# An efficient grid layout algorithm for biological networks utilizing various biological attributes

**DOI:** 10.1186/1471-2105-8-76

**Published:** 2007-03-06

**Authors:** Kaname Kojima, Masao Nagasaki, Euna Jeong, Mitsuru Kato, Satoru Miyano

**Affiliations:** 1 Human Genome Center, Institute of Medical Science, University of Tokyo, 4-6-1 Shirokanedai, Minato-ku, Tokyo 108-8639, Japan

## Abstract

**Background:**

Clearly visualized biopathways provide a great help in understanding biological systems. However, manual drawing of large-scale biopathways is time consuming. We proposed a grid layout algorithm that can handle gene-regulatory networks and signal transduction pathways by considering edge-edge crossing, node-edge crossing, distance measure between nodes, and subcellular localization information from Gene Ontology. Consequently, the layout algorithm succeeded in drastically reducing these crossings in the apoptosis model. However, for larger-scale networks, we encountered three problems: (i) the initial layout is often very far from any local optimum because nodes are initially placed at random, (ii) from a biological viewpoint, human layouts still exceed automatic layouts in understanding because except subcellular localization, it does not fully utilize biological information of pathways, and (iii) it employs a local search strategy in which the neighborhood is obtained by moving one node at each step, and automatic layouts suggest that simultaneous movements of multiple nodes are necessary for better layouts, while such extension may face worsening the time complexity.

**Results:**

We propose a new grid layout algorithm. To address problem (i), we devised a new force-directed algorithm whose output is suitable as the initial layout. For (ii), we considered that an appropriate alignment of nodes having the same biological attribute is one of the most important factors of the comprehension, and we defined a new score function that gives an advantage to such configurations. For solving problem (iii), we developed a search strategy that considers swapping nodes as well as moving a node, while keeping the order of the time complexity. Though a naïve implementation increases by one order, the time complexity, we solved this difficulty by devising a method that caches differences between scores of a layout and its possible updates.

**Conclusion:**

Layouts of the new grid layout algorithm are compared with that of the previous algorithm and human layout in an endothelial cell model, three times as large as the apoptosis model. The total cost of the result from the new grid layout algorithm is similar to that of the human layout. In addition, its convergence time is drastically reduced (40% reduction).

## Background

Modeling and simulations of large scale biological pathways are some of the most important tasks in Bioinformatics. Many applications, e.g., Cell Illustrator [1,2], Cytoscape [3], Pajek [4], PATIKA [5,6], and CADLIVE [7,8] have been developed in this area. Related to these topics, the visualization of biopathways is considered to play a key role in understanding biological systems. However, manual drawing of large-scale biopathways is a time consuming work, hence suitable biopathway layout algorithms and their applications are strongly demanded.

Biopathways are categorized into three types, i.e., metabolic pathways, signal transduction pathways, and gene-regulatory networks. For metabolic pathways, several algorithms have been already proposed [9-13], and some of them succeeded in capturing the flow of the reactions well. In contrast, few layout algorithms that provide a convenient biological understanding have been proposed for signal transduction pathways [14,15] and gene-regulatory networks [16,17]. Thus, our new layout algorithm is focused on signal transduction pathways and gene-regulatory networks. For signal transduction pathways and gene-regulatory networks, extant layout algorithms can be categorized into two types; force-directed and grid layout algorithms.

Force-directed algorithms are used in [16,17] by taking into account the directional constraint following different types of molecular and simple regional constraints from subcellular localizations. These algorithms have been successfully integrated into PATIKA. However, as pointed out in [14], force-directed algorithms may not be suitable for compact layouts of complex biopathways. Furthermore, intricately shaped regions such as torus-shaped region cannot be handled well as regional constraints in these force-directed algorithms. Hence, they are not suitable for models containing torus-shaped plasma membrane and nuclear membrane although such types of models are common as biopathways.

A grid layout algorithm (referred to as LK-grid layout algorithm) was initially proposed by Li and Kurata. The grid layout algorithm restricts the positions of all nodes to grid points. Li and Kurata defined a cost function for two nodes that depends on some distance between these nodes and the topology of their connections in the graph. They applied LK-grid layout algorithm to a yeast cell-cycle pathway and concluded that this algorithm can geometrically classify the pathway into functional categories without using biological information. Moreover, they noticed that the algorithm generates compact layouts while avoiding overlaps between nodes. [15] proposed CB-grid layout algorithm, in which so as to reduce edge-edge crossings and node-edge crossings, a penalty for these cases is added to the cost function. The algorithm can also deal with any complex regional constraints following subcellular localizations, and besides search space is reduced due to these constrains. As a result, in the apoptosis model, the layout algorithm succeeded in a drastic reduction of edge-edge crossings and node-edge crossings, while placing nodes in biologically proper regions.

However, in the case of larger-scale networks, this algorithm encountered three problems. First, a layout with randomly placed nodes is used as the initial layout. This random layout contains a large number of edge-edge crossings and node-edge crossings; subsequently, many iterations will be required to obtain a locally optimal layout. Secondly, although one of the features of CB-grid layout algorithm is to use the subcellular localization information, it still does not fully utilize biological characteristics. For example, it does not consider such biological attributes as types of entities (protein, mRNA, and microRNA) or types of processes (phosphorylation, binding, and translation), although in human layouts these biological attributes are apt to contribute to the comprehension of interesting biopathways easier. Thirdly, according to a greedy strategy, CB-grid layout algorithm updates a layout by moving one node at each step until the layout reaches an optimum. However, resulting layouts are just local optima, hence their quality fundamentally depends on the initial layout. Although in [15] a multi-step CB-grid layout algorithm was also proposed to solve this drawback, it requires higher time complexity and hence is not suitable for practical applications.

To overcome these three problems, we propose a new grid layout algorithm. For the first problem, we propose a new force-directed algorithm whose output is suitable as the initial layout of grid layout algorithms. For the second problem, we introduce the concept that assigns a score i.e., a negative cost, to a layout depending on how nodes with the same attribute are aligned. This concept is realized with a combo score function, which is combined with the cost function defined in CB-grid layout algorithm. For the third problem, the search strategy in CB-grid layout algorithm is improved by adding the swap operation while keeping the time complexity. By the swap operation, the new grid layout can also consider layouts generated by exchanging the positions of two nodes in the current layout at each step.

The Methods section is organized as follows: (i) first, we introduce the previous grid layout, i.e., CB-grid layout algorithm; (ii) for the first improvement in the initial layout of CB-grid layout algorithm, the new force-directed algorithm termed Eades initial layout algorithm is described; (iii) for the second improvement, CCB-grid layout algorithm, which is CB-grid layout algorithm with the combo score function is described; (iv) for the third improvement, SCCB-grid layout algorithm, which enhances CCB-grid layout algorithm by adding the swap operation is presented. In the Results and Discussion section, the performances of these new algorithms are compared and verified by applying them to the signal transduction pathway of an endothelial cell, which is larger than the pathways in [14] and [15].

## Methods

### CB-grid layout algorithm: Introduction of the grid layout algorithm

Given a graph *G *= (*V*, *E*) with nodes *V *and edges *E*, a *layout L *= (*V*, *E*, *U*, *P*) of *G *consists of the underlying graph *G*, grid points *U *and a function *P *: *V *→ *U *such that *P *(*v*_*α*_) ≠ *P *(*v*_*β*_) for any two distinct nodes *v*_*α*_, *v*_*β *_∈ *V*. This definition does not allow overlaps between nodes in the layout. For a layout *L*, this paper uses the following notations.

• *W*_*L*_: a set of vacant points of *L*.

• *E*_*v*_: the set of all edges connected to node *v*.

• |*V*|: the number of nodes in *V*.

• |*W*|: the number of vacant points in *L*, instead of |*W*_*L*_| if there is no confusion possible.

We define the following operations.

• *T*_*v *→ *p *_*L*: the layout generated by moving a node *v *to a vacant point *p *∈ *W*_*L*_.

• Svα↔vβ
 MathType@MTEF@5@5@+=feaafiart1ev1aaatCvAUfKttLearuWrP9MDH5MBPbIqV92AaeXatLxBI9gBaebbnrfifHhDYfgasaacH8akY=wiFfYdH8Gipec8Eeeu0xXdbba9frFj0=OqFfea0dXdd9vqai=hGuQ8kuc9pgc9s8qqaq=dirpe0xb9q8qiLsFr0=vr0=vr0dc8meaabaqaciaacaGaaeqabaqabeGadaaakeaacqWGtbWudaWgaaWcbaGaemODay3aaSbaaWqaaGGaciab=f7aHbqabaWccqGHugYQcqWG2bGDdaWgaaadbaGae8NSdigabeaaaSqabaaaaa@368F@*L*: the layout generated by swapping nodes *v*_*α *_and *v*_*β*_.

• *D*_*v *_*L*: the layout generated by removing a node *v *and all edges connected to *v*.

In addition, we define the following functions.

• Crossei,ej
 MathType@MTEF@5@5@+=feaafiart1ev1aaatCvAUfKttLearuWrP9MDH5MBPbIqV92AaeXatLxBI9gBaebbnrfifHhDYfgasaacH8akY=wiFfYdH8Gipec8Eeeu0xXdbba9frFj0=OqFfea0dXdd9vqai=hGuQ8kuc9pgc9s8qqaq=dirpe0xb9q8qiLsFr0=vr0=vr0dc8meaabaqaciaacaGaaeqabaqabeGadaaakeaacqWGdbWqcqWGYbGCcqWGVbWBcqWGZbWCcqWGZbWCdaWgaaWcbaGaemyzau2aaSbaaWqaaiabdMgaPbqabaWccqGGSaalcqWGLbqzdaWgaaadbaGaemOAaOgabeaaaSqabaaaaa@3A47@ (*L*): a binary function that returns 1 if an edge *e*_*i *_crosses with an edge *e*_*j *_and 0 otherwise.

• Crossvi,ej
 MathType@MTEF@5@5@+=feaafiart1ev1aaatCvAUfKttLearuWrP9MDH5MBPbIqV92AaeXatLxBI9gBaebbnrfifHhDYfgasaacH8akY=wiFfYdH8Gipec8Eeeu0xXdbba9frFj0=OqFfea0dXdd9vqai=hGuQ8kuc9pgc9s8qqaq=dirpe0xb9q8qiLsFr0=vr0=vr0dc8meaabaqaciaacaGaaeqabaqabeGadaaakeaacqWGdbWqcqWGYbGCcqWGVbWBcqWGZbWCcqWGZbWCdaWgaaWcbaGaemODay3aaSbaaWqaaiabdMgaPbqabaWccqGGSaalcqWGLbqzdaWgaaadbaGaemOAaOgabeaaaSqabaaaaa@3A69@ (*L*): a binary function that returns 1 if an edge *e*_*j *_crosses with a node *v*_*i *_and 0 otherwise.

• Distancevi,vj
 MathType@MTEF@5@5@+=feaafiart1ev1aaatCvAUfKttLearuWrP9MDH5MBPbIqV92AaeXatLxBI9gBaebbnrfifHhDYfgasaacH8akY=wiFfYdH8Gipec8Eeeu0xXdbba9frFj0=OqFfea0dXdd9vqai=hGuQ8kuc9pgc9s8qqaq=dirpe0xb9q8qiLsFr0=vr0=vr0dc8meaabaqaciaacaGaaeqabaqabeGadaaakeaaieGacqWFebarcqWFPbqAcqWFZbWCcqWF0baDcqWFHbqycqWFUbGBcqWFJbWycqWFLbqzdaWgaaWcbaGaemODay3aaSbaaWqaaiabdMgaPbqabaWccqGGSaalcqWG2bGDdaWgaaadbaGaemOAaOgabeaaaSqabaaaaa@3E53@ (*L*): a function that returns wvi,vj⋅md(vi,vj)
 MathType@MTEF@5@5@+=feaafiart1ev1aaatCvAUfKttLearuWrP9MDH5MBPbIqV92AaeXatLxBI9gBaebbnrfifHhDYfgasaacH8akY=wiFfYdH8Gipec8Eeeu0xXdbba9frFj0=OqFfea0dXdd9vqai=hGuQ8kuc9pgc9s8qqaq=dirpe0xb9q8qiLsFr0=vr0=vr0dc8meaabaqaciaacaGaaeqabaqabeGadaaakeaacqWG3bWDdaWgaaWcbaGaemODay3aaSbaaWqaaiabdMgaPbqabaWccqGGSaalcqWG2bGDdaWgaaadbaGaemOAaOgabeaaaSqabaGccqGHflY1cqWGTbqBcqWGKbazcqGGOaakcqWG2bGDdaWgaaWcbaGaemyAaKgabeaakiabcYcaSiabdAha2naaBaaaleaacqWGQbGAaeqaaOGaeiykaKcaaa@42E9@, where wvi,vj
 MathType@MTEF@5@5@+=feaafiart1ev1aaatCvAUfKttLearuWrP9MDH5MBPbIqV92AaeXatLxBI9gBaebbnrfifHhDYfgasaacH8akY=wiFfYdH8Gipec8Eeeu0xXdbba9frFj0=OqFfea0dXdd9vqai=hGuQ8kuc9pgc9s8qqaq=dirpe0xb9q8qiLsFr0=vr0=vr0dc8meaabaqaciaacaGaaeqabaqabeGadaaakeaacqWG3bWDdaWgaaWcbaGaemODay3aaSbaaWqaaiabdMgaPbqabaWccqGGSaalcqWG2bGDdaWgaaadbaGaemOAaOgabeaaaSqabaaaaa@3541@ is the weight to the couple of nodes *v*_*i *_and *v*_*j*_, and *md *(*v*_*i*_, *v*_*j*_) is the Manhattan distance between *v*_*i *_and *v*_*j*_.

In our previous approach [15] (mainly referred to as CB-grid layout algorithm), the *layout cost C *(*L*) of *L *was defined as follows:

C(L)=Wee∑ei,ej∈ECrossei,ej(L)+Wne∑vk∈V,el∈ECrossvk,el(L)+Wdc∑vm,vn∈VDistancevm,vn(L),     (1)
 MathType@MTEF@5@5@+=feaafiart1ev1aaatCvAUfKttLearuWrP9MDH5MBPbIqV92AaeXatLxBI9gBaebbnrfifHhDYfgasaacH8akY=wiFfYdH8Gipec8Eeeu0xXdbba9frFj0=OqFfea0dXdd9vqai=hGuQ8kuc9pgc9s8qqaq=dirpe0xb9q8qiLsFr0=vr0=vr0dc8meaabaqaciaacaGaaeqabaqabeGadaaakeaacqWGdbWqcqGGOaakcqWGmbatcqGGPaqkcqGH9aqpcqWGxbWvdaWgaaWcbaGaemyzauMaemyzaugabeaakmaaqafabaGaem4qamKaemOCaiNaem4Ba8Maem4CamNaem4Cam3aaSbaaSqaaiabdwgaLnaaBaaameaacqWGPbqAaeqaaSGaeiilaWIaemyzau2aaSbaaWqaaiabdQgaQbqabaaaleqaaaqaaiabdwgaLnaaBaaameaacqWGPbqAaeqaaSGaeiilaWIaemyzau2aaSbaaWqaaiabdQgaQbqabaWccqGHiiIZcqWGfbqraeqaniabggHiLdGccqGGOaakcqWGmbatcqGGPaqkcqGHRaWkcqWGxbWvdaWgaaWcbaGaemOBa4MaemyzaugabeaakmaaqafabaGaem4qamKaemOCaiNaem4Ba8Maem4CamNaem4Cam3aaSbaaSqaaiabdAha2naaBaaameaacqWGRbWAaeqaaSGaeiilaWIaemyzau2aaSbaaWqaaiabdYgaSbqabaaaleqaaaqaaiabdAha2naaBaaameaacqWGRbWAaeqaaSGaeyicI4SaemOvayLaeiilaWIaemyzau2aaSbaaWqaaiabdYgaSbqabaWccqGHiiIZcqWGfbqraeqaniabggHiLdGccqGGOaakcqWGmbatcqGGPaqkcqGHRaWkcqWGxbWvdaWgaaWcbaGaemizaqMaem4yamgabeaakmaaqafabaacbiGae8hraqKae8xAaKMae83CamNae8hDaqNae8xyaeMae8NBa4Mae83yamMae8xzau2aaSbaaSqaaiabdAha2naaBaaameaacqWGTbqBaeqaaSGaeiilaWIaemODay3aaSbaaWqaaiabd6gaUbqabaaaleqaaaqaaiabdAha2naaBaaameaacqWGTbqBaeqaaSGaeiilaWIaemODay3aaSbaaWqaaiabd6gaUbqabaWccqGHiiIZcqWGwbGvaeqaniabggHiLdGccqGGOaakcqWGmbatcqGGPaqkcqGGSaalcaWLjaGaaCzcamaabmaabaGaeGymaedacaGLOaGaayzkaaaaaa@9F58@

where *W*_*ee*_, *W*_*ne*_, and *W*_*d *_are called respectively *edge-edge crossing weight, node-edge crossing weight*, and *distance cost weight*.

The CB-grid layout algorithm repeats the operation of moving a unique node to a vacant point one-by-one until it reaches a locally optimal layout. At each step, the algorithm calculates costs of all layouts that can be generated by moving one of all nodes to one of all vacant points. The layout with the lowest cost is selected as a starting layout for the next step. After reaching convergence, the algorithm outputs a locally optimal layout. If the cost calculation of all possible adjacent layouts is implemented in a naïve way, high time complexity is required. To overcome this problem, the previous method [15] introduced Δ matrix that stores each possible cost difference at the previous step and succeeded in reducing the time complexity at each step from *O *(|*W*| (|*V*|^2 ^+ |*E*|^2^) to *O *(|*V*|^2 ^+ |*E*|^2 ^+ |*W*||Evβ
 MathType@MTEF@5@5@+=feaafiart1ev1aaatCvAUfKttLearuWrP9MDH5MBPbIqV92AaeXatLxBI9gBaebbnrfifHhDYfgasaacH8akY=wiFfYdH8Gipec8Eeeu0xXdbba9frFj0=OqFfea0dXdd9vqai=hGuQ8kuc9pgc9s8qqaq=dirpe0xb9q8qiLsFr0=vr0=vr0dc8meaabaqaciaacaGaaeqabaqabeGadaaakeaacqWGfbqrdaWgaaWcbaGaemODay3aaSbaaWqaaGGaciab=j7aIbqabaaaleqaaaaa@3140@| (|*V*| + |*E*|)), where *v*_*β *_is the node moved at the previous step.

When CB-grid layout algorithm was applied to several biopathways, we encountered three problems. Thus, we propose new grid layout algorithms that solve these problems. Problems and solutions are summarized as follows:

1. Improving the choice of the initial layout: since a locally optimal layout depends noticeably on the initial layout, we first apply Eades initial layout algorithm to a random layout, and use its output as the initial layout. In the previous approach, a random layout was directly used as the initial layout.

2. Improving the cost function: we introduce the concept of a combo score that gives a good score, i.e., a negative cost when nodes with the same biological attribute are aligned (CCB-grid layout algorithm). In CB-grid layout algorithm, the biological attributes, except subcellular localization, were ignored.

3. Improving the search strategy: we propose a better search strategy, which allows us to obtain improved results, keeping the time complexity. For obtaining a better layout, the search space is extended by adding the swap operation. At each step, all layouts obtained by swapping two nodes are also considered (SCCB-grid layout algorithm).

In the remainder of this section, we describe these three new algorithms mentioned above.

### Eades initial layout algorithm: generating a new initial layout for grid layout algorithms

In the previous paper [15], a random layout was used as an initial layout for CB-grid layout algorithm. When the initial layout is far from the global optimum, the local optimum obtained tends to be unacceptable. Therefore, we decided to develop Eades algorithm [18] and use its output as the initial layout. Eades algorithm is one of the force-directed algorithms, consisting of the following two steps.

1. Two types of forces are defined for each pair of nodes. If two nodes are adjacent, there exists an attractive force *a*_*c*1 _log(*d*/*a*_*c*2_) between them, where *a*_*c*1 _and *a*_*c*2 _are constants, and *d *is the distance between the two nodes. On the other hand, if two nodes are not adjacent, there exists a repulsive force *r*_*c*_/d
 MathType@MTEF@5@5@+=feaafiart1ev1aaatCvAUfKttLearuWrP9MDH5MBPbIqV92AaeXatLxBI9gBaebbnrfifHhDYfgasaacH8akY=wiFfYdH8Gipec8Eeeu0xXdbba9frFj0=OqFfea0dXdd9vqai=hGuQ8kuc9pgc9s8qqaq=dirpe0xb9q8qiLsFr0=vr0=vr0dc8meaabaqaciaacaGaaeqabaqabeGadaaakeaadaGcaaqaaiabdsgaKbWcbeaaaaa@2E18@ between them, where *r*_*c *_is a constant. At each step, the positions of all the nodes are updated according to the sum of the repulsive and attractive forces between them.

2. The above step is iterated a predetermined number of times, and the final result is obtained.

We have customized two points in Eades algorithm. First, nodes in Eades algorithm can be placed anywhere. All the nodes in the initial layout for CB-grid layout algorithm, however, should be placed on the grid points that satisfy the subcellular localization. Thus, the output of Eades algorithm cannot be used directly as an input for CB-grid layout algorithm.

To handle this problem, we propose to move each node to the closest vacant point that satisfies the subcellular localization after moving nodes at each step.

**Figure 1:**
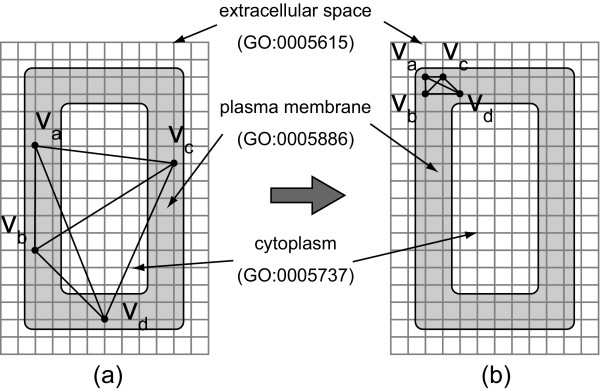
**Two layouts with the same canvas and three subcellular localizations**. The grid canvases (a) and (b) have the same biological subcellular localizations extracellular space, plasma membrane, and cytoplasm. Both canvases contain the same graph with four nodes that are located in plasma membrane, which surrounds cytoplasm. In (a), nodes are spread apart in plasma membrane, and edges among these nodes cross over cytoplasm. In (b), the nodes are gathered in the left-top corner, and no edge crosses over cytoplasm. Due to its crossing patterns in (a) these edges have a higher probability to cross other nodes in cytoplasm. This is the drawback of using the layout in (a) as the initial layout for Eades initial layout algorithm.

Second improvement is the following one. Since Eades algorithm doesn't consider edge-edge crossings and node-edge crossings in its implementation, the resulting layout could contain a lot of such crossings. For example, suppose a biological pathway with a subcellular localization, membrane, which slimly surrounds other subcellular localizations as shown in Figure 1(a), the graph in (a) could be a layout resulting from Eades algorithm. In this case, the layout might contain a large number of edge-edge crossings and node-edge crossings because edges cross over other subcellular localizations. In order to avoid this problem, we propose to gather nodes around a particular grid point for each subcellular localization as shown in Figure 1(b). Eades algorithm with the above improvements is called *Eades initial layout algorithm*.

### CCB-grid layout algorithm: utilizing various biological attributes

**Figure 2:**
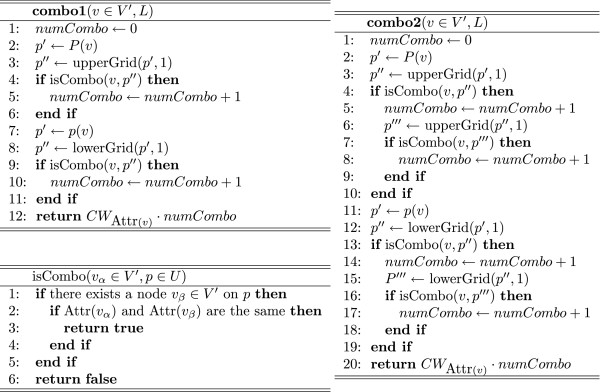
**Pseudo codes of combo score functions: combo1 and combo2**. (a) **combo1**: a score function that considers nodes with one vertical grid distance from the target node. (b) **combo2**: a score function that considers nodes with up to two vertical grid distances from the target node, (c) isCombo: a boolean function that takes a node and a grid point as its arguments and returns "true" if the attribute of the node and that of the node on the grid point are the same.

When humans draw biopathway models, nodes with the same attribute are usually arranged according to a rule. In CB-grid layout algorithm, this type of information is completely ignored. To implement this type of property, we introduce the concept of combo scores called **combo1 **and **combo2 **(see Figure 2). Note that a combo score is applied only to nodes having an attribute since some nodes do not have any attributes. We denote the set of nodes having an attribute by *V' *⊆ *V*. In this algorithm, (i) upperGrid(*p*, *i*)/lowerGrid(*p*, *i*) returns the upper/lower *i*th grid point over/under a grid point *p *∈ *P*, and (ii) Attr(*v*) is the attribute of a node *v *∈ *V'*, and *CW*_*a *_= (1 + *C*/|V′a
 MathType@MTEF@5@5@+=feaafiart1ev1aaatCvAUfKttLearuWrP9MDH5MBPbIqV92AaeXatLxBI9gBaebbnrfifHhDYfgasaacH8akY=wiFfYdH8Gipec8Eeeu0xXdbba9frFj0=OqFfea0dXdd9vqai=hGuQ8kuc9pgc9s8qqaq=dirpe0xb9q8qiLsFr0=vr0=vr0dc8meaabaqaciaacaGaaeqabaqabeGadaaakeaacuWGwbGvgaqbamaaBaaaleaacqWGHbqyaeqaaaaa@2F64@|), where *C *is a constant and normally set to |*V*|, and V′a
 MathType@MTEF@5@5@+=feaafiart1ev1aaatCvAUfKttLearuWrP9MDH5MBPbIqV92AaeXatLxBI9gBaebbnrfifHhDYfgasaacH8akY=wiFfYdH8Gipec8Eeeu0xXdbba9frFj0=OqFfea0dXdd9vqai=hGuQ8kuc9pgc9s8qqaq=dirpe0xb9q8qiLsFr0=vr0=vr0dc8meaabaqaciaacaGaaeqabaqabeGadaaakeaacuWGwbGvgaqbamaaBaaaleaacqWGHbqyaeqaaaaa@2F64@ is the set of nodes having an attribute *a*.

**Figure 3:**
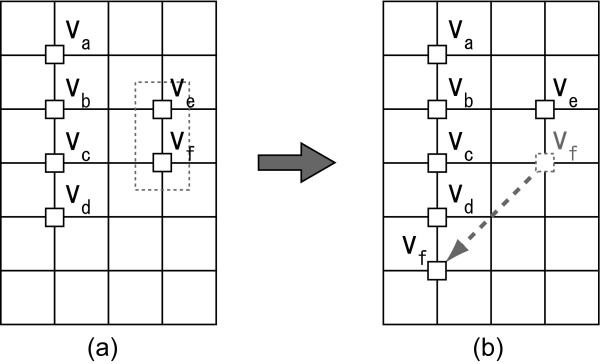
**An example that compares the features of combo1 and combo2 score functions**. (a) An intermediate layout of CCB-grid layout algorithm. In this layout, all six nodes have the same attribute. (b) The next candidate layout that is generated from (a) by moving node *v*_*f *_below node *v*_*d*_. Combo scores of (a) and (b) are the same with **combo1 **score function. Instead, the combo score of (b) will be better than (a) with **combo2 **score function.

The combo score is designed such that the more nodes with the same attribute are aligned vertically, the higher the score is. The combo score is defined between two nodes, and a combo score of a layout *L *is defined to be the sum of all the combo scores occurring in *L*. We say that two nodes have a *combo relation *when a combo score occurs between them. Note that the horizontal alignment score is not implemented because if the above combo score supported both the vertical and horizontal directions, the numbers of edge-edge crossings and node-edge crossings would be considerably increased. Therefore, we should choose only one direction for combo scores. In this paper, we defined combo scores in the vertical direction. We have considered two types of combo scores, i.e., **combo1 **and **combo2 **for layouts in Figure 3(a) and 3(b), respectively. Let nodes *v*_*a *_to *v*_*f *_in Figure 3 have the same attribute. The **combo1 **considers only the nodes with one vertical grid distance from the target node. In contrast, **combo2 **considers the nodes with up to two vertical grid distances from the target node. For the layout in Figure 3(a), the number of combo relations with **combo1 **and **combo2 **are 8 and 12, respectively. If node *v*_*f *_is moved as shown in Figure 3(b), the number of combo relations with **combo1 **is the same as before, whereas that with **combo2 **is 14. Thus, only by using **combo2**, we can improve the combo score when node *v*_*f *_is moved as shown in Figure 3(a) and 3(b). As shown in the dotted rectangle in Figure 3(a), a pair of vertically aligned nodes often occurs during the process of updating a layout. In this case, Figure 3(b) should be a better layout than Figure 3(a). For this reason, we decide to employ **combo2**. Henceforth, for a node *v *∈ *V *in a layout *L*, *Combo*_*v *_(*L*) denotes the same combo score as **combo2 **(*v*, *L*). The total score ∑v∈VCombov(L)
 MathType@MTEF@5@5@+=feaafiart1ev1aaatCvAUfKttLearuWrP9MDH5MBPbIqV92AaeXatLxBI9gBaebbnrfifHhDYfgasaacH8akY=wiFfYdH8Gipec8Eeeu0xXdbba9frFj0=OqFfea0dXdd9vqai=hGuQ8kuc9pgc9s8qqaq=dirpe0xb9q8qiLsFr0=vr0=vr0dc8meaabaqaciaacaGaaeqabaqabeGadaaakeaadaaeqbqaaiabdoeadjabd+gaVjabd2gaTjabdkgaIjabd+gaVnaaBaaaleaacqWG2bGDaeqaaOGaeiikaGIaemitaWKaeiykaKcaleaacqWG2bGDcqGHiiIZcqWGwbGvaeqaniabggHiLdaaaa@3E08@ for *L *is denoted by *Combo *(*L*).

If *CW*_*a *_returns the same value for any attribute *a*, many of the nodes with the same attribute will be vertically aligned easily since they have a greater chance to neighbor one another. So as to reduce the biases among the attributes, we define *CW*_*a *_to be inversely related to the total number of the nodes whose attribute is *a*.

By modifying the layout score of CB-grid layout algorithm, we can define the layout cost *C *(*L*) of a layout *L *with the new concept of the combo score as follows:

C(L)=Wee∑ei,ej∈ECrossei,ej(L)+Wne∑vk∈V,el∈ECrossvk,el(L)+Wdc∑vm,vn∈VDistancevm,vn(L)−Wcs(12∑vo∈V′Combovo(L)),     (2)
 MathType@MTEF@5@5@+=feaafiart1ev1aaatCvAUfKttLearuWrP9MDH5MBPbIqV92AaeXatLxBI9gBaebbnrfifHhDYfgasaacH8akY=wiFfYdH8Gipec8Eeeu0xXdbba9frFj0=OqFfea0dXdd9vqai=hGuQ8kuc9pgc9s8qqaq=dirpe0xb9q8qiLsFr0=vr0=vr0dc8meaabaqaciaacaGaaeqabaqabeGadaaakeaafaqaaeGadaaabaGaem4qamKaeiikaGIaemitaWKaeiykaKcabaGaeyypa0dabaGaem4vaC1aaSbaaSqaaiabdwgaLjabdwgaLbqabaGcdaaeqbqaaiabdoeadjabdkhaYjabd+gaVjabdohaZjabdohaZnaaBaaaleaacqWGLbqzdaWgaaadbaGaemyAaKgabeaaliabcYcaSiabdwgaLnaaBaaameaacqWGQbGAaeqaaaWcbeaaaeaacqWGLbqzdaWgaaadbaGaemyAaKgabeaaliabcYcaSiabdwgaLnaaBaaameaacqWGQbGAaeqaaSGaeyicI4SaemyraueabeqdcqGHris5aOGaeiikaGIaemitaWKaeiykaKIaey4kaSIaem4vaC1aaSbaaSqaaiabd6gaUjabdwgaLbqabaGcdaaeqbqaaiabdoeadjabdkhaYjabd+gaVjabdohaZjabdohaZnaaBaaaleaacqWG2bGDdaWgaaadbaGaem4AaSgabeaaliabcYcaSiabdwgaLnaaBaaameaacqWGSbaBaeqaaaWcbeaaaeaacqWG2bGDdaWgaaadbaGaem4AaSgabeaaliabgIGiolabdAfawjabcYcaSiabdwgaLnaaBaaameaacqWGSbaBaeqaaSGaeyicI4SaemyraueabeqdcqGHris5aOGaeiikaGIaemitaWKaeiykaKIaey4kaSIaem4vaC1aaSbaaSqaaiabdsgaKjabdogaJbqabaGcdaaeqbqaaGqaciab=reaejab=LgaPjab=nhaZjab=rha0jab=fgaHjab=5gaUjab=ngaJjab=vgaLnaaBaaaleaacqWG2bGDdaWgaaadbaGaemyBa0gabeaaliabcYcaSiabdAha2naaBaaameaacqWGUbGBaeqaaaWcbeaaaeaacqWG2bGDdaWgaaadbaGaemyBa0gabeaaliabcYcaSiabdAha2naaBaaameaacqWGUbGBaeqaaSGaeyicI4SaemOvayfabeqdcqGHris5aOGaeiikaGIaemitaWKaeiykaKcabaaabaGaeyOeI0cabaGaem4vaC1aaSbaaSqaaiabdogaJjabdohaZbqabaGcdaqadaqaamaalaaabaGaeGymaedabaGaeGOmaidaamaaqafabaGaem4qamKaem4Ba8MaemyBa0MaemOyaiMaem4Ba82aaSbaaSqaaiabdAha2naaBaaameaacqWGVbWBaeqaaaWcbeaakiabcIcaOiabdYeamjabcMcaPaWcbaGaemODay3aaSbaaWqaaiabd+gaVbqabaWccqGHiiIZcuWGwbGvgaqbaaqab0GaeyyeIuoaaOGaayjkaiaawMcaaiabcYcaSaaacaWLjaGaaCzcamaabmaabaGaeGOmaidacaGLOaGaayzkaaaaaa@BCAF@

where *W*_*cs *_is called *combo score weight*. CB-grid layout algorithm improved by the above modification is named *Combo score, Cross cost and Biological information grid layout algorithm *(CCB-grid layout algorithm). The reason for multiplying the sum of the combo scores by 1/2 is that combo scores are counted twice since a combo score between nodes *v*_*α *_and *v*_*β *_is included in both Combovα
 MathType@MTEF@5@5@+=feaafiart1ev1aaatCvAUfKttLearuWrP9MDH5MBPbIqV92AaeXatLxBI9gBaebbnrfifHhDYfgasaacH8akY=wiFfYdH8Gipec8Eeeu0xXdbba9frFj0=OqFfea0dXdd9vqai=hGuQ8kuc9pgc9s8qqaq=dirpe0xb9q8qiLsFr0=vr0=vr0dc8meaabaqaciaacaGaaeqabaqabeGadaaakeaacqWGdbWqcqWGVbWBcqWGTbqBcqWGIbGycqWGVbWBdaWgaaWcbaGaemODay3aaSbaaWqaaGGaciab=f7aHbqabaaaleqaaaaa@36B8@ (*L*) and Combovβ
 MathType@MTEF@5@5@+=feaafiart1ev1aaatCvAUfKttLearuWrP9MDH5MBPbIqV92AaeXatLxBI9gBaebbnrfifHhDYfgasaacH8akY=wiFfYdH8Gipec8Eeeu0xXdbba9frFj0=OqFfea0dXdd9vqai=hGuQ8kuc9pgc9s8qqaq=dirpe0xb9q8qiLsFr0=vr0=vr0dc8meaabaqaciaacaGaaeqabaqabeGadaaakeaacqWGdbWqcqWGVbWBcqWGTbqBcqWGIbGycqWGVbWBdaWgaaWcbaGaemODay3aaSbaaWqaaGGaciab=j7aIbqabaaaleqaaaaa@36BA@ (*L*). The algorithm is the same as *C-optimization *(*L*) step in [15] except for the use of the above layout cost *C *(*L*), i.e., the algorithm for calculating Δ matrix is also the same.

For calculating the combo score for each node, only four nodes need to be checked at most, i.e., its time complexity is constant, while for calculating the edge-edge crossing cost, the node-edge crossing cost, and the distance cost for each node, these time complexities depend on |*E*|, |*V*|, and |*W*|, respectively. Thus, without using Δ matrix, the time complexity related to combo scores is *O *(|*V*||*W*|) at each step.

At each step, we need to calculate the difference between the combo score of the previous layout *L *and that of the current layout that is generated by moving a node *v *to a vacant point *p*, i.e., *Combo*(*T*_*v*→*p *_*L*) – *Combo*(*L*). We can efficiently calculate the difference of the combo score Δvpcs
 MathType@MTEF@5@5@+=feaafiart1ev1aaatCvAUfKttLearuWrP9MDH5MBPbIqV92AaeXatLxBI9gBaebbnrfifHhDYfgasaacH8akY=wiFfYdH8Gipec8Eeeu0xXdbba9frFj0=OqFfea0dXdd9vqai=hGuQ8kuc9pgc9s8qqaq=dirpe0xb9q8qiLsFr0=vr0=vr0dc8meaabaqaciaacaGaaeqabaqabeGadaaakeaacqqHuoardaqhaaWcbaGaemODayNaemiCaahabaGaem4yamMaem4Camhaaaaa@33DB@ (*L*) as follows:

Δvpcs(L)={Wcs(Combov(Tv→pL)−Combov(L)+Adjv(Tv→pL)−Adjv(L))if v∈V′0ifv∉V′,     (3)
 MathType@MTEF@5@5@+=feaafiart1ev1aaatCvAUfKttLearuWrP9MDH5MBPbIqV92AaeXatLxBI9gBaebbnrfifHhDYfgasaacH8akY=wiFfYdH8Gipec8Eeeu0xXdbba9frFj0=OqFfea0dXdd9vqai=hGuQ8kuc9pgc9s8qqaq=dirpe0xb9q8qiLsFr0=vr0=vr0dc8meaabaqaciaacaGaaeqabaqabeGadaaakeaacqqHuoardaqhaaWcbaGaemODayNaemiCaahabaGaem4yamMaem4CamhaaOGaeiikaGIaemitaWKaeiykaKIaeyypa0ZaaiqabeaafaqabeGacaaabaGaem4vaC1aaSbaaSqaaiabdogaJjabdohaZbqabaGccqGGOaakcqWGdbWqcqWGVbWBcqWGTbqBcqWGIbGycqWGVbWBdaWgaaWcbaGaemODayhabeaakiabcIcaOiabdsfaunaaBaaaleaacqWG2bGDcqGHsgIRcqWGWbaCaeqaaOGaemitaWKaeiykaKIaeyOeI0Iaem4qamKaem4Ba8MaemyBa0MaemOyaiMaem4Ba82aaSbaaSqaaiabdAha2bqabaGccqGGOaakcqWGmbatcqGGPaqkcqGHRaWkcqWGbbqqcqWGKbazcqWGQbGAdaWgaaWcbaGaemODayhabeaakiabcIcaOiabdsfaunaaBaaaleaacqWG2bGDcqGHsgIRcqWGWbaCaeqaaOGaemitaWKaeiykaKIaeyOeI0IaemyqaeKaemizaqMaemOAaO2aaSbaaSqaaiabdAha2bqabaGccqGGOaakcqWGmbatcqGGPaqkcqGGPaqkaeaaieaacqWFPbqAcqWFMbGzcqqGGaaicqWG2bGDcqGHiiIZcuWGwbGvgaqbaaqaaiabicdaWaqaaiab=LgaPjab=zgaMjab=bcaGiabdAha2jabgMGiplqbdAfawzaafaaaaaGaay5EaaGaeiilaWIaaCzcaiaaxMaadaqadaqaaiabiodaZaGaayjkaiaawMcaaaaa@8954@

where

Adjv={CWAttr(v)if isCombo(v,upperGrid(P(v,1)))=true&isCombo(v,lowerGrid(P(v,1)))=true0otherwise..     (4)
 MathType@MTEF@5@5@+=feaafiart1ev1aaatCvAUfKttLearuWrP9MDH5MBPbIqV92AaeXatLxBI9gBaebbnrfifHhDYfgasaacH8akY=wiFfYdH8Gipec8Eeeu0xXdbba9frFj0=OqFfea0dXdd9vqai=hGuQ8kuc9pgc9s8qqaq=dirpe0xb9q8qiLsFr0=vr0=vr0dc8meaabaqaciaacaGaaeqabaqabeGadaaakeaacqWGbbqqcqWGKbazcqWGQbGAdaWgaaWcbaGaemODayhabeaakiabg2da9maaceqabaqbaeqabiGaaaqaaiabdoeadjabdEfaxnaaBaaaleaacqqGbbqqcqqG0baDcqqG0baDcqqGYbGCcqGGOaakcqWG2bGDcqGGPaqkaeqaaaGcbaGaeeyAaKMaeeOzayMaeeiiaasbaeaabiqaaaqaaiabbMgaPjabbohaZjabboeadjabb+gaVjabb2gaTjabbkgaIjabb+gaVjabcIcaOiabdAha2jabcYcaSiabbwha1jabbchaWjabbchaWjabbwgaLjabbkhaYjabbEeahjabbkhaYjabbMgaPjabbsgaKjabcIcaOiabbcfaqjabcIcaOiabdAha2jabcYcaSiabigdaXiabcMcaPiabcMcaPiabcMcaPiabg2da9Gqabiab=rha0jab=jhaYjab=vha1jab=vgaLjabcAcaMaqaaiabbMgaPjabbohaZjabboeadjabb+gaVjabb2gaTjabbkgaIjabb+gaVjabcIcaOiabdAha2jabcYcaSiabbYgaSjabb+gaVjabbEha3jabbwgaLjabbkhaYjabbEeahjabbkhaYjabbMgaPjabbsgaKjabcIcaOiabbcfaqjabcIcaOiabdAha2jabcYcaSiabigdaXiabcMcaPiabcMcaPiabcMcaPiabg2da9iab=rha0jab=jhaYjab=vha1jab=vgaLbaaaeaacqaIWaamaeaacqqGVbWBcqqG0baDcqqGObaAcqqGLbqzcqqGYbGCcqqG3bWDcqqGPbqAcqqGZbWCcqqGLbqzcqGGUaGlaaGaeiOla4IaaCzcaiaaxMaadaqadaqaaiabisda0aGaayjkaiaawMcaaaGaay5Eaaaaaa@A633@

We introduced *Adj*_*v *_(*L*) due to the following reason. First, suppose that three nodes with the same attribute are aligned vertically. We call them *v*_*α*_, *v*_*β*_, and *v*_*γ *_beginning from the bottom. There are three combo relations among the three nodes: one is between *v*_*α *_and *v*_*β*_, another between *v*_*β *_and *v*_*γ*_, and the third between *v*_*α *_and *v*_*γ*_. Although *v*_*β *_is involved in these three combo relations, the combo relation between *v*_*α *_and *v*_*γ *_is not considered in Combovβ
 MathType@MTEF@5@5@+=feaafiart1ev1aaatCvAUfKttLearuWrP9MDH5MBPbIqV92AaeXatLxBI9gBaebbnrfifHhDYfgasaacH8akY=wiFfYdH8Gipec8Eeeu0xXdbba9frFj0=OqFfea0dXdd9vqai=hGuQ8kuc9pgc9s8qqaq=dirpe0xb9q8qiLsFr0=vr0=vr0dc8meaabaqaciaacaGaaeqabaqabeGadaaakeaacqWGdbWqcqWGVbWBcqWGTbqBcqWGIbGycqWGVbWBdaWgaaWcbaGaemODay3aaSbaaWqaaGGaciab=j7aIbqabaaaleqaaaaa@36BA@ (*L*). Therefore, *Adj*_*v *_(*L*) is needed to correct this type of undercount.

### SCCB-grid layout algorithm: extension of the search space due to the swap operation

**Figure 4:**
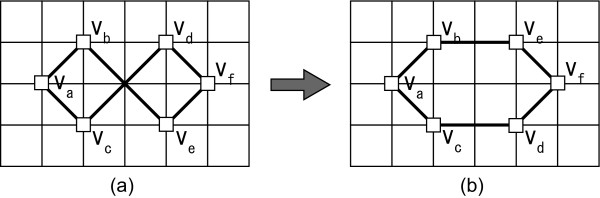
**An optimal layout of CB-grid and improved layout with the swap operation**. (a) An optimal layout for CB-grid layout algorithm. (b) From (a) a better layout will be generated with the swap operation.

Another drawback of CB-grid layout algorithm is that only one node can be moved to a vacant point at each step. For example, the layout shown in Figure 4(a) is optimal for CB-grid layout algorithm despite the fact the layout in Figure 4(b) should be selected as the better layout. This limitation is due to the strategy of CB-grid layout algorithm. Thus, we have devised a new algorithm by allowing the swap operations between two nodes while keeping the time complexity. With this improvement, the layout in Figure 4(a) will be arranged as shown in Figure 4(b). The new algorithm is named CCB-grid layout with the swap operation (SCCB-grid layout algorithm). The layout cost function is the same as in CCB-grid layout algorithm. However, a naïve implementation would increase the time complexity to calculate the layout cost for swapped layouts.

In the previous approach [15], Δ matrix stores cost differences that are induced only by moving nodes to vacant points. As a result, if a grid point of interest was occupied at the previous step, we cannot exploit Δ matrix to calculate cost differences corresponding to that grid point. Since grid points of interest on the swap operation are obviously occupied at the previous step, Δ matrix cannot be used. However, if Δ matrix also stores cost differences related to occupied points, Δ matrix can be exploited for this problematic case, too. We then propose an extended Δ matrix, which considers occupied points as well as vacant points. Since the definition of the cost differences for vacant points cannot be applied directly to occupied points, we decide to calculate the cost differences for the occupied points by calculating it without taking into account the node occupying that grid point and all edges connected to it. In the remainder of this section, we will show how to calculate the extended Δ matrix and then compare the time complexity of the extended Δ matrix and the original Δ matrix.

Henceforth, let us refer to the extended Δ matrix as Δ matrix. Given a layout *L*, at the first step, we update Δ (*L*) matrix as follows:

Δvαp(L)={Fvα(Tvα→pL)−Fvα(L)if p∈WLFvα(Tvα→pDvγL)−Fvα(DvγL)if p=P(vγ).     (5)
 MathType@MTEF@5@5@+=feaafiart1ev1aaatCvAUfKttLearuWrP9MDH5MBPbIqV92AaeXatLxBI9gBaebbnrfifHhDYfgasaacH8akY=wiFfYdH8Gipec8Eeeu0xXdbba9frFj0=OqFfea0dXdd9vqai=hGuQ8kuc9pgc9s8qqaq=dirpe0xb9q8qiLsFr0=vr0=vr0dc8meaabaqaciaacaGaaeqabaqabeGadaaakeaacqqHuoardaWgaaWcbaGaemODay3aaSbaaWqaaGGaciab=f7aHbqabaWccqWGWbaCaeqaaOGaeiikaGIaemitaWKaeiykaKIaeyypa0ZaaiqabeaafaqaaeGacaaabaGaemOray0aaSbaaSqaaiabdAha2naaBaaameaacqWFXoqyaeqaaaWcbeaakiabcIcaOiabdsfaunaaBaaaleaacqWG2bGDdaWgaaadbaGae8xSdegabeaaliabgkziUkabdchaWbqabaGccqWGmbatcqGGPaqkcqGHsislcqWGgbGrdaWgaaWcbaGaemODay3aaSbaaWqaaiab=f7aHbqabaaaleqaaOGaeiikaGIaemitaWKaeiykaKcabaacbaGae4xAaKMae4NzayMaeeiiaaIaemiCaaNaeyicI4Saem4vaC1aaSbaaSqaaiabdYeambqabaaakeaacqWGgbGrdaWgaaWcbaGaemODay3aaSbaaWqaaiab=f7aHbqabaaaleqaaOGaeiikaGIaemivaq1aaSbaaSqaaiabdAha2naaBaaameaacqWFXoqyaeqaaSGaeyOKH4QaemiCaahabeaakiabdseaenaaBaaaleaacqWG2bGDdaWgaaadbaGae83SdCgabeaaaSqabaGccqWGmbatcqGGPaqkcqGHsislcqWGgbGrdaWgaaWcbaGaemODay3aaSbaaWqaaiab=f7aHbqabaaaleqaaOGaeiikaGIaemiraq0aaSbaaSqaaiabdAha2naaBaaameaacqWFZoWzaeqaaaWcbeaakiabdYeamjabcMcaPaqaaiab+LgaPjab+zgaMjabbccaGiabdchaWjabg2da9iabdcfaqjabcIcaOiabdAha2naaBaaaleaacqWFZoWzaeqaaOGaeiykaKIaeiOla4caaaGaay5EaaGaaCzcaiaaxMaadaqadaqaaiabiwda1aGaayjkaiaawMcaaaaa@8A47@

Fvα
 MathType@MTEF@5@5@+=feaafiart1ev1aaatCvAUfKttLearuWrP9MDH5MBPbIqV92AaeXatLxBI9gBaebbnrfifHhDYfgasaacH8akY=wiFfYdH8Gipec8Eeeu0xXdbba9frFj0=OqFfea0dXdd9vqai=hGuQ8kuc9pgc9s8qqaq=dirpe0xb9q8qiLsFr0=vr0=vr0dc8meaabaqaciaacaGaaeqabaqabeGadaaakeaacqWGgbGrdaWgaaWcbaGaemODay3aaSbaaWqaaGGaciab=f7aHbqabaaaleqaaaaa@3140@ is the following function:

Fvα(L)=Wee∑ei∈Evα,ej∈ECrossei,ej(L)+Wne∑ek∈ECrossvα,ek(L)+Wdc∑vl∈VDistancevα,vl(L).     (6)
 MathType@MTEF@5@5@+=feaafiart1ev1aaatCvAUfKttLearuWrP9MDH5MBPbIqV92AaeXatLxBI9gBaebbnrfifHhDYfgasaacH8akY=wiFfYdH8Gipec8Eeeu0xXdbba9frFj0=OqFfea0dXdd9vqai=hGuQ8kuc9pgc9s8qqaq=dirpe0xb9q8qiLsFr0=vr0=vr0dc8meaabaqaciaacaGaaeqabaqabeGadaaakeaacqWGgbGrdaWgaaWcbaGaemODay3aaSbaaWqaaGGaciab=f7aHbqabaaaleqaaOGaeiikaGIaemitaWKaeiykaKIaeyypa0Jaem4vaC1aaSbaaSqaaiabdwgaLjabdwgaLbqabaGcdaaeqbqaaiabdoeadjabdkhaYjabd+gaVjabdohaZjabdohaZnaaBaaaleaacqWGLbqzdaWgaaadbaGaemyAaKgabeaaliabcYcaSiabdwgaLnaaBaaameaacqWGQbGAaeqaaaWcbeaaaeaacqWGLbqzdaWgaaadbaGaemyAaKgabeaaliabgIGiolabdweafnaaBaaameaacqWG2bGDdaWgaaqaaiab=f7aHbqabaaabeaaliabcYcaSiabdwgaLnaaBaaameaacqWGQbGAaeqaaSGaeyicI4SaemyraueabeqdcqGHris5aOGaeiikaGIaemitaWKaeiykaKIaey4kaSIaem4vaC1aaSbaaSqaaiabd6gaUjabdwgaLbqabaGcdaaeqbqaaiabdoeadjabdkhaYjabd+gaVjabdohaZjabdohaZnaaBaaaleaacqWG2bGDdaWgaaadbaGae8xSdegabeaaliabcYcaSiabdwgaLnaaBaaameaacqWGRbWAaeqaaaWcbeaaaeaacqWGLbqzdaWgaaadbaGaem4AaSgabeaaliabgIGiolabdweafbqab0GaeyyeIuoakiabcIcaOiabdYeamjabcMcaPiabgUcaRiabdEfaxnaaBaaaleaacqWGKbazcqWGJbWyaeqaaOWaaabuaeaaieGacqGFebarcqGFPbqAcqGFZbWCcqGF0baDcqGFHbqycqGFUbGBcqGFJbWycqGFLbqzdaWgaaWcbaGaemODay3aaSbaaWqaaiab=f7aHbqabaWccqGGSaalcqWG2bGDdaWgaaadbaGaemiBaWgabeaaaSqabaaabaGaemODay3aaSbaaWqaaiabdYgaSbqabaWccqGHiiIZcqWGwbGvaeqaniabggHiLdGccqGGOaakcqWGmbatcqGGPaqkcqGGUaGlcaWLjaGaaCzcamaabmaabaGaeGOnaydacaGLOaGaayzkaaaaaa@9EBD@

If the previous layout is updated by moving node *v*_*β *_to vacant point *q*, Δ (Tvα→p
 MathType@MTEF@5@5@+=feaafiart1ev1aaatCvAUfKttLearuWrP9MDH5MBPbIqV92AaeXatLxBI9gBaebbnrfifHhDYfgasaacH8akY=wiFfYdH8Gipec8Eeeu0xXdbba9frFj0=OqFfea0dXdd9vqai=hGuQ8kuc9pgc9s8qqaq=dirpe0xb9q8qiLsFr0=vr0=vr0dc8meaabaqaciaacaGaaeqabaqabeGadaaakeaacqWGubavdaWgaaWcbaGaemODay3aaSbaaWqaaGGaciab=f7aHbqabaWccqGHsgIRcqWGWbaCaeqaaaaa@34B2@*L*) can be updated efficiently by using Δ (*L*) as follows:

Δvαp(Tvβ→qL)={Δvβp(L)−Δvβq(L),if vα=vβ,p∈WTvβ→qL(case 1)Δvαp(L)+DIFF0,if vα≠vβ,p∈WTvβ→qL\P(vβ)(case 2)Δvαp(L)+DIFF1,if vα≠vβ,p=P(vβ)(case 3)Δvβp(L)−Δvβq(L)+DIFF2,if vα=vβ,p=P(vγ)(case 4)Δvαp(L)+DIFF3if vα≠vβ,p=P(vγ)(case 5)Δvαp(L)+DIFF4,if vα≠vβ,p=q(case 6),     (7)
 MathType@MTEF@5@5@+=feaafiart1ev1aaatCvAUfKttLearuWrP9MDH5MBPbIqV92AaeXatLxBI9gBaebbnrfifHhDYfgasaacH8akY=wiFfYdH8Gipec8Eeeu0xXdbba9frFj0=OqFfea0dXdd9vqai=hGuQ8kuc9pgc9s8qqaq=dirpe0xb9q8qiLsFr0=vr0=vr0dc8meaabaqaciaacaGaaeqabaqabeGadaaakeaacqqHuoardaWgaaWcbaGaemODay3aaSbaaWqaaGGaciab=f7aHbqabaWccqWGWbaCaeqaaOGaeiikaGIaemivaq1aaSbaaSqaaiabdAha2naaBaaameaacqWFYoGyaeqaaSGaeyOKH4QaemyCaehabeaakiabdYeamjabcMcaPiabg2da9maaceqabaqbaeaabyWaaaaabaGaeuiLdq0aaSbaaSqaaiabdAha2naaBaaameaacqWFYoGyaeqaaSGaemiCaahabeaakiabcIcaOiabdYeamjabcMcaPiabgkHiTiabfs5aenaaBaaaleaacqWG2bGDdaWgaaadbaGae8NSdigabeaaliabdghaXbqabaGccqGGOaakcqWGmbatcqGGPaqkcqGGSaalaeaacqqGPbqAcqqGMbGzcqqGGaaicqWG2bGDdaWgaaWcbaGae8xSdegabeaakiabg2da9iabdAha2naaBaaaleaacqWFYoGyaeqaaOGaeiilaWIaemiCaaNaeyicI4Saem4vaC1aaSbaaSqaaiabdsfaunaaBaaameaacqWG2bGDdaWgaaqaaiab=j7aIbqabaGaeyOKH4QaemyCaehabeaaliabdYeambqabaaakeaadaqadaqaaiabbogaJjabbggaHjabbohaZjabbwgaLjabbccaGiabigdaXaGaayjkaiaawMcaaaqaaiabfs5aenaaBaaaleaacqWG2bGDdaWgaaadbaGae8xSdegabeaaliabdchaWbqabaGccqGGOaakcqWGmbatcqGGPaqkcqGHRaWkcqWGebarcqWGjbqscqWGgbGrcqWGgbGrdaWgaaWcbaGaeGimaadabeaakiabcYcaSaqaaiabbMgaPjabbAgaMjabbccaGiabdAha2naaBaaaleaacqWFXoqyaeqaaOGaeyiyIKRaemODay3aaSbaaSqaaiab=j7aIbqabaGccqGGSaalcqWGWbaCcqGHiiIZcqWGxbWvdaWgaaWcbaGaemivaq1aaSbaaWqaaiabdAha2naaBaaabaGae8NSdigabeaacqGHsgIRcqWGXbqCaeqaaSGaemitaWeabeaakiabcYfaCjabdcfaqjabcIcaOiabdAha2naaBaaaleaacqWFYoGyaeqaaOGaeiykaKcabaWaaeWaaeaacqqGJbWycqqGHbqycqqGZbWCcqqGLbqzcqqGGaaicqaIYaGmaiaawIcacaGLPaaaaeaacqqHuoardaWgaaWcbaGaemODay3aaSbaaWqaaiab=f7aHbqabaWccqWGWbaCaeqaaOGaeiikaGIaemitaWKaeiykaKIaey4kaSIaemiraqKaemysaKKaemOrayKaemOray0aaSbaaSqaaiabigdaXaqabaGccqGGSaalaeaacqqGPbqAcqqGMbGzcqqGGaaicqWG2bGDdaWgaaWcbaGae8xSdegabeaakiabgcMi5kabdAha2naaBaaaleaacqWFYoGyaeqaaOGaeiilaWIaemiCaaNaeyypa0JaemiuaaLaeiikaGIaemODay3aaSbaaSqaaiab=j7aIbqabaGccqGGPaqkaeaadaqadaqaaiabbogaJjabbggaHjabbohaZjabbwgaLjabbccaGiabiodaZaGaayjkaiaawMcaaaqaaiabfs5aenaaBaaaleaacqWG2bGDdaWgaaadbaGae8NSdigabeaaliabdchaWbqabaGccqGGOaakcqWGmbatcqGGPaqkcqGHsislcqqHuoardaWgaaWcbaGaemODay3aaSbaaWqaaiab=j7aIbqabaWccqWGXbqCaeqaaOGaeiikaGIaemitaWKaeiykaKIaey4kaSIaemiraqKaemysaKKaemOrayKaemOray0aaSbaaSqaaiabikdaYaqabaGccqGGSaalaeaacqqGPbqAcqqGMbGzcqqGGaaicqWG2bGDdaWgaaWcbaGae8xSdegabeaakiabg2da9iabdAha2naaBaaaleaacqWFYoGyaeqaaOGaeiilaWIaemiCaaNaeyypa0JaemiuaaLaeiikaGIaemODay3aaSbaaSqaaiab=n7aNbqabaGccqGGPaqkaeaadaqadaqaaiabbogaJjabbggaHjabbohaZjabbwgaLjabbccaGiabisda0aGaayjkaiaawMcaaaqaaiabfs5aenaaBaaaleaacqWG2bGDdaWgaaadbaGae8xSdegabeaaliabdchaWbqabaGccqGGOaakcqWGmbatcqGGPaqkcqGHRaWkcqWGebarcqWGjbqscqWGgbGrcqWGgbGrdaWgaaWcbaGaeG4mamdabeaaaOqaaiabbMgaPjabbAgaMjabbccaGiabdAha2naaBaaaleaacqWFXoqyaeqaaOGaeyiyIKRaemODay3aaSbaaSqaaiab=j7aIbqabaGccqGGSaalcqWGWbaCcqGH9aqpcqWGqbaucqGGOaakcqWG2bGDdaWgaaWcbaGae83SdCgabeaakiabcMcaPaqaamaabmaabaGaee4yamMaeeyyaeMaee4CamNaeeyzauMaeeiiaaIaeGynaudacaGLOaGaayzkaaaabaGaeuiLdq0aaSbaaSqaaiabdAha2naaBaaameaacqWFXoqyaeqaaSGaemiCaahabeaakiabcIcaOiabdYeamjabcMcaPiabgUcaRiabdseaejabdMeajjabdAeagjabdAeagnaaBaaaleaacqaI0aanaeqaaOGaeiilaWcabaGaeeyAaKMaeeOzayMaeeiiaaIaemODay3aaSbaaSqaaiab=f7aHbqabaGccqGHGjsUcqWG2bGDdaWgaaWcbaGae8NSdigabeaakiabcYcaSiabdchaWjabg2da9iabdghaXbqaamaabmaabaGaee4yamMaeeyyaeMaee4CamNaeeyzauMaeeiiaaIaeGOnaydacaGLOaGaayzkaaGaeiilaWcaaaGaay5EaaGaaCzcaiaaxMaadaqadaqaaiabiEda3aGaayjkaiaawMcaaaaa@71B8@

where *DIFF*_0 _to *DIFF*_4 _are defined in the following way:

DIFF0=Qvα,vβ(Tvα→pTvβ→qL)−Qvα,vβ(Tvβ→qL)−Qvα,vβ(Tvα→pL)+Qvα,vβ(L)     (8)
 MathType@MTEF@5@5@+=feaafiart1ev1aaatCvAUfKttLearuWrP9MDH5MBPbIqV92AaeXatLxBI9gBaebbnrfifHhDYfgasaacH8akY=wiFfYdH8Gipec8Eeeu0xXdbba9frFj0=OqFfea0dXdd9vqai=hGuQ8kuc9pgc9s8qqaq=dirpe0xb9q8qiLsFr0=vr0=vr0dc8meaabaqaciaacaGaaeqabaqabeGadaaakeaacqWGebarcqWGjbqscqWGgbGrcqWGgbGrdaWgaaWcbaGaeGimaadabeaakiabg2da9iabdgfarnaaBaaaleaacqWG2bGDdaWgaaadbaacciGae8xSdegabeaaliabcYcaSiabdAha2naaBaaameaacqWFYoGyaeqaaaWcbeaakiabcIcaOiabdsfaunaaBaaaleaacqWG2bGDdaWgaaadbaGae8xSdegabeaaliabgkziUkabdchaWbqabaGccqWGubavdaWgaaWcbaGaemODay3aaSbaaWqaaiab=j7aIbqabaWccqGHsgIRcqWGXbqCaeqaaOGaemitaWKaeiykaKIaeyOeI0Iaemyuae1aaSbaaSqaaiabdAha2naaBaaameaacqWFXoqyaeqaaSGaeiilaWIaemODay3aaSbaaWqaaiab=j7aIbqabaaaleqaaOGaeiikaGIaemivaq1aaSbaaSqaaiabdAha2naaBaaameaacqWFYoGyaeqaaSGaeyOKH4QaemyCaehabeaakiabdYeamjabcMcaPiabgkHiTiabdgfarnaaBaaaleaacqWG2bGDdaWgaaadbaGae8xSdegabeaaliabcYcaSiabdAha2naaBaaameaacqWFYoGyaeqaaaWcbeaakiabcIcaOiabdsfaunaaBaaaleaacqWG2bGDdaWgaaadbaGae8xSdegabeaaliabgkziUkabdchaWbqabaGccqWGmbatcqGGPaqkcqGHRaWkcqWGrbqudaWgaaWcbaGaemODay3aaSbaaWqaaiab=f7aHbqabaWccqGGSaalcqWG2bGDdaWgaaadbaGae8NSdigabeaaaSqabaGccqGGOaakcqWGmbatcqGGPaqkcaWLjaGaaCzcamaabmaabaGaeGioaGdacaGLOaGaayzkaaaaaa@8867@

DIFF1=Qvα,vβ(Tvα→pTvβ→qL)−Qvα,vβ(Tvβ→qL)     (9)
 MathType@MTEF@5@5@+=feaafiart1ev1aaatCvAUfKttLearuWrP9MDH5MBPbIqV92AaeXatLxBI9gBaebbnrfifHhDYfgasaacH8akY=wiFfYdH8Gipec8Eeeu0xXdbba9frFj0=OqFfea0dXdd9vqai=hGuQ8kuc9pgc9s8qqaq=dirpe0xb9q8qiLsFr0=vr0=vr0dc8meaabaqaciaacaGaaeqabaqabeGadaaakeaacqWGebarcqWGjbqscqWGgbGrcqWGgbGrdaWgaaWcbaGaeGymaedabeaakiabg2da9iabdgfarnaaBaaaleaacqWG2bGDdaWgaaadbaacciGae8xSdegabeaaliabcYcaSiabdAha2naaBaaameaacqWFYoGyaeqaaaWcbeaakiabcIcaOiabdsfaunaaBaaaleaacqWG2bGDdaWgaaadbaGae8xSdegabeaaliabgkziUkabdchaWbqabaGccqWGubavdaWgaaWcbaGaemODay3aaSbaaWqaaiab=j7aIbqabaWccqGHsgIRcqWGXbqCaeqaaOGaemitaWKaeiykaKIaeyOeI0Iaemyuae1aaSbaaSqaaiabdAha2naaBaaameaacqWFXoqyaeqaaSGaeiilaWIaemODay3aaSbaaWqaaiab=j7aIbqabaaaleqaaOGaeiikaGIaemivaq1aaSbaaSqaaiabdAha2naaBaaameaacqWFYoGyaeqaaSGaeyOKH4QaemyCaehabeaakiabdYeamjabcMcaPiaaxMaacaWLjaWaaeWaaeaacqaI5aqoaiaawIcacaGLPaaaaaa@6750@

DIFF2=Qvβ,vγ(Tvβ→qL)−Qvβ,vγ(L)     (10)
 MathType@MTEF@5@5@+=feaafiart1ev1aaatCvAUfKttLearuWrP9MDH5MBPbIqV92AaeXatLxBI9gBaebbnrfifHhDYfgasaacH8akY=wiFfYdH8Gipec8Eeeu0xXdbba9frFj0=OqFfea0dXdd9vqai=hGuQ8kuc9pgc9s8qqaq=dirpe0xb9q8qiLsFr0=vr0=vr0dc8meaabaqaciaacaGaaeqabaqabeGadaaakeaacqWGebarcqWGjbqscqWGgbGrcqWGgbGrdaWgaaWcbaGaeGOmaidabeaakiabg2da9iabdgfarnaaBaaaleaacqWG2bGDdaWgaaadbaacciGae8NSdigabeaaliabcYcaSiabdAha2naaBaaameaacqWFZoWzaeqaaaWcbeaakiabcIcaOiabdsfaunaaBaaaleaacqWG2bGDdaWgaaadbaGae8NSdigabeaaliabgkziUkabdghaXbqabaGccqWGmbatcqGGPaqkcqGHsislcqWGrbqudaWgaaWcbaGaemODay3aaSbaaWqaaiab=j7aIbqabaWccqGGSaalcqWG2bGDdaWgaaadbaGae83SdCgabeaaaSqabaGccqGGOaakcqWGmbatcqGGPaqkcaWLjaGaaCzcamaabmaabaGaeGymaeJaeGimaadacaGLOaGaayzkaaaaaa@5834@

DIFF3=Qvα,vβ(Tvα→pTvβ→qDvγL)−Qvα,vβ(Tvβ→qDvγL)−Qvα,vβ(Tvα→pDvγL)+Qvα,vβ(DvγL)     (11)
 MathType@MTEF@5@5@+=feaafiart1ev1aaatCvAUfKttLearuWrP9MDH5MBPbIqV92AaeXatLxBI9gBaebbnrfifHhDYfgasaacH8akY=wiFfYdH8Gipec8Eeeu0xXdbba9frFj0=OqFfea0dXdd9vqai=hGuQ8kuc9pgc9s8qqaq=dirpe0xb9q8qiLsFr0=vr0=vr0dc8meaabaqaciaacaGaaeqabaqabeGadaaakeaacqWGebarcqWGjbqscqWGgbGrcqWGgbGrdaWgaaWcbaGaeG4mamdabeaakiabg2da9iabdgfarnaaBaaaleaacqWG2bGDdaWgaaadbaacciGae8xSdegabeaaliabcYcaSiabdAha2naaBaaameaacqWFYoGyaeqaaaWcbeaakiabcIcaOiabdsfaunaaBaaaleaacqWG2bGDdaWgaaadbaGae8xSdegabeaaliabgkziUkabdchaWbqabaGccqWGubavdaWgaaWcbaGaemODay3aaSbaaWqaaiab=j7aIbqabaWccqGHsgIRcqWGXbqCaeqaaOGaemiraq0aaSbaaSqaaiabdAha2naaBaaameaacqWFZoWzaeqaaaWcbeaakiabdYeamjabcMcaPiabgkHiTiabdgfarnaaBaaaleaacqWG2bGDdaWgaaadbaGae8xSdegabeaaliabcYcaSiabdAha2naaBaaameaacqWFYoGyaeqaaaWcbeaakiabcIcaOiabdsfaunaaBaaaleaacqWG2bGDdaWgaaadbaGae8NSdigabeaaliabgkziUkabdghaXbqabaGccqWGebardaWgaaWcbaGaemODay3aaSbaaWqaaiab=n7aNbqabaaaleqaaOGaemitaWKaeiykaKIaeyOeI0Iaemyuae1aaSbaaSqaaiabdAha2naaBaaameaacqWFXoqyaeqaaSGaeiilaWIaemODay3aaSbaaWqaaiab=j7aIbqabaaaleqaaOGaeiikaGIaemivaq1aaSbaaSqaaiabdAha2naaBaaameaacqWFXoqyaeqaaSGaeyOKH4QaemiCaahabeaakiabdseaenaaBaaaleaacqWG2bGDdaWgaaadbaGae83SdCgabeaaaSqabaGccqWGmbatcqGGPaqkcqGHRaWkcqWGrbqudaWgaaWcbaGaemODay3aaSbaaWqaaiab=f7aHbqabaWccqGGSaalcqWG2bGDdaWgaaadbaGae8NSdigabeaaaSqabaGccqGGOaakcqWGebardaWgaaWcbaGaemODay3aaSbaaWqaaiab=n7aNbqabaaaleqaaOGaemitaWKaeiykaKIaaCzcaiaaxMaadaqadaqaaiabigdaXiabigdaXaGaayjkaiaawMcaaaaa@9BA7@

DIFF4=−Qvα,vβ(Tvα→pL)+Qvα,vβ(L),     (12)
 MathType@MTEF@5@5@+=feaafiart1ev1aaatCvAUfKttLearuWrP9MDH5MBPbIqV92AaeXatLxBI9gBaebbnrfifHhDYfgasaacH8akY=wiFfYdH8Gipec8Eeeu0xXdbba9frFj0=OqFfea0dXdd9vqai=hGuQ8kuc9pgc9s8qqaq=dirpe0xb9q8qiLsFr0=vr0=vr0dc8meaabaqaciaacaGaaeqabaqabeGadaaakeaacqWGebarcqWGjbqscqWGgbGrcqWGgbGrdaWgaaWcbaGaeGinaqdabeaakiabg2da9iabgkHiTiabdgfarnaaBaaaleaacqWG2bGDdaWgaaadbaacciGae8xSdegabeaaliabcYcaSiabdAha2naaBaaameaacqWFYoGyaeqaaaWcbeaakiabcIcaOiabdsfaunaaBaaaleaacqWG2bGDdaWgaaadbaGae8xSdegabeaaliabgkziUkabdchaWbqabaGccqWGmbatcqGGPaqkcqGHRaWkcqWGrbqudaWgaaWcbaGaemODay3aaSbaaWqaaiab=f7aHbqabaWccqGGSaalcqWG2bGDdaWgaaadbaGae8NSdigabeaaaSqabaGccqGGOaakcqWGmbatcqGGPaqkcqGGSaalcaWLjaGaaCzcamaabmaabaGaeGymaeJaeGOmaidacaGLOaGaayzkaaaaaa@59EA@

where *Q *shall be defined below.

If the previous layout is updated by swapping two nodes vβ1
 MathType@MTEF@5@5@+=feaafiart1ev1aaatCvAUfKttLearuWrP9MDH5MBPbIqV92AaeXatLxBI9gBaebbnrfifHhDYfgasaacH8akY=wiFfYdH8Gipec8Eeeu0xXdbba9frFj0=OqFfea0dXdd9vqai=hGuQ8kuc9pgc9s8qqaq=dirpe0xb9q8qiLsFr0=vr0=vr0dc8meaabaqaciaacaGaaeqabaqabeGadaaakeaacqWG2bGDdaWgaaWcbaacciGae8NSdi2aaSbaaWqaaiabigdaXaqabaaaleqaaaaa@311D@ and vβ2
 MathType@MTEF@5@5@+=feaafiart1ev1aaatCvAUfKttLearuWrP9MDH5MBPbIqV92AaeXatLxBI9gBaebbnrfifHhDYfgasaacH8akY=wiFfYdH8Gipec8Eeeu0xXdbba9frFj0=OqFfea0dXdd9vqai=hGuQ8kuc9pgc9s8qqaq=dirpe0xb9q8qiLsFr0=vr0=vr0dc8meaabaqaciaacaGaaeqabaqabeGadaaakeaacqWG2bGDdaWgaaWcbaacciGae8NSdi2aaSbaaWqaaiabikdaYaqabaaaleqaaaaa@311F@, Δ (Svβ1↔vβ2
 MathType@MTEF@5@5@+=feaafiart1ev1aaatCvAUfKttLearuWrP9MDH5MBPbIqV92AaeXatLxBI9gBaebbnrfifHhDYfgasaacH8akY=wiFfYdH8Gipec8Eeeu0xXdbba9frFj0=OqFfea0dXdd9vqai=hGuQ8kuc9pgc9s8qqaq=dirpe0xb9q8qiLsFr0=vr0=vr0dc8meaabaqaciaacaGaaeqabaqabeGadaaakeaacqWGtbWudaWgaaWcbaGaemODay3aaSbaaWqaaGGaciab=j7aInaaBaaabaGaeGymaedabeaaaeqaaSGaeyiLHSQaemODay3aaSbaaWqaaiab=j7aInaaBaaabaGaeGOmaidabeaaaeqaaaWcbeaaaaa@38B5@*L*) is then updated efficiently by using Δ (*L*) as follows:

Δvαp(Svβ1↔vβ2L)={Δvβ1p(L)−Δvβ1P(vβ2)(L)+DIFF5,if vα=vβ1,p∈WL(case 1)Δvαp(L)+DIFF6,if vα≠vβ1,p∈WL\{P(vβ1),P(vβ2)}(case 2)Δvβ1p(L)−Δvβ1P(vβ2)(L)+DIFF7,if vα=vβ1,p=P(vγ)(case 3)Δvαp(L)+DIFF8,if vα≠vβ1,vα≠vβ2,p=P(vγ)(case 4)Δvαp(L)+DIFF9,if vα≠vβ1,vα≠vβ2,p=P(vβ2)(case 5),     (13)
 MathType@MTEF@5@5@+=feaafiart1ev1aaatCvAUfKttLearuWrP9MDH5MBPbIqV92AaeXatLxBI9gBaebbnrfifHhDYfgasaacH8akY=wiFfYdH8Gipec8Eeeu0xXdbba9frFj0=OqFfea0dXdd9vqai=hGuQ8kuc9pgc9s8qqaq=dirpe0xb9q8qiLsFr0=vr0=vr0dc8meaabaqaciaacaGaaeqabaqabeGadaaakeaacqqHuoardaWgaaWcbaGaemODay3aaSbaaWqaaGGaciab=f7aHbqabaWccqWGWbaCaeqaaOGaeiikaGIaem4uam1aaSbaaSqaaiabdAha2naaBaaameaacqWFYoGydaWgaaqaaiabigdaXaqabaaabeaaliabgsziRkabdAha2naaBaaameaacqWFYoGydaWgaaqaaiabikdaYaqabaaabeaaaSqabaGccqWGmbatcqGGPaqkcqGH9aqpdaGabeqaauaabaqafmaaaaqaaiabfs5aenaaBaaaleaacqWG2bGDdaWgaaadbaGae8NSdi2aaSbaaeaacqaIXaqmaeqaaaqabaWccqWGWbaCaeqaaOGaeiikaGIaemitaWKaeiykaKIaeyOeI0IaeuiLdq0aaSbaaSqaaiabdAha2naaBaaameaacqWFYoGydaWgaaqaaiabigdaXaqabaaabeaaliabdcfaqjabcIcaOiabdAha2naaBaaameaacqWFYoGydaWgaaqaaiabikdaYaqabaaabeaaliabcMcaPaqabaGccqGGOaakcqWGmbatcqGGPaqkcqGHRaWkcqWGebarcqWGjbqscqWGgbGrcqWGgbGrdaWgaaWcbaGaeGynaudabeaakiabcYcaSaqaaiabbMgaPjabbAgaMjabbccaGiabdAha2naaBaaaleaacqWFXoqyaeqaaOGaeyypa0JaemODay3aaSbaaSqaaiab=j7aInaaBaaameaacqaIXaqmaeqaaaWcbeaakiabcYcaSiabdchaWjabgIGiolabdEfaxnaaBaaaleaacqWGmbataeqaaaGcbaWaaeWaaeaacqqGJbWycqqGHbqycqqGZbWCcqqGLbqzcqqGGaaicqaIXaqmaiaawIcacaGLPaaaaeaacqqHuoardaWgaaWcbaGaemODay3aaSbaaWqaaiab=f7aHbqabaWccqWGWbaCaeqaaOGaeiikaGIaemitaWKaeiykaKIaey4kaSIaemiraqKaemysaKKaemOrayKaemOray0aaSbaaSqaaiabiAda2aqabaGccqGGSaalaeaacqqGPbqAcqqGMbGzcqqGGaaicqWG2bGDdaWgaaWcbaGae8xSdegabeaakiabgcMi5kabdAha2naaBaaaleaacqWFYoGydaWgaaadbaGaeGymaedabeaaaSqabaGccqGGSaalcqWGWbaCcqGHiiIZcqWGxbWvdaWgaaWcbaGaemitaWeabeaakiabcYfaCjabcUha7jabdcfaqjabcIcaOiabdAha2naaBaaaleaacqWFYoGydaWgaaadbaGaeGymaedabeaaaSqabaGccqGGPaqkcqGGSaalcqWGqbaucqGGOaakcqWG2bGDdaWgaaWcbaGae8NSdi2aaSbaaWqaaiabikdaYaqabaaaleqaaOGaeiykaKIaeiyFa0habaWaaeWaaeaacqqGJbWycqqGHbqycqqGZbWCcqqGLbqzcqqGGaaicqaIYaGmaiaawIcacaGLPaaaaeaacqqHuoardaWgaaWcbaGaemODay3aaSbaaWqaaiab=j7aInaaBaaabaGaeGymaedabeaaaeqaaSGaemiCaahabeaakiabcIcaOiabdYeamjabcMcaPiabgkHiTiabfs5aenaaBaaaleaacqWG2bGDdaWgaaadbaGae8NSdi2aaSbaaeaacqaIXaqmaeqaaaqabaWccqWGqbaucqGGOaakcqWG2bGDdaWgaaadbaGae8NSdi2aaSbaaeaacqaIYaGmaeqaaaqabaWccqGGPaqkaeqaaOGaeiikaGIaemitaWKaeiykaKIaey4kaSIaemiraqKaemysaKKaemOrayKaemOray0aaSbaaSqaaiabiEda3aqabaGccqGGSaalaeaacqqGPbqAcqqGMbGzcqqGGaaicqWG2bGDdaWgaaWcbaGae8xSdegabeaakiabg2da9iabdAha2naaBaaaleaacqWFYoGydaWgaaadbaGaeGymaedabeaaaSqabaGccqGGSaalcqWGWbaCcqGH9aqpcqWGqbaucqGGOaakcqWG2bGDdaWgaaWcbaGae83SdCgabeaakiabcMcaPaqaamaabmaabaGaee4yamMaeeyyaeMaee4CamNaeeyzauMaeeiiaaIaeG4mamdacaGLOaGaayzkaaaabaGaeuiLdq0aaSbaaSqaaiabdAha2naaBaaameaacqWFXoqyaeqaaSGaemiCaahabeaakiabcIcaOiabdYeamjabcMcaPiabgUcaRiabdseaejabdMeajjabdAeagjabdAeagnaaBaaaleaacqaI4aaoaeqaaOGaeiilaWcabaGaeeyAaKMaeeOzayMaeeiiaaIaemODay3aaSbaaSqaaiab=f7aHbqabaGccqGHGjsUcqWG2bGDdaWgaaWcbaGae8NSdi2aaSbaaWqaaiabigdaXaqabaaaleqaaOGaeiilaWIaemODay3aaSbaaSqaaiab=f7aHbqabaGccqGHGjsUcqWG2bGDdaWgaaWcbaGae8NSdi2aaSbaaWqaaiabikdaYaqabaaaleqaaOGaeiilaWIaemiCaaNaeyypa0JaemiuaaLaeiikaGIaemODay3aaSbaaSqaaiab=n7aNbqabaGccqGGPaqkaeaadaqadaqaaiabbogaJjabbggaHjabbohaZjabbwgaLjabbccaGiabisda0aGaayjkaiaawMcaaaqaaiabfs5aenaaBaaaleaacqWG2bGDdaWgaaadbaGae8xSdegabeaaliabdchaWbqabaGccqGGOaakcqWGmbatcqGGPaqkcqGHRaWkcqWGebarcqWGjbqscqWGgbGrcqWGgbGrdaWgaaWcbaGaeGyoaKdabeaakiabcYcaSaqaaiabbMgaPjabbAgaMjabbccaGiabdAha2naaBaaaleaacqWFXoqyaeqaaOGaeyiyIKRaemODay3aaSbaaSqaaiab=j7aInaaBaaameaacqaIXaqmaeqaaaWcbeaakiabcYcaSiabdAha2naaBaaaleaacqWFXoqyaeqaaOGaeyiyIKRaemODay3aaSbaaSqaaiab=j7aInaaBaaameaacqaIYaGmaeqaaaWcbeaakiabcYcaSiabdchaWjabg2da9iabdcfaqjabcIcaOiabdAha2naaBaaaleaacqWFYoGydaWgaaadbaacbiGae4NmaidabeaaaSqabaGccqGGPaqkaeaadaqadaqaaiabbogaJjabbggaHjabbohaZjabbwgaLjabbccaGiabbwda1aGaayjkaiaawMcaaiabcYcaSaaaaiaawUhaaiaaxMaacaWLjaWaaeWaaeaacqaIXaqmcqaIZaWmaiaawIcacaGLPaaaaaa@7C80@

where *DIFF*_5 _to *DIFF*_9 _are defined in the following way:

DIFF5=Qvβ1,vβ2(Tvβ1→pSvβ1↔vβ1L)−Qvβ1,vβ2(Svβ1↔vβ2L)−Qvβ1,vβ2(Tvβ1→pL)+Qvβ1,vβ2(L)     (14)
 MathType@MTEF@5@5@+=feaafiart1ev1aaatCvAUfKttLearuWrP9MDH5MBPbIqV92AaeXatLxBI9gBaebbnrfifHhDYfgasaacH8akY=wiFfYdH8Gipec8Eeeu0xXdbba9frFj0=OqFfea0dXdd9vqai=hGuQ8kuc9pgc9s8qqaq=dirpe0xb9q8qiLsFr0=vr0=vr0dc8meaabaqaciaacaGaaeqabaqabeGadaaakeaacqWGebarcqWGjbqscqWGgbGrcqWGgbGrdaWgaaWcbaGaeGynaudabeaakiabg2da9iabdgfarnaaBaaaleaacqWG2bGDdaWgaaadbaacciGae8NSdi2aaSbaaeaacqaIXaqmaeqaaaqabaWccqGGSaalcqWG2bGDdaWgaaadbaGae8NSdi2aaSbaaeaacqaIYaGmaeqaaaqabaaaleqaaOGaeiikaGIaemivaq1aaSbaaSqaaiabdAha2naaBaaameaacqWFYoGydaWgaaqaaiabigdaXaqabaaabeaaliabgkziUkabdchaWbqabaGccqWGtbWudaWgaaWcbaGaemODay3aaSbaaWqaaiab=j7aInaaBaaabaGaeGymaedabeaaaeqaaSGaeyiLHSQaemODay3aaSbaaWqaaiab=j7aInaaBaaabaGaeGymaedabeaaaeqaaaWcbeaakiabdYeamjabcMcaPiabgkHiTiabdgfarnaaBaaaleaacqWG2bGDdaWgaaadbaGae8NSdi2aaSbaaeaacqaIXaqmaeqaaaqabaWccqGGSaalcqWG2bGDdaWgaaadbaGae8NSdi2aaSbaaeaacqaIYaGmaeqaaaqabaaaleqaaOGaeiikaGIaem4uam1aaSbaaSqaaiabdAha2naaBaaameaacqWFYoGydaWgaaqaaiabigdaXaqabaaabeaaliabgsziRkabdAha2naaBaaameaacqWFYoGydaWgaaqaaiabikdaYaqabaaabeaaaSqabaGccqWGmbatcqGGPaqkcqGHsislcqWGrbqudaWgaaWcbaGaemODay3aaSbaaWqaaiab=j7aInaaBaaabaGaeGymaedabeaaaeqaaSGaeiilaWIaemODay3aaSbaaWqaaiab=j7aInaaBaaabaGaeGOmaidabeaaaeqaaaWcbeaakiabcIcaOiabdsfaunaaBaaaleaacqWG2bGDdaWgaaadbaGae8NSdi2aaSbaaeaacqaIXaqmaeqaaaqabaWccqGHsgIRcqWGWbaCaeqaaOGaemitaWKaeiykaKIaey4kaSIaemyuae1aaSbaaSqaaiabdAha2naaBaaameaacqWFYoGydaWgaaqaaiabigdaXaqabaaabeaaliabcYcaSiabdAha2naaBaaameaacqWFYoGydaWgaaqaaiabikdaYaqabaaabeaaaSqabaGccqGGOaakcqWGmbatcqGGPaqkcaWLjaGaaCzcamaabmaabaGaeGymaeJaeGinaqdacaGLOaGaayzkaaaaaa@9C13@

DIFF6=Q^vα,vβ1,vβ2(Tvα→pSvβ1↔vβ2L)−Q^vα,vβ1,vβ2(Svβ1↔vβ2L)−Q^vα,vβ1,vβ2(Tvα→pL)+Q^vα,vβ1,vβ2(L)     (15)
 MathType@MTEF@5@5@+=feaafiart1ev1aaatCvAUfKttLearuWrP9MDH5MBPbIqV92AaeXatLxBI9gBaebbnrfifHhDYfgasaacH8akY=wiFfYdH8Gipec8Eeeu0xXdbba9frFj0=OqFfea0dXdd9vqai=hGuQ8kuc9pgc9s8qqaq=dirpe0xb9q8qiLsFr0=vr0=vr0dc8meaabaqaciaacaGaaeqabaqabeGadaaakeaacqWGebarcqWGjbqscqWGgbGrcqWGgbGrdaWgaaWcbaGaeGOnaydabeaakiabg2da9iqbdgfarzaajaWaaSbaaSqaaiabdAha2naaBaaameaaiiGacqWFXoqyaeqaaSGaeiilaWIaemODay3aaSbaaWqaaiab=j7aInaaBaaabaGaeGymaedabeaaaeqaaSGaeiilaWIaemODay3aaSbaaWqaaiab=j7aInaaBaaabaGaeGOmaidabeaaaeqaaaWcbeaakiabcIcaOiabdsfaunaaBaaaleaacqWG2bGDdaWgaaadbaGae8xSdegabeaaliabgkziUkabdchaWbqabaGccqWGtbWudaWgaaWcbaGaemODay3aaSbaaWqaaiab=j7aInaaBaaabaGaeGymaedabeaaaeqaaSGaeyiLHSQaemODay3aaSbaaWqaaiab=j7aInaaBaaabaGaeGOmaidabeaaaeqaaaWcbeaakiabdYeamjabcMcaPiabgkHiTiqbdgfarzaajaWaaSbaaSqaaiabdAha2naaBaaameaacqWFXoqyaeqaaSGaeiilaWIaemODay3aaSbaaWqaaiab=j7aInaaBaaabaGaeGymaedabeaaaeqaaSGaeiilaWIaemODay3aaSbaaWqaaiab=j7aInaaBaaabaGaeGOmaidabeaaaeqaaaWcbeaakiabcIcaOiabdofatnaaBaaaleaacqWG2bGDdaWgaaadbaGae8NSdi2aaSbaaeaacqaIXaqmaeqaaaqabaWccqGHugYQcqWG2bGDdaWgaaadbaGae8NSdi2aaSbaaeaacqaIYaGmaeqaaaqabaaaleqaaOGaemitaWKaeiykaKIaeyOeI0IafmyuaeLbaKaadaWgaaWcbaGaemODay3aaSbaaWqaaiab=f7aHbqabaWccqGGSaalcqWG2bGDdaWgaaadbaGae8NSdi2aaSbaaeaacqaIXaqmaeqaaaqabaWccqGGSaalcqWG2bGDdaWgaaadbaGae8NSdi2aaSbaaeaacqaIYaGmaeqaaaqabaaaleqaaOGaeiikaGIaemivaq1aaSbaaSqaaiabdAha2naaBaaameaacqWFXoqyaeqaaSGaeyOKH4QaemiCaahabeaakiabdYeamjabcMcaPiabgUcaRiqbdgfarzaajaWaaSbaaSqaaiabdAha2naaBaaameaacqWFXoqyaeqaaSGaeiilaWIaemODay3aaSbaaWqaaiab=j7aInaaBaaabaGaeGymaedabeaaaeqaaSGaeiilaWIaemODay3aaSbaaWqaaiab=j7aInaaBaaabaGaeGOmaidabeaaaeqaaaWcbeaakiabcIcaOiabdYeamjabcMcaPiaaxMaacaWLjaWaaeWaaeaacqaIXaqmcqaI1aqnaiaawIcacaGLPaaaaaa@AACF@

DIFF7=Qvβ1,vβ2(Tvβ1→pSvβ1↔vβ2DvγL)−Qvβ1,vβ2(Tvβ1→pDvγL)−Qvβ1,vβ2(Svβ1↔vβ2DvγL)+Qvβ1,vβ2(DvγL)+Qvβ1,vγ(Tvβ1→P(vβ2)Dvβ2L)−Qvβ1,vγ(Dvβ2L)     (16)
 MathType@MTEF@5@5@+=feaafiart1ev1aaatCvAUfKttLearuWrP9MDH5MBPbIqV92AaeXatLxBI9gBaebbnrfifHhDYfgasaacH8akY=wiFfYdH8Gipec8Eeeu0xXdbba9frFj0=OqFfea0dXdd9vqai=hGuQ8kuc9pgc9s8qqaq=dirpe0xb9q8qiLsFr0=vr0=vr0dc8meaabaqaciaacaGaaeqabaqabeGadaaakeaafaqaaeGadaaabaGaemiraqKaemysaKKaemOrayKaemOray0aaSbaaSqaaiabiEda3aqabaaakeaacqGH9aqpaeaacqWGrbqudaWgaaWcbaGaemODay3aaSbaaWqaaGGaciab=j7aInaaBaaabaGaeGymaedabeaaaeqaaSGaeiilaWIaemODay3aaSbaaWqaaiab=j7aInaaBaaabaGaeGOmaidabeaaaeqaaaWcbeaakiabcIcaOiabdsfaunaaBaaaleaacqWG2bGDdaWgaaadbaGae8NSdi2aaSbaaeaacqaIXaqmaeqaaaqabaWccqGHsgIRcqWGWbaCaeqaaOGaem4uam1aaSbaaSqaaiabdAha2naaBaaameaacqWFYoGydaWgaaqaaiabigdaXaqabaaabeaaliabgsziRkabdAha2naaBaaameaacqWFYoGydaWgaaqaaiabikdaYaqabaaabeaaaSqabaGccqWGebardaWgaaWcbaGaemODay3aaSbaaWqaaiab=n7aNbqabaaaleqaaOGaemitaWKaeiykaKIaeyOeI0Iaemyuae1aaSbaaSqaaiabdAha2naaBaaameaacqWFYoGydaWgaaqaaiabigdaXaqabaaabeaaliabcYcaSiabdAha2naaBaaameaacqWFYoGydaWgaaqaaiabikdaYaqabaaabeaaaSqabaGccqGGOaakcqWGubavdaWgaaWcbaGaemODay3aaSbaaWqaaiab=j7aInaaBaaabaGaeGymaedabeaaaeqaaSGaeyOKH4QaemiCaahabeaakiabdseaenaaBaaaleaacqWG2bGDdaWgaaadbaGae83SdCgabeaaaSqabaGccqWGmbatcqGGPaqkcqGHsislcqWGrbqudaWgaaWcbaGaemODay3aaSbaaWqaaiab=j7aInaaBaaabaGaeGymaedabeaaaeqaaSGaeiilaWIaemODay3aaSbaaWqaaiab=j7aInaaBaaabaGaeGOmaidabeaaaeqaaaWcbeaakiabcIcaOiabdofatnaaBaaaleaacqWG2bGDdaWgaaadbaGae8NSdi2aaSbaaeaacqaIXaqmaeqaaaqabaWccqGHugYQcqWG2bGDdaWgaaadbaGae8NSdi2aaSbaaeaacqaIYaGmaeqaaaqabaaaleqaaOGaemiraq0aaSbaaSqaaiabdAha2naaBaaameaacqWFZoWzaeqaaaWcbeaakiabdYeamjabcMcaPaqaaaqaaiabgUcaRaqaaiabdgfarnaaBaaaleaacqWG2bGDdaWgaaadbaGae8NSdi2aaSbaaeaacqaIXaqmaeqaaaqabaWccqGGSaalcqWG2bGDdaWgaaadbaGae8NSdi2aaSbaaeaacqaIYaGmaeqaaaqabaaaleqaaOGaeiikaGIaemiraq0aaSbaaSqaaiabdAha2naaBaaameaacqWFZoWzaeqaaaWcbeaakiabdYeamjabcMcaPiabgUcaRiabdgfarnaaBaaaleaacqWG2bGDdaWgaaadbaGae8NSdi2aaSbaaeaacqaIXaqmaeqaaaqabaWccqGGSaalcqWG2bGDdaWgaaadbaGae83SdCgabeaaaSqabaGccqGGOaakcqWGubavdaWgaaWcbaGaemODay3aaSbaaWqaaiab=j7aInaaBaaabaGaeGymaedabeaaaeqaaSGaeyOKH4QaemiuaaLaeiikaGIaemODay3aaSbaaWqaaiab=j7aInaaBaaabaGaeGOmaidabeaaaeqaaSGaeiykaKcabeaakiabdseaenaaBaaaleaacqWG2bGDdaWgaaadbaGae8NSdi2aaSbaaeaacqaIYaGmaeqaaaqabaaaleqaaOGaemitaWKaeiykaKIaeyOeI0Iaemyuae1aaSbaaSqaaiabdAha2naaBaaameaacqWFYoGydaWgaaqaaiabigdaXaqabaaabeaaliabcYcaSiabdAha2naaBaaameaacqWFZoWzaeqaaaWcbeaakiabcIcaOiabdseaenaaBaaaleaacqWG2bGDdaWgaaadbaGae8NSdi2aaSbaaeaacqaIYaGmaeqaaaqabaaaleqaaOGaemitaWKaeiykaKcaaiaaxMaacaWLjaWaaeWaaeaacqaIXaqmcqaI2aGnaiaawIcacaGLPaaaaaa@E3FB@

DIFF8=Q^vα,vβ1,vβ2(Tvα→pSvβ1↔vβ2DvγL)−Q^vα,vβ1,vβ2(Svβ1↔vβ2DvγL)−Q^vα,vβ1,vβ2(Tvα→pDvγL)+Q^vα,vβ1,vβ2(DvγL)     (17)
 MathType@MTEF@5@5@+=feaafiart1ev1aaatCvAUfKttLearuWrP9MDH5MBPbIqV92AaeXatLxBI9gBaebbnrfifHhDYfgasaacH8akY=wiFfYdH8Gipec8Eeeu0xXdbba9frFj0=OqFfea0dXdd9vqai=hGuQ8kuc9pgc9s8qqaq=dirpe0xb9q8qiLsFr0=vr0=vr0dc8meaabaqaciaacaGaaeqabaqabeGadaaakeaafaqaaeGadaaabaGaemiraqKaemysaKKaemOrayKaemOray0aaSbaaSqaaiabiIda4aqabaaakeaacqGH9aqpaeaacuWGrbqugaqcamaaBaaaleaacqWG2bGDdaWgaaadbaacciGae8xSdegabeaaliabcYcaSiabdAha2naaBaaameaacqWFYoGydaWgaaqaaiabigdaXaqabaaabeaaliabcYcaSiabdAha2naaBaaameaacqWFYoGydaWgaaqaaiabikdaYaqabaaabeaaaSqabaGccqGGOaakcqWGubavdaWgaaWcbaGaemODay3aaSbaaWqaaiab=f7aHbqabaWccqGHsgIRcqWGWbaCaeqaaOGaem4uam1aaSbaaSqaaiabdAha2naaBaaameaacqWFYoGydaWgaaqaaiabigdaXaqabaaabeaaliabgsziRkabdAha2naaBaaameaacqWFYoGydaWgaaqaaiabikdaYaqabaaabeaaaSqabaGccqWGebardaWgaaWcbaGaemODay3aaSbaaWqaaiab=n7aNbqabaaaleqaaOGaemitaWKaeiykaKIaeyOeI0IafmyuaeLbaKaadaWgaaWcbaGaemODay3aaSbaaWqaaiab=f7aHbqabaWccqGGSaalcqWG2bGDdaWgaaadbaGae8NSdi2aaSbaaeaacqaIXaqmaeqaaaqabaWccqGGSaalcqWG2bGDdaWgaaadbaGae8NSdi2aaSbaaeaacqaIYaGmaeqaaaqabaaaleqaaOGaeiikaGIaem4uam1aaSbaaSqaaiabdAha2naaBaaameaacqWFYoGydaWgaaqaaiabigdaXaqabaaabeaaliabgsziRkabdAha2naaBaaameaacqWFYoGydaWgaaqaaiabikdaYaqabaaabeaaaSqabaGccqWGebardaWgaaWcbaGaemODay3aaSbaaWqaaiab=n7aNbqabaaaleqaaOGaemitaWKaeiykaKIaeyOeI0IafmyuaeLbaKaadaWgaaWcbaGaemODay3aaSbaaWqaaiab=f7aHbqabaWccqGGSaalcqWG2bGDdaWgaaadbaGae8NSdi2aaSbaaeaacqaIXaqmaeqaaaqabaWccqGGSaalcqWG2bGDdaWgaaadbaGae8NSdi2aaSbaaeaacqaIYaGmaeqaaaqabaaaleqaaOGaeiikaGIaemivaq1aaSbaaSqaaiabdAha2naaBaaameaacqWFXoqyaeqaaSGaeyOKH4QaemiCaahabeaakiabdseaenaaBaaaleaacqWG2bGDdaWgaaadbaGae83SdCgabeaaaSqabaGccqWGmbatcqGGPaqkaeaaaeaacqGHRaWkaeaacuWGrbqugaqcamaaBaaaleaacqWG2bGDdaWgaaadbaGae8xSdegabeaaliabcYcaSiabdAha2naaBaaameaacqWFYoGydaWgaaqaaiabigdaXaqabaaabeaaliabcYcaSiabdAha2naaBaaameaacqWFYoGydaWgaaqaaiabikdaYaqabaaabeaaaSqabaGccqGGOaakcqWGebardaWgaaWcbaGaemODay3aaSbaaWqaaiab=n7aNbqabaaaleqaaOGaemitaWKaeiykaKcaaiaaxMaacaWLjaWaaeWaaeaacqaIXaqmcqaI3aWnaiaawIcacaGLPaaaaaa@BD41@

DIFF9=Qvα,vβ2(Tvα→P(vβ2)Tvβ2→P(vβ1)Dvβ1L)−Qvα,vβ2(Tvβ2→P(vβ1)Dvβ1L)−Qvα,vβ1(Tvα→P(vβ2)Dvβ2L)+Qvα,vβ1(Dvβ2L).     (18)
 MathType@MTEF@5@5@+=feaafiart1ev1aaatCvAUfKttLearuWrP9MDH5MBPbIqV92AaeXatLxBI9gBaebbnrfifHhDYfgasaacH8akY=wiFfYdH8Gipec8Eeeu0xXdbba9frFj0=OqFfea0dXdd9vqai=hGuQ8kuc9pgc9s8qqaq=dirpe0xb9q8qiLsFr0=vr0=vr0dc8meaabaqaciaacaGaaeqabaqabeGadaaakeaafaqaaeGadaaabaGaemiraqKaemysaKKaemOrayKaemOray0aaSbaaSqaaiabiMda5aqabaaakeaacqGH9aqpaeaacqWGrbqudaWgaaWcbaGaemODay3aaSbaaWqaaGGaciab=f7aHbqabaWccqGGSaalcqWG2bGDdaWgaaadbaGae8NSdi2aaSbaaeaaieaacqGFYaGmaeqaaaqabaaaleqaaOGaeiikaGIaemivaq1aaSbaaSqaaiabdAha2naaBaaameaacqWFXoqyaeqaaSGaeyOKH4QaemiuaaLaeiikaGIaemODay3aaSbaaWqaaiab=j7aInaaBaaabaGaeGOmaidabeaaaeqaaSGaeiykaKcabeaakiabdsfaunaaBaaaleaacqWG2bGDdaWgaaadbaGae8NSdi2aaSbaaeaacqaIYaGmaeqaaaqabaWccqGHsgIRcqWGqbaucqGGOaakcqWG2bGDdaWgaaadbaGae8NSdi2aaSbaaeaacqaIXaqmaeqaaaqabaWccqGGPaqkaeqaaOGaemiraq0aaSbaaSqaaiabdAha2naaBaaameaacqWFYoGydaWgaaqaaiabigdaXaqabaaabeaaaSqabaGccqWGmbatcqGGPaqkcqGHsislcqWGrbqudaWgaaWcbaGaemODay3aaSbaaWqaaiab=f7aHbqabaWccqGGSaalcqWG2bGDdaWgaaadbaGae8NSdi2aaSbaaeaacqGFYaGmaeqaaaqabaaaleqaaOGaeiikaGIaemivaq1aaSbaaSqaaiabdAha2naaBaaameaacqWFYoGydaWgaaqaaiabikdaYaqabaaabeaaliabgkziUkabdcfaqjabcIcaOiabdAha2naaBaaameaacqWFYoGydaWgaaqaaiabigdaXaqabaaabeaaliabcMcaPaqabaGccqWGebardaWgaaWcbaGaemODay3aaSbaaWqaaiab=j7aInaaBaaabaGaeGymaedabeaaaeqaaaWcbeaakiabdYeamjabcMcaPaqaaaqaaiabgkHiTaqaaiabdgfarnaaBaaaleaacqWG2bGDdaWgaaadbaGae8xSdegabeaaliabcYcaSiabdAha2naaBaaameaacqWFYoGydaWgaaqaaiab+fdaXaqabaaabeaaaSqabaGccqGGOaakcqWGubavdaWgaaWcbaGaemODay3aaSbaaWqaaiab=f7aHbqabaWccqGHsgIRcqWGqbaucqGGOaakcqWG2bGDdaWgaaadbaGae8NSdi2aaSbaaeaacqaIYaGmaeqaaaqabaWccqGGPaqkaeqaaOGaemiraq0aaSbaaSqaaiabdAha2naaBaaameaacqWFYoGydaWgaaqaaiabikdaYaqabaaabeaaaSqabaGccqWGmbatcqGGPaqkcqGHRaWkcqWGrbqudaWgaaWcbaGaemODay3aaSbaaWqaaiab=f7aHbqabaWccqGGSaalcqWG2bGDdaWgaaadbaGae8NSdi2aaSbaaeaacqGFXaqmaeqaaaqabaaaleqaaOGaeiikaGIaemiraq0aaSbaaSqaaiabdAha2naaBaaameaacqWFYoGydaWgaaqaaiabikdaYaqabaaabeaaaSqabaGccqWGmbatcqGGPaqkcqGGUaGlaaGaaCzcaiaaxMaadaqadaqaaiabigdaXiabiIda4aGaayjkaiaawMcaaaaa@BE66@

The case of *v*_*α *_= vβ2
 MathType@MTEF@5@5@+=feaafiart1ev1aaatCvAUfKttLearuWrP9MDH5MBPbIqV92AaeXatLxBI9gBaebbnrfifHhDYfgasaacH8akY=wiFfYdH8Gipec8Eeeu0xXdbba9frFj0=OqFfea0dXdd9vqai=hGuQ8kuc9pgc9s8qqaq=dirpe0xb9q8qiLsFr0=vr0=vr0dc8meaabaqaciaacaGaaeqabaqabeGadaaakeaacqWG2bGDdaWgaaWcbaacciGae8NSdi2aaSbaaWqaaiabikdaYaqabaaaleqaaaaa@311F@ is not considered in Equation (13) because equations of this case can be obtained by simply replacing vβ1
 MathType@MTEF@5@5@+=feaafiart1ev1aaatCvAUfKttLearuWrP9MDH5MBPbIqV92AaeXatLxBI9gBaebbnrfifHhDYfgasaacH8akY=wiFfYdH8Gipec8Eeeu0xXdbba9frFj0=OqFfea0dXdd9vqai=hGuQ8kuc9pgc9s8qqaq=dirpe0xb9q8qiLsFr0=vr0=vr0dc8meaabaqaciaacaGaaeqabaqabeGadaaakeaacqWG2bGDdaWgaaWcbaacciGae8NSdi2aaSbaaWqaaiabigdaXaqabaaaleqaaaaa@311D@ with vβ2
 MathType@MTEF@5@5@+=feaafiart1ev1aaatCvAUfKttLearuWrP9MDH5MBPbIqV92AaeXatLxBI9gBaebbnrfifHhDYfgasaacH8akY=wiFfYdH8Gipec8Eeeu0xXdbba9frFj0=OqFfea0dXdd9vqai=hGuQ8kuc9pgc9s8qqaq=dirpe0xb9q8qiLsFr0=vr0=vr0dc8meaabaqaciaacaGaaeqabaqabeGadaaakeaacqWG2bGDdaWgaaWcbaacciGae8NSdi2aaSbaaWqaaiabikdaYaqabaaaleqaaaaa@311F@ in case 1 and 3.

Qva,vb
 MathType@MTEF@5@5@+=feaafiart1ev1aaatCvAUfKttLearuWrP9MDH5MBPbIqV92AaeXatLxBI9gBaebbnrfifHhDYfgasaacH8akY=wiFfYdH8Gipec8Eeeu0xXdbba9frFj0=OqFfea0dXdd9vqai=hGuQ8kuc9pgc9s8qqaq=dirpe0xb9q8qiLsFr0=vr0=vr0dc8meaabaqaciaacaGaaeqabaqabeGadaaakeaacqWGrbqudaWgaaWcbaGaemODay3aaSbaaWqaaiabdggaHbqabaWccqGGSaalcqWG2bGDdaWgaaadbaGaemOyaigabeaaaSqabaaaaa@34D5@ (·) and Q^va,vb,vc
 MathType@MTEF@5@5@+=feaafiart1ev1aaatCvAUfKttLearuWrP9MDH5MBPbIqV92AaeXatLxBI9gBaebbnrfifHhDYfgasaacH8akY=wiFfYdH8Gipec8Eeeu0xXdbba9frFj0=OqFfea0dXdd9vqai=hGuQ8kuc9pgc9s8qqaq=dirpe0xb9q8qiLsFr0=vr0=vr0dc8meaabaqaciaacaGaaeqabaqabeGadaaakeaacuWGrbqugaqcamaaBaaaleaacqWG2bGDdaWgaaadbaGaemyyaegabeaaliabcYcaSiabdAha2naaBaaameaacqWGIbGyaeqaaSGaeiilaWIaemODay3aaSbaaWqaaiabdogaJbqabaaaleqaaaaa@38C1@ (·) in *DIFF*_0 _to *DIFF*_9 _are partial cost functions depending on the two nodes *v*_*a *_and *v*_*b *_and the three nodes *v*_*a*_, *v*_*b*_, and *v*_*c*_, respectively, they are the sums of the corresponding partial edge-edge crossing costs, node-edge crossing costs and distance costs as follows:

Qva,vb(L)=WvQva,vbdc(L)+WeeQva,vbee(L)+WveQva,vbve(L)     (19)
 MathType@MTEF@5@5@+=feaafiart1ev1aaatCvAUfKttLearuWrP9MDH5MBPbIqV92AaeXatLxBI9gBaebbnrfifHhDYfgasaacH8akY=wiFfYdH8Gipec8Eeeu0xXdbba9frFj0=OqFfea0dXdd9vqai=hGuQ8kuc9pgc9s8qqaq=dirpe0xb9q8qiLsFr0=vr0=vr0dc8meaabaqaciaacaGaaeqabaqabeGadaaakeaacqWGrbqudaWgaaWcbaGaemODay3aaSbaaWqaaiabdggaHbqabaWccqGGSaalcqWG2bGDdaWgaaadbaGaemOyaigabeaaaSqabaGccqGGOaakcqWGmbatcqGGPaqkcqGH9aqpcqWGxbWvdaWgaaWcbaGaemODayhabeaakiabdgfarnaaDaaaleaacqWG2bGDdaWgaaadbaGaemyyaegabeaaliabcYcaSiabdAha2naaBaaameaacqWGIbGyaeqaaaWcbaGaemizaqMaem4yamgaaOGaeiikaGIaemitaWKaeiykaKIaey4kaSIaem4vaC1aaSbaaSqaaiabdwgaLjabdwgaLbqabaGccqWGrbqudaqhaaWcbaGaemODay3aaSbaaWqaaiabdggaHbqabaWccqGGSaalcqWG2bGDdaWgaaadbaGaemOyaigabeaaaSqaaiabdwgaLjabdwgaLbaakiabcIcaOiabdYeamjabcMcaPiabgUcaRiabdEfaxnaaBaaaleaacqWG2bGDcqWGLbqzaeqaaOGaemyuae1aa0baaSqaaiabdAha2naaBaaameaacqWGHbqyaeqaaSGaeiilaWIaemODay3aaSbaaWqaaiabdkgaIbqabaaaleaacqWG2bGDcqWGLbqzaaGccqGGOaakcqWGmbatcqGGPaqkcaWLjaGaaCzcamaabmaabaGaeGymaeJaeGyoaKdacaGLOaGaayzkaaaaaa@7386@

Q^va,vb,vc(L)=WvQ^va,vb,vcdc(L)+WeeQ^va,vb,vcee(L)+WveQ^va,vb,vcve(L),     (20)
 MathType@MTEF@5@5@+=feaafiart1ev1aaatCvAUfKttLearuWrP9MDH5MBPbIqV92AaeXatLxBI9gBaebbnrfifHhDYfgasaacH8akY=wiFfYdH8Gipec8Eeeu0xXdbba9frFj0=OqFfea0dXdd9vqai=hGuQ8kuc9pgc9s8qqaq=dirpe0xb9q8qiLsFr0=vr0=vr0dc8meaabaqaciaacaGaaeqabaqabeGadaaakeaacuWGrbqugaqcamaaBaaaleaacqWG2bGDdaWgaaadbaGaemyyaegabeaaliabcYcaSiabdAha2naaBaaameaacqWGIbGyaeqaaSGaeiilaWIaemODay3aaSbaaWqaaiabdogaJbqabaaaleqaaOGaeiikaGIaemitaWKaeiykaKIaeyypa0Jaem4vaC1aaSbaaSqaaiabdAha2bqabaGccuWGrbqugaqcamaaDaaaleaacqWG2bGDdaWgaaadbaGaemyyaegabeaaliabcYcaSiabdAha2naaBaaameaacqWGIbGyaeqaaSGaeiilaWIaemODay3aaSbaaWqaaiabdogaJbqabaaaleaacqWGKbazcqWGJbWyaaGccqGGOaakcqWGmbatcqGGPaqkcqGHRaWkcqWGxbWvdaWgaaWcbaGaemyzauMaemyzaugabeaakiqbdgfarzaajaWaa0baaSqaaiabdAha2naaBaaameaacqWGHbqyaeqaaSGaeiilaWIaemODay3aaSbaaWqaaiabdkgaIbqabaWccqGGSaalcqWG2bGDdaWgaaadbaGaem4yamgabeaaaSqaaiabdwgaLjabdwgaLbaakiabcIcaOiabdYeamjabcMcaPiabgUcaRiabdEfaxnaaBaaaleaacqWG2bGDcqWGLbqzaeqaaOGafmyuaeLbaKaadaqhaaWcbaGaemODay3aaSbaaWqaaiabdggaHbqabaWccqGGSaalcqWG2bGDdaWgaaadbaGaemOyaigabeaaliabcYcaSiabdAha2naaBaaameaacqWGJbWyaeqaaaWcbaGaemODayNaemyzaugaaOGaeiikaGIaemitaWKaeiykaKIaeiilaWIaaCzcaiaaxMaadaqadaqaaiabikdaYiabicdaWaGaayjkaiaawMcaaaaa@8406@

where Qva,vbee
 MathType@MTEF@5@5@+=feaafiart1ev1aaatCvAUfKttLearuWrP9MDH5MBPbIqV92AaeXatLxBI9gBaebbnrfifHhDYfgasaacH8akY=wiFfYdH8Gipec8Eeeu0xXdbba9frFj0=OqFfea0dXdd9vqai=hGuQ8kuc9pgc9s8qqaq=dirpe0xb9q8qiLsFr0=vr0=vr0dc8meaabaqaciaacaGaaeqabaqabeGadaaakeaacqWGrbqudaqhaaWcbaGaemODay3aaSbaaWqaaiabdggaHbqabaWccqGGSaalcqWG2bGDdaWgaaadbaGaemOyaigabeaaaSqaaiabdwgaLjabdwgaLbaaaaa@377C@ (·) and Q^va,vb,vcee
 MathType@MTEF@5@5@+=feaafiart1ev1aaatCvAUfKttLearuWrP9MDH5MBPbIqV92AaeXatLxBI9gBaebbnrfifHhDYfgasaacH8akY=wiFfYdH8Gipec8Eeeu0xXdbba9frFj0=OqFfea0dXdd9vqai=hGuQ8kuc9pgc9s8qqaq=dirpe0xb9q8qiLsFr0=vr0=vr0dc8meaabaqaciaacaGaaeqabaqabeGadaaakeaacuWGrbqugaqcamaaDaaaleaacqWG2bGDdaWgaaadbaGaemyyaegabeaaliabcYcaSiabdAha2naaBaaameaacqWGIbGyaeqaaSGaeiilaWIaemODay3aaSbaaWqaaiabdogaJbqabaaaleaacqWGLbqzcqWGLbqzaaaaaa@3B68@ (·) are related to edge-edge crossings, while Qva,vbne
 MathType@MTEF@5@5@+=feaafiart1ev1aaatCvAUfKttLearuWrP9MDH5MBPbIqV92AaeXatLxBI9gBaebbnrfifHhDYfgasaacH8akY=wiFfYdH8Gipec8Eeeu0xXdbba9frFj0=OqFfea0dXdd9vqai=hGuQ8kuc9pgc9s8qqaq=dirpe0xb9q8qiLsFr0=vr0=vr0dc8meaabaqaciaacaGaaeqabaqabeGadaaakeaacqWGrbqudaqhaaWcbaGaemODay3aaSbaaWqaaiabdggaHbqabaWccqGGSaalcqWG2bGDdaWgaaadbaGaemOyaigabeaaaSqaaiabd6gaUjabdwgaLbaaaaa@378E@ (·) and Q^va,vb,vcne
 MathType@MTEF@5@5@+=feaafiart1ev1aaatCvAUfKttLearuWrP9MDH5MBPbIqV92AaeXatLxBI9gBaebbnrfifHhDYfgasaacH8akY=wiFfYdH8Gipec8Eeeu0xXdbba9frFj0=OqFfea0dXdd9vqai=hGuQ8kuc9pgc9s8qqaq=dirpe0xb9q8qiLsFr0=vr0=vr0dc8meaabaqaciaacaGaaeqabaqabeGadaaakeaacuWGrbqugaqcamaaDaaaleaacqWG2bGDdaWgaaadbaGaemyyaegabeaaliabcYcaSiabdAha2naaBaaameaacqWGIbGyaeqaaSGaeiilaWIaemODay3aaSbaaWqaaiabdogaJbqabaaaleaacqWGUbGBcqWGLbqzaaaaaa@3B7A@ (·) are related to node-edge crossings, and Qva,vbdc
 MathType@MTEF@5@5@+=feaafiart1ev1aaatCvAUfKttLearuWrP9MDH5MBPbIqV92AaeXatLxBI9gBaebbnrfifHhDYfgasaacH8akY=wiFfYdH8Gipec8Eeeu0xXdbba9frFj0=OqFfea0dXdd9vqai=hGuQ8kuc9pgc9s8qqaq=dirpe0xb9q8qiLsFr0=vr0=vr0dc8meaabaqaciaacaGaaeqabaqabeGadaaakeaacqWGrbqudaqhaaWcbaGaemODay3aaSbaaWqaaiabdggaHbqabaWccqGGSaalcqWG2bGDdaWgaaadbaGaemOyaigabeaaaSqaaiabdsgaKjabdogaJbaaaaa@3776@ (·) and Q^va,vb,vcdc
 MathType@MTEF@5@5@+=feaafiart1ev1aaatCvAUfKttLearuWrP9MDH5MBPbIqV92AaeXatLxBI9gBaebbnrfifHhDYfgasaacH8akY=wiFfYdH8Gipec8Eeeu0xXdbba9frFj0=OqFfea0dXdd9vqai=hGuQ8kuc9pgc9s8qqaq=dirpe0xb9q8qiLsFr0=vr0=vr0dc8meaabaqaciaacaGaaeqabaqabeGadaaakeaacuWGrbqugaqcamaaDaaaleaacqWG2bGDdaWgaaadbaGaemyyaegabeaaliabcYcaSiabdAha2naaBaaameaacqWGIbGyaeqaaSGaeiilaWIaemODay3aaSbaaWqaaiabdogaJbqabaaaleaacqWGKbazcqWGJbWyaaaaaa@3B62@ (·) are related to the distance cost. The details are described as below.

(a) Qva,vbee
 MathType@MTEF@5@5@+=feaafiart1ev1aaatCvAUfKttLearuWrP9MDH5MBPbIqV92AaeXatLxBI9gBaebbnrfifHhDYfgasaacH8akY=wiFfYdH8Gipec8Eeeu0xXdbba9frFj0=OqFfea0dXdd9vqai=hGuQ8kuc9pgc9s8qqaq=dirpe0xb9q8qiLsFr0=vr0=vr0dc8meaabaqaciaacaGaaeqabaqabeGadaaakeaacqWGrbqudaqhaaWcbaGaemODay3aaSbaaWqaaiabdggaHbqabaWccqGGSaalcqWG2bGDdaWgaaadbaGaemOyaigabeaaaSqaaiabdwgaLjabdwgaLbaaaaa@377C@ (·) is a partial edge-edge crossing cost function of Eva
 MathType@MTEF@5@5@+=feaafiart1ev1aaatCvAUfKttLearuWrP9MDH5MBPbIqV92AaeXatLxBI9gBaebbnrfifHhDYfgasaacH8akY=wiFfYdH8Gipec8Eeeu0xXdbba9frFj0=OqFfea0dXdd9vqai=hGuQ8kuc9pgc9s8qqaq=dirpe0xb9q8qiLsFr0=vr0=vr0dc8meaabaqaciaacaGaaeqabaqabeGadaaakeaacqWGfbqrdaWgaaWcbaGaemODay3aaSbaaWqaaiabdggaHbqabaaaleqaaaaa@30E3@ and Evb
 MathType@MTEF@5@5@+=feaafiart1ev1aaatCvAUfKttLearuWrP9MDH5MBPbIqV92AaeXatLxBI9gBaebbnrfifHhDYfgasaacH8akY=wiFfYdH8Gipec8Eeeu0xXdbba9frFj0=OqFfea0dXdd9vqai=hGuQ8kuc9pgc9s8qqaq=dirpe0xb9q8qiLsFr0=vr0=vr0dc8meaabaqaciaacaGaaeqabaqabeGadaaakeaacqWGfbqrdaWgaaWcbaGaemODay3aaSbaaWqaaiabdkgaIbqabaaaleqaaaaa@30E5@, and is defined as follows:

Qva,vbee(L)={∑eva∈Eva,evb∈EvbCrosseva,evb(L)if (va,vb)∉E∑eva∈Eva,evb∈EvbCrosseva,evb(L)+∑e∈ECrosse,(va,vb)(L)if (va,vb)∈E.     (21)
 MathType@MTEF@5@5@+=feaafiart1ev1aaatCvAUfKttLearuWrP9MDH5MBPbIqV92AaeXatLxBI9gBaebbnrfifHhDYfgasaacH8akY=wiFfYdH8Gipec8Eeeu0xXdbba9frFj0=OqFfea0dXdd9vqai=hGuQ8kuc9pgc9s8qqaq=dirpe0xb9q8qiLsFr0=vr0=vr0dc8meaabaqaciaacaGaaeqabaqabeGadaaakeaacqWGrbqudaqhaaWcbaGaemODay3aaSbaaWqaaiabdggaHbqabaWccqGGSaalcqWG2bGDdaWgaaadbaGaemOyaigabeaaaSqaaiabdwgaLjabdwgaLbaakiabcIcaOiabdYeamjabcMcaPiabg2da9maaceqabaqbaeaabiGaaaqaamaaqafabaGaem4qamKaemOCaiNaem4Ba8Maem4CamNaem4Cam3aaSbaaSqaaiabdwgaLnaaBaaameaacqWG2bGDdaWgaaqaaiabdggaHbqabaaabeaaliabcYcaSiabdwgaLnaaBaaameaacqWG2bGDdaWgaaqaaiabdkgaIbqabaaabeaaaSqabaGccqGGOaakcqWGmbatcqGGPaqkaSqaaiabdwgaLnaaBaaameaacqWG2bGDdaWgaaqaaiabdggaHbqabaaabeaaliabgIGiolabdweafnaaBaaameaacqWG2bGDdaWgaaqaaiabdggaHbqabaaabeaaliabcYcaSiabdwgaLnaaBaaameaacqWG2bGDdaWgaaqaaiabdkgaIbqabaaabeaaliabgIGiolabdweafnaaBaaameaacqWG2bGDdaWgaaqaaiabdkgaIbqabaaabeaaaSqab0GaeyyeIuoaaOqaaiabbMgaPjabbAgaMjabbccaGiabcIcaOiabdAha2naaBaaaleaacqWGHbqyaeqaaOGaeiilaWIaemODay3aaSbaaSqaaiabdkgaIbqabaGccqGGPaqkcqGHjiYZcqWGfbqraeaadaaeqbqaaiabdoeadjabdkhaYjabd+gaVjabdohaZjabdohaZnaaBaaaleaacqWGLbqzdaWgaaadbaGaemODay3aaSbaaeaacqWGHbqyaeqaaaqabaWccqGGSaalcqWGLbqzdaWgaaadbaGaemODay3aaSbaaeaacqWGIbGyaeqaaaqabaaaleqaaOGaeiikaGIaemitaWKaeiykaKcaleaacqWGLbqzdaWgaaadbaGaemODay3aaSbaaeaacqWGHbqyaeqaaaqabaWccqGHiiIZcqWGfbqrdaWgaaadbaGaemODay3aaSbaaeaacqWGHbqyaeqaaaqabaWccqGGSaalcqWGLbqzdaWgaaadbaGaemODay3aaSbaaeaacqWGIbGyaeqaaaqabaWccqGHiiIZcqWGfbqrdaWgaaadbaGaemODay3aaSbaaeaacqWGIbGyaeqaaaqabaaaleqaniabggHiLdGccqGHRaWkdaaeqbqaaiabdoeadjabdkhaYjabd+gaVjabdohaZjabdohaZnaaBaaaleaacqWGLbqzcqGGSaalcqGGOaakcqWG2bGDdaWgaaadbaGaemyyaegabeaaliabcYcaSiabdAha2naaBaaameaacqWGIbGyaeqaaSGaeiykaKcabeaakiabcIcaOiabdYeamjabcMcaPaWcbaGaemyzauMaeyicI4SaemyraueabeqdcqGHris5aaGcbaGaeeyAaKMaeeOzayMaeeiiaaIaeiikaGIaemODay3aaSbaaSqaaiabdggaHbqabaGccqGGSaalcqWG2bGDdaWgaaWcbaGaemOyaigabeaakiabcMcaPiabgIGiolabdweafbaaaiaawUhaaiabc6caUiaaxMaacaWLjaWaaeWaaeaacqaIYaGmcqaIXaqmaiaawIcacaGLPaaaaaa@D046@

Similarly, Q^va,vb,vcee
 MathType@MTEF@5@5@+=feaafiart1ev1aaatCvAUfKttLearuWrP9MDH5MBPbIqV92AaeXatLxBI9gBaebbnrfifHhDYfgasaacH8akY=wiFfYdH8Gipec8Eeeu0xXdbba9frFj0=OqFfea0dXdd9vqai=hGuQ8kuc9pgc9s8qqaq=dirpe0xb9q8qiLsFr0=vr0=vr0dc8meaabaqaciaacaGaaeqabaqabeGadaaakeaacuWGrbqugaqcamaaDaaaleaacqWG2bGDdaWgaaadbaGaemyyaegabeaaliabcYcaSiabdAha2naaBaaameaacqWGIbGyaeqaaSGaeiilaWIaemODay3aaSbaaWqaaiabdogaJbqabaaaleaacqWGLbqzcqWGLbqzaaaaaa@3B68@ (·) is a partial edge-edge crossing cost function of Eva
 MathType@MTEF@5@5@+=feaafiart1ev1aaatCvAUfKttLearuWrP9MDH5MBPbIqV92AaeXatLxBI9gBaebbnrfifHhDYfgasaacH8akY=wiFfYdH8Gipec8Eeeu0xXdbba9frFj0=OqFfea0dXdd9vqai=hGuQ8kuc9pgc9s8qqaq=dirpe0xb9q8qiLsFr0=vr0=vr0dc8meaabaqaciaacaGaaeqabaqabeGadaaakeaacqWGfbqrdaWgaaWcbaGaemODay3aaSbaaWqaaiabdggaHbqabaaaleqaaaaa@30E3@, Evb
 MathType@MTEF@5@5@+=feaafiart1ev1aaatCvAUfKttLearuWrP9MDH5MBPbIqV92AaeXatLxBI9gBaebbnrfifHhDYfgasaacH8akY=wiFfYdH8Gipec8Eeeu0xXdbba9frFj0=OqFfea0dXdd9vqai=hGuQ8kuc9pgc9s8qqaq=dirpe0xb9q8qiLsFr0=vr0=vr0dc8meaabaqaciaacaGaaeqabaqabeGadaaakeaacqWGfbqrdaWgaaWcbaGaemODay3aaSbaaWqaaiabdkgaIbqabaaaleqaaaaa@30E5@, and Evc
 MathType@MTEF@5@5@+=feaafiart1ev1aaatCvAUfKttLearuWrP9MDH5MBPbIqV92AaeXatLxBI9gBaebbnrfifHhDYfgasaacH8akY=wiFfYdH8Gipec8Eeeu0xXdbba9frFj0=OqFfea0dXdd9vqai=hGuQ8kuc9pgc9s8qqaq=dirpe0xb9q8qiLsFr0=vr0=vr0dc8meaabaqaciaacaGaaeqabaqabeGadaaakeaacqWGfbqrdaWgaaWcbaGaemODay3aaSbaaWqaaiabdogaJbqabaaaleqaaaaa@30E7@, and is defined as follows:

Q^va,vb,vcee(L)={∑eva∈Eva,evb∈EvbCrosseva,evb(L)+∑eva∈Eva,evc∈EvcCrosseva,evc(L) (=Q^ee)if (va,vb)∉E,(va,vc)∉EQ^ee+∑e∈E\EvcCrosse,(va,vb)(L)if (va,vb)∈E,(va,vc)∉EQ^ee+∑e∈E\EvbCrosse,(va,vc)(L)if (va,vb)∉E,(va,vc)∈EQ^ee+∑e∈E\EvcCrosse,(va,vb)(L)+∑e∈E\EvbCrosse,(va,vc)(L)if (va,vb)∈E,(va,vc)∈E.     (22)
 MathType@MTEF@5@5@+=feaafiart1ev1aaatCvAUfKttLearuWrP9MDH5MBPbIqV92AaeXatLxBI9gBaebbnrfifHhDYfgasaacH8akY=wiFfYdH8Gipec8Eeeu0xXdbba9frFj0=OqFfea0dXdd9vqai=hGuQ8kuc9pgc9s8qqaq=dirpe0xb9q8qiLsFr0=vr0=vr0dc8meaabaqaciaacaGaaeqabaqabeGadaaakeaacuWGrbqugaqcamaaDaaaleaacqWG2bGDdaWgaaadbaGaemyyaegabeaaliabcYcaSiabdAha2naaBaaameaacqWGIbGyaeqaaSGaeiilaWIaemODay3aaSbaaWqaaiabdogaJbqabaaaleaacqWGLbqzcqWGLbqzaaGccqGGOaakcqWGmbatcqGGPaqkcqGH9aqpdaGabeqaauaabaqaeiaaaaqaauaabeqaceaaaeaadaaeqbqaaiabdoeadjabdkhaYjabd+gaVjabdohaZjabdohaZnaaBaaaleaacqWGLbqzdaWgaaadbaGaemODay3aaSbaaeaacqWGHbqyaeqaaaqabaWccqGGSaalcqWGLbqzdaWgaaadbaGaemODay3aaSbaaeaacqWGIbGyaeqaaaqabaaaleqaaOGaeiikaGIaemitaWKaeiykaKcaleaacqWGLbqzdaWgaaadbaGaemODay3aaSbaaeaacqWGHbqyaeqaaaqabaWccqGHiiIZcqWGfbqrdaWgaaadbaGaemODay3aaSbaaeaacqWGHbqyaeqaaaqabaWccqGGSaalcqWGLbqzdaWgaaadbaGaemODay3aaSbaaeaacqWGIbGyaeqaaaqabaWccqGHiiIZcqWGfbqrdaWgaaadbaGaemODay3aaSbaaeaacqWGIbGyaeqaaaqabaaaleqaniabggHiLdaakeaacqGHRaWkdaaeqbqaaiabdoeadjabdkhaYjabd+gaVjabdohaZjabdohaZnaaBaaaleaacqWGLbqzdaWgaaadbaGaemODay3aaSbaaeaacqWGHbqyaeqaaaqabaWccqGGSaalcqWGLbqzdaWgaaadbaGaemODay3aaSbaaeaacqWGJbWyaeqaaaqabaaaleqaaOGaeiikaGIaemitaWKaeiykaKcaleaacqWGLbqzdaWgaaadbaGaemODay3aaSbaaeaacqWGHbqyaeqaaaqabaWccqGHiiIZcqWGfbqrdaWgaaadbaGaemODay3aaSbaaeaacqWGHbqyaeqaaaqabaWccqGGSaalcqWGLbqzdaWgaaadbaGaemODay3aaSbaaeaacqWGJbWyaeqaaaqabaWccqGHiiIZcqWGfbqrdaWgaaadbaGaemODay3aaSbaaeaacqWGJbWyaeqaaaqabaaaleqaniabggHiLdaaaOGaeeiiaaIaeiikaGIaeyypa0JafmyuaeLbaKaadaahaaWcbeqaaiabdwgaLjabdwgaLbaakiabcMcaPaqaaiabbMgaPjabbAgaMjabbccaGuaabeqaceaaaeaacqGGOaakcqWG2bGDdaWgaaWcbaGaemyyaegabeaakiabcYcaSiabdAha2naaBaaaleaacqWGIbGyaeqaaOGaeiykaKIaeyycI8SaemyrauKaeiilaWcabaGaeiikaGIaemODay3aaSbaaSqaaiabdggaHbqabaGccqGGSaalcqWG2bGDdaWgaaWcbaGaem4yamgabeaakiabcMcaPiabgMGiplabdweafbaaaeaacuWGrbqugaqcamaaCaaaleqabaGaemyzauMaemyzaugaaOGaey4kaSYaaabuaeaacqWGdbWqcqWGYbGCcqWGVbWBcqWGZbWCcqWGZbWCdaWgaaWcbaGaemyzauMaeiilaWIaeiikaGIaemODay3aaSbaaWqaaiabdggaHbqabaWccqGGSaalcqWG2bGDdaWgaaadbaGaemOyaigabeaaliabcMcaPaqabaGccqGGOaakcqWGmbatcqGGPaqkaSqaaiabdwgaLjabgIGiolabdweafjabcYfaCjabdweafnaaBaaameaacqWG2bGDdaWgaaqaaiabdogaJbqabaaabeaaaSqab0GaeyyeIuoaaOqaaiabbMgaPjabbAgaMjabbccaGuaabeqaceaaaeaacqGGOaakcqWG2bGDdaWgaaWcbaGaemyyaegabeaakiabcYcaSiabdAha2naaBaaaleaacqWGIbGyaeqaaOGaeiykaKIaeyicI4SaemyrauKaeiilaWcabaGaeiikaGIaemODay3aaSbaaSqaaiabdggaHbqabaGccqGGSaalcqWG2bGDdaWgaaWcbaGaem4yamgabeaakiabcMcaPiabgMGiplabdweafbaaaeaacuWGrbqugaqcamaaCaaaleqabaGaemyzauMaemyzaugaaOGaey4kaSYaaabuaeaacqWGdbWqcqWGYbGCcqWGVbWBcqWGZbWCcqWGZbWCdaWgaaWcbaGaemyzauMaeiilaWIaeiikaGIaemODay3aaSbaaWqaaiabdggaHbqabaWccqGGSaalcqWG2bGDdaWgaaadbaGaem4yamgabeaaliabcMcaPaqabaGccqGGOaakcqWGmbatcqGGPaqkaSqaaiabdwgaLjabgIGiolabdweafjabcYfaCjabdweafnaaBaaameaacqWG2bGDdaWgaaqaaiabdkgaIbqabaaabeaaaSqab0GaeyyeIuoaaOqaaiabbMgaPjabbAgaMjabbccaGuaabeqaceaaaeaacqGGOaakcqWG2bGDdaWgaaWcbaGaemyyaegabeaakiabcYcaSiabdAha2naaBaaaleaacqWGIbGyaeqaaOGaeiykaKIaeyycI8SaemyrauKaeiilaWcabaGaeiikaGIaemODay3aaSbaaSqaaiabdggaHbqabaGccqGGSaalcqWG2bGDdaWgaaWcbaGaem4yamgabeaakiabcMcaPiabgIGiolabdweafbaaaeaacuWGrbqugaqcamaaCaaaleqabaGaemyzauMaemyzaugaaOGaey4kaSYaaabuaeaacqWGdbWqcqWGYbGCcqWGVbWBcqWGZbWCcqWGZbWCdaWgaaWcbaGaemyzauMaeiilaWIaeiikaGIaemODay3aaSbaaWqaaiabdggaHbqabaWccqGGSaalcqWG2bGDdaWgaaadbaGaemOyaigabeaaliabcMcaPaqabaGccqGGOaakcqWGmbatcqGGPaqkaSqaaiabdwgaLjabgIGiolabdweafjabcYfaCjabdweafnaaBaaameaacqWG2bGDdaWgaaqaaiabdogaJbqabaaabeaaaSqab0GaeyyeIuoakiabgUcaRmaaqafabaGaem4qamKaemOCaiNaem4Ba8Maem4CamNaem4Cam3aaSbaaSqaaiabdwgaLjabcYcaSiabcIcaOiabdAha2naaBaaameaacqWGHbqyaeqaaSGaeiilaWIaemODay3aaSbaaWqaaiabdogaJbqabaWccqGGPaqkaeqaaOGaeiikaGIaemitaWKaeiykaKcaleaacqWGLbqzcqGHiiIZcqWGfbqrcqGGCbaxcqWGfbqrdaWgaaadbaGaemODay3aaSbaaeaacqWGIbGyaeqaaaqabaaaleqaniabggHiLdaakeaacqqGPbqAcqqGMbGzcqqGGaaifaqabeGabaaabaGaeiikaGIaemODay3aaSbaaSqaaiabdggaHbqabaGccqGGSaalcqWG2bGDdaWgaaWcbaGaemOyaigabeaakiabcMcaPiabgIGiolabdweafjabcYcaSaqaaiabcIcaOiabdAha2naaBaaaleaacqWGHbqyaeqaaOGaeiilaWIaemODay3aaSbaaSqaaiabdogaJbqabaGccqGGPaqkcqGHiiIZcqWGfbqraaaaaaGaay5EaaGaeiOla4IaaCzcaiaaxMaadaqadaqaaiabikdaYiabikdaYaGaayjkaiaawMcaaaaa@9E6E@

(b) Qva,vbne
 MathType@MTEF@5@5@+=feaafiart1ev1aaatCvAUfKttLearuWrP9MDH5MBPbIqV92AaeXatLxBI9gBaebbnrfifHhDYfgasaacH8akY=wiFfYdH8Gipec8Eeeu0xXdbba9frFj0=OqFfea0dXdd9vqai=hGuQ8kuc9pgc9s8qqaq=dirpe0xb9q8qiLsFr0=vr0=vr0dc8meaabaqaciaacaGaaeqabaqabeGadaaakeaacqWGrbqudaqhaaWcbaGaemODay3aaSbaaWqaaiabdggaHbqabaWccqGGSaalcqWG2bGDdaWgaaadbaGaemOyaigabeaaaSqaaiabd6gaUjabdwgaLbaaaaa@378E@ is a partial node-edge crossing cost function of *v*_*a*_, *v*_*b*_, Eva
 MathType@MTEF@5@5@+=feaafiart1ev1aaatCvAUfKttLearuWrP9MDH5MBPbIqV92AaeXatLxBI9gBaebbnrfifHhDYfgasaacH8akY=wiFfYdH8Gipec8Eeeu0xXdbba9frFj0=OqFfea0dXdd9vqai=hGuQ8kuc9pgc9s8qqaq=dirpe0xb9q8qiLsFr0=vr0=vr0dc8meaabaqaciaacaGaaeqabaqabeGadaaakeaacqWGfbqrdaWgaaWcbaGaemODay3aaSbaaWqaaiabdggaHbqabaaaleqaaaaa@30E3@, and Evb
 MathType@MTEF@5@5@+=feaafiart1ev1aaatCvAUfKttLearuWrP9MDH5MBPbIqV92AaeXatLxBI9gBaebbnrfifHhDYfgasaacH8akY=wiFfYdH8Gipec8Eeeu0xXdbba9frFj0=OqFfea0dXdd9vqai=hGuQ8kuc9pgc9s8qqaq=dirpe0xb9q8qiLsFr0=vr0=vr0dc8meaabaqaciaacaGaaeqabaqabeGadaaakeaacqWGfbqrdaWgaaWcbaGaemODay3aaSbaaWqaaiabdkgaIbqabaaaleqaaaaa@30E5@, and is defined as follows:

Qva,vbne(L)={∑eva∈EvaCrossvb,eva(L)+∑evb∈EvbCrossva,evb(L)(=Qne)if (va,vb)∉EQne+∑v∈VCrossv,(va,vb)(L)if (va,vb)∈E     (23)
 MathType@MTEF@5@5@+=feaafiart1ev1aaatCvAUfKttLearuWrP9MDH5MBPbIqV92AaeXatLxBI9gBaebbnrfifHhDYfgasaacH8akY=wiFfYdH8Gipec8Eeeu0xXdbba9frFj0=OqFfea0dXdd9vqai=hGuQ8kuc9pgc9s8qqaq=dirpe0xb9q8qiLsFr0=vr0=vr0dc8meaabaqaciaacaGaaeqabaqabeGadaaakeaacqWGrbqudaqhaaWcbaGaemODay3aaSbaaWqaaiabdggaHbqabaWccqGGSaalcqWG2bGDdaWgaaadbaGaemOyaigabeaaaSqaaiabd6gaUjabdwgaLbaakiabcIcaOiabdYeamjabcMcaPiabg2da9maaceqabaqbaeaabiWaaaqaamaaqafabaGaem4qamKaemOCaiNaem4Ba8Maem4CamNaem4Cam3aaSbaaSqaaiabdAha2naaBaaameaacqWGIbGyaeqaaSGaeiilaWIaemyzau2aaSbaaWqaaiabdAha2naaBaaabaGaemyyaegabeaaaeqaaaWcbeaakiabcIcaOiabdYeamjabcMcaPaWcbaGaemyzau2aaSbaaWqaaiabdAha2naaBaaabaGaemyyaegabeaaaeqaaSGaeyicI4Saemyrau0aaSbaaWqaaiabdAha2naaBaaabaGaemyyaegabeaaaeqaaaWcbeqdcqGHris5aOGaey4kaSYaaabuaeaacqWGdbWqcqWGYbGCcqWGVbWBcqWGZbWCcqWGZbWCdaWgaaWcbaGaemODay3aaSbaaWqaaiabdggaHbqabaWccqGGSaalcqWGLbqzdaWgaaadbaGaemODay3aaSbaaeaacqWGIbGyaeqaaaqabaaaleqaaOGaeiikaGIaemitaWKaeiykaKcaleaacqWGLbqzdaWgaaadbaGaemODay3aaSbaaeaacqWGIbGyaeqaaaqabaWccqGHiiIZcqWGfbqrdaWgaaadbaGaemODay3aaSbaaeaacqWGIbGyaeqaaaqabaaaleqaniabggHiLdaakeaacqGGOaakcqGH9aqpcqWGrbqudaahaaWcbeqaaiabd6gaUjabdwgaLbaakiabcMcaPaqaaiabbMgaPjabbAgaMjabbccaGiabcIcaOiabdAha2naaBaaaleaacqWGHbqyaeqaaOGaeiilaWIaemODay3aaSbaaSqaaiabdkgaIbqabaGccqGGPaqkcqGHjiYZcqWGfbqraeaacqWGrbqudaahaaWcbeqaaiabd6gaUjabdwgaLbaakiabgUcaRmaaqafabaGaem4qamKaemOCaiNaem4Ba8Maem4CamNaem4Cam3aaSbaaSqaaiabdAha2jabcYcaSiabcIcaOiabdAha2naaBaaameaacqWGHbqyaeqaaSGaeiilaWIaemODay3aaSbaaWqaaiabdkgaIbqabaWccqGGPaqkaeqaaOGaeiikaGIaemitaWKaeiykaKcaleaacqWG2bGDcqGHiiIZcqWGwbGvaeqaniabggHiLdaakeaaaeaacqqGPbqAcqqGMbGzcqqGGaaicqGGOaakcqWG2bGDdaWgaaWcbaGaemyyaegabeaakiabcYcaSiabdAha2naaBaaaleaacqWGIbGyaeqaaOGaeiykaKIaeyicI4SaemyraueaaaGaay5EaaGaaCzcaiaaxMaadaqadaqaaiabikdaYiabiodaZaGaayjkaiaawMcaaaaa@C2CB@

Similarly, Q^va,vb,vcne
 MathType@MTEF@5@5@+=feaafiart1ev1aaatCvAUfKttLearuWrP9MDH5MBPbIqV92AaeXatLxBI9gBaebbnrfifHhDYfgasaacH8akY=wiFfYdH8Gipec8Eeeu0xXdbba9frFj0=OqFfea0dXdd9vqai=hGuQ8kuc9pgc9s8qqaq=dirpe0xb9q8qiLsFr0=vr0=vr0dc8meaabaqaciaacaGaaeqabaqabeGadaaakeaacuWGrbqugaqcamaaDaaaleaacqWG2bGDdaWgaaadbaGaemyyaegabeaaliabcYcaSiabdAha2naaBaaameaacqWGIbGyaeqaaSGaeiilaWIaemODay3aaSbaaWqaaiabdogaJbqabaaaleaacqWGUbGBcqWGLbqzaaaaaa@3B7A@ (·) is a partial node-edge crossing cost function of *v*_*a*_, *v*_*b*_, *v*_*c*_, Eva
 MathType@MTEF@5@5@+=feaafiart1ev1aaatCvAUfKttLearuWrP9MDH5MBPbIqV92AaeXatLxBI9gBaebbnrfifHhDYfgasaacH8akY=wiFfYdH8Gipec8Eeeu0xXdbba9frFj0=OqFfea0dXdd9vqai=hGuQ8kuc9pgc9s8qqaq=dirpe0xb9q8qiLsFr0=vr0=vr0dc8meaabaqaciaacaGaaeqabaqabeGadaaakeaacqWGfbqrdaWgaaWcbaGaemODay3aaSbaaWqaaiabdggaHbqabaaaleqaaaaa@30E3@, Evb
 MathType@MTEF@5@5@+=feaafiart1ev1aaatCvAUfKttLearuWrP9MDH5MBPbIqV92AaeXatLxBI9gBaebbnrfifHhDYfgasaacH8akY=wiFfYdH8Gipec8Eeeu0xXdbba9frFj0=OqFfea0dXdd9vqai=hGuQ8kuc9pgc9s8qqaq=dirpe0xb9q8qiLsFr0=vr0=vr0dc8meaabaqaciaacaGaaeqabaqabeGadaaakeaacqWGfbqrdaWgaaWcbaGaemODay3aaSbaaWqaaiabdkgaIbqabaaaleqaaaaa@30E5@, and Evc
 MathType@MTEF@5@5@+=feaafiart1ev1aaatCvAUfKttLearuWrP9MDH5MBPbIqV92AaeXatLxBI9gBaebbnrfifHhDYfgasaacH8akY=wiFfYdH8Gipec8Eeeu0xXdbba9frFj0=OqFfea0dXdd9vqai=hGuQ8kuc9pgc9s8qqaq=dirpe0xb9q8qiLsFr0=vr0=vr0dc8meaabaqaciaacaGaaeqabaqabeGadaaakeaacqWGfbqrdaWgaaWcbaGaemODay3aaSbaaWqaaiabdogaJbqabaaaleqaaaaa@30E7@, and is defined as follows:

Q^va,vb,vcne(L)={∑eva∈EvaCrossvb,eva(L)+∑eva∈EvaCrossvc,eva(L)+∑evb∈EvbCrossva,evb(L)+∑evc∈EvcCrossva,evc(L) (=Q^ne)if (va,vb)∉E,(va,vc)∉EQ^ne+∑v∈V\vcCrossv,(va,vb)(L)if (va,vb)∈E,(va,vc)∉EQ^ne+∑v∈V\vbCrossv,(va,vc)(L)if (va,vb)∉E,(va,vc)∈EQ^ne+∑v∈V\vcCrossv,(va,vb)(L)+∑v∈V\vbCrossv,(va,vc)(L)if (va,vb)∈E,(va,vc)∈E.     (24)
 MathType@MTEF@5@5@+=feaafiart1ev1aaatCvAUfKttLearuWrP9MDH5MBPbIqV92AaeXatLxBI9gBaebbnrfifHhDYfgasaacH8akY=wiFfYdH8Gipec8Eeeu0xXdbba9frFj0=OqFfea0dXdd9vqai=hGuQ8kuc9pgc9s8qqaq=dirpe0xb9q8qiLsFr0=vr0=vr0dc8meaabaqaciaacaGaaeqabaqabeGadaaakeaacuWGrbqugaqcamaaDaaaleaacqWG2bGDdaWgaaadbaGaemyyaegabeaaliabcYcaSiabdAha2naaBaaameaacqWGIbGyaeqaaSGaeiilaWIaemODay3aaSbaaWqaaiabdogaJbqabaaaleaacqWGUbGBcqWGLbqzaaGccqGGOaakcqWGmbatcqGGPaqkcqGH9aqpdaGabeqaauaabaqaeiaaaaqaauaabeqaceaaaeaadaaeqbqaaiabdoeadjabdkhaYjabd+gaVjabdohaZjabdohaZnaaBaaaleaacqWG2bGDdaWgaaadbaGaemOyaigabeaaliabcYcaSiabdwgaLnaaBaaameaacqWG2bGDdaWgaaqaaiabdggaHbqabaaabeaaaSqabaGccqGGOaakcqWGmbatcqGGPaqkaSqaaiabdwgaLnaaBaaameaacqWG2bGDdaWgaaqaaiabdggaHbqabaaabeaaliabgIGiolabdweafnaaBaaameaacqWG2bGDdaWgaaqaaiabdggaHbqabaaabeaaaSqab0GaeyyeIuoakiabgUcaRmaaqafabaGaem4qamKaemOCaiNaem4Ba8Maem4CamNaem4Cam3aaSbaaSqaaiabdAha2naaBaaameaacqWGJbWyaeqaaSGaeiilaWIaemyzau2aaSbaaWqaaiabdAha2naaBaaabaGaemyyaegabeaaaeqaaaWcbeaakiabcIcaOiabdYeamjabcMcaPaWcbaGaemyzau2aaSbaaWqaaiabdAha2naaBaaabaGaemyyaegabeaaaeqaaSGaeyicI4Saemyrau0aaSbaaWqaaiabdAha2naaBaaabaGaemyyaegabeaaaeqaaaWcbeqdcqGHris5aaGcbaGaey4kaSYaaabuaeaacqWGdbWqcqWGYbGCcqWGVbWBcqWGZbWCcqWGZbWCdaWgaaWcbaGaemODay3aaSbaaWqaaiabdggaHbqabaWccqGGSaalcqWGLbqzdaWgaaadbaGaemODay3aaSbaaeaacqWGIbGyaeqaaaqabaaaleqaaOGaeiikaGIaemitaWKaeiykaKcaleaacqWGLbqzdaWgaaadbaGaemODay3aaSbaaeaacqWGIbGyaeqaaaqabaWccqGHiiIZcqWGfbqrdaWgaaadbaGaemODay3aaSbaaeaacqWGIbGyaeqaaaqabaaaleqaniabggHiLdGccqGHRaWkdaaeqbqaaiabdoeadjabdkhaYjabd+gaVjabdohaZjabdohaZnaaBaaaleaacqWG2bGDdaWgaaadbaGaemyyaegabeaaliabcYcaSiabdwgaLnaaBaaameaacqWG2bGDdaWgaaqaaiabdogaJbqabaaabeaaaSqabaGccqGGOaakcqWGmbatcqGGPaqkaSqaaiabdwgaLnaaBaaameaacqWG2bGDdaWgaaqaaiabdogaJbqabaaabeaaliabgIGiolabdweafnaaBaaameaacqWG2bGDdaWgaaqaaiabdogaJbqabaaabeaaaSqab0GaeyyeIuoaaaGccqqGGaaicqGGOaakcqGH9aqpcuWGrbqugaqcamaaCaaaleqabaGaemOBa4MaemyzaugaaOGaeiykaKcabaGaeeyAaKMaeeOzayMaeeiiaasbaeqabiqaaaqaaiabcIcaOiabdAha2naaBaaaleaacqWGHbqyaeqaaOGaeiilaWIaemODay3aaSbaaSqaaiabdkgaIbqabaGccqGGPaqkcqGHjiYZcqWGfbqrcqGGSaalaeaacqGGOaakcqWG2bGDdaWgaaWcbaGaemyyaegabeaakiabcYcaSiabdAha2naaBaaaleaacqWGJbWyaeqaaOGaeiykaKIaeyycI8SaemyraueaaaqaaiqbdgfarzaajaWaaWbaaSqabeaacqWGUbGBcqWGLbqzaaGccqGHRaWkdaaeqbqaaiabdoeadjabdkhaYjabd+gaVjabdohaZjabdohaZnaaBaaaleaacqWG2bGDcqGGSaalcqGGOaakcqWG2bGDdaWgaaadbaGaemyyaegabeaaliabcYcaSiabdAha2naaBaaameaacqWGIbGyaeqaaSGaeiykaKcabeaakiabcIcaOiabdYeamjabcMcaPaWcbaGaemODayNaeyicI4SaemOvayLaeiixaWLaemODay3aaSbaaWqaaiabdogaJbqabaaaleqaniabggHiLdaakeaacqqGPbqAcqqGMbGzcqqGGaaifaqabeGabaaabaGaeiikaGIaemODay3aaSbaaSqaaiabdggaHbqabaGccqGGSaalcqWG2bGDdaWgaaWcbaGaemOyaigabeaakiabcMcaPiabgIGiolabdweafjabcYcaSaqaaiabcIcaOiabdAha2naaBaaaleaacqWGHbqyaeqaaOGaeiilaWIaemODay3aaSbaaSqaaiabdogaJbqabaGccqGGPaqkcqGHjiYZcqWGfbqraaaabaGafmyuaeLbaKaadaahaaWcbeqaaiabd6gaUjabdwgaLbaakiabgUcaRmaaqafabaGaem4qamKaemOCaiNaem4Ba8Maem4CamNaem4Cam3aaSbaaSqaaiabdAha2jabcYcaSiabcIcaOiabdAha2naaBaaameaacqWGHbqyaeqaaSGaeiilaWIaemODay3aaSbaaWqaaiabdogaJbqabaWccqGGPaqkaeqaaOGaeiikaGIaemitaWKaeiykaKcaleaacqWG2bGDcqGHiiIZcqWGwbGvcqGGCbaxcqWG2bGDdaWgaaadbaGaemOyaigabeaaaSqab0GaeyyeIuoaaOqaaiabbMgaPjabbAgaMjabbccaGuaabeqaceaaaeaacqGGOaakcqWG2bGDdaWgaaWcbaGaemyyaegabeaakiabcYcaSiabdAha2naaBaaaleaacqWGIbGyaeqaaOGaeiykaKIaeyycI8SaemyrauKaeiilaWcabaGaeiikaGIaemODay3aaSbaaSqaaiabdggaHbqabaGccqGGSaalcqWG2bGDdaWgaaWcbaGaem4yamgabeaakiabcMcaPiabgIGiolabdweafbaaaeaacuWGrbqugaqcamaaCaaaleqabaGaemOBa4MaemyzaugaaOGaey4kaSYaaabuaeaacqWGdbWqcqWGYbGCcqWGVbWBcqWGZbWCcqWGZbWCdaWgaaWcbaGaemODayNaeiilaWIaeiikaGIaemODay3aaSbaaWqaaiabdggaHbqabaWccqGGSaalcqWG2bGDdaWgaaadbaGaemOyaigabeaaliabcMcaPaqabaGccqGGOaakcqWGmbatcqGGPaqkaSqaaiabdAha2jabgIGiolabdAfawjabcYfaCjabdAha2naaBaaameaacqWGJbWyaeqaaaWcbeqdcqGHris5aOGaey4kaSYaaabuaeaacqWGdbWqcqWGYbGCcqWGVbWBcqWGZbWCcqWGZbWCdaWgaaWcbaGaemODayNaeiilaWIaeiikaGIaemODay3aaSbaaWqaaiabdggaHbqabaWccqGGSaalcqWG2bGDdaWgaaadbaGaem4yamgabeaaliabcMcaPaqabaGccqGGOaakcqWGmbatcqGGPaqkaSqaaiabdAha2jabgIGiolabdAfawjabcYfaCjabdAha2naaBaaameaacqWGIbGyaeqaaaWcbeqdcqGHris5aaGcbaGaeeyAaKMaeeOzayMaeeiiaasbaeqabiqaaaqaaiabcIcaOiabdAha2naaBaaaleaacqWGHbqyaeqaaOGaeiilaWIaemODay3aaSbaaSqaaiabdkgaIbqabaGccqGGPaqkcqGHiiIZcqWGfbqrcqGGSaalaeaacqGGOaakcqWG2bGDdaWgaaWcbaGaemyyaegabeaakiabcYcaSiabdAha2naaBaaaleaacqWGJbWyaeqaaOGaeiykaKIaeyicI4SaemyraueaaaaaaiaawUhaaiabc6caUiaaxMaacaWLjaWaaeWaaeaacqaIYaGmcqaI0aanaiaawIcacaGLPaaaaaa@C12C@

(c) Qva,vbdc
 MathType@MTEF@5@5@+=feaafiart1ev1aaatCvAUfKttLearuWrP9MDH5MBPbIqV92AaeXatLxBI9gBaebbnrfifHhDYfgasaacH8akY=wiFfYdH8Gipec8Eeeu0xXdbba9frFj0=OqFfea0dXdd9vqai=hGuQ8kuc9pgc9s8qqaq=dirpe0xb9q8qiLsFr0=vr0=vr0dc8meaabaqaciaacaGaaeqabaqabeGadaaakeaacqWGrbqudaqhaaWcbaGaemODay3aaSbaaWqaaiabdggaHbqabaWccqGGSaalcqWG2bGDdaWgaaadbaGaemOyaigabeaaaSqaaiabdsgaKjabdogaJbaaaaa@3776@ is a partial distance cost function of *v*_*a *_and *v*_*b*_, and is defined as follows:

Qva,vbdc(L)=Distanceva,vb(L).     (25)
 MathType@MTEF@5@5@+=feaafiart1ev1aaatCvAUfKttLearuWrP9MDH5MBPbIqV92AaeXatLxBI9gBaebbnrfifHhDYfgasaacH8akY=wiFfYdH8Gipec8Eeeu0xXdbba9frFj0=OqFfea0dXdd9vqai=hGuQ8kuc9pgc9s8qqaq=dirpe0xb9q8qiLsFr0=vr0=vr0dc8meaabaqaciaacaGaaeqabaqabeGadaaakeaacqWGrbqudaqhaaWcbaGaemODay3aaSbaaWqaaiabdggaHbqabaWccqGGSaalcqWG2bGDdaWgaaadbaGaemOyaigabeaaaSqaaiabdsgaKjabdogaJbaakiabcIcaOiabdYeamjabcMcaPiabg2da9iabdseaejabdMgaPjabdohaZHqaciab=rha0jab=fgaHjab=5gaUjabdogaJjabdwgaLnaaBaaaleaacqWG2bGDdaWgaaadbaGaemyyaegabeaaliabcYcaSiabdAha2naaBaaameaacqWGIbGyaeqaaaWcbeaakiabcIcaOiabdYeamjabcMcaPiabc6caUiaaxMaacaWLjaWaaeWaaeaacqaIYaGmcqaI1aqnaiaawIcacaGLPaaaaaa@556C@

Similarly, Q^va,vb,vcdc
 MathType@MTEF@5@5@+=feaafiart1ev1aaatCvAUfKttLearuWrP9MDH5MBPbIqV92AaeXatLxBI9gBaebbnrfifHhDYfgasaacH8akY=wiFfYdH8Gipec8Eeeu0xXdbba9frFj0=OqFfea0dXdd9vqai=hGuQ8kuc9pgc9s8qqaq=dirpe0xb9q8qiLsFr0=vr0=vr0dc8meaabaqaciaacaGaaeqabaqabeGadaaakeaacuWGrbqugaqcamaaDaaaleaacqWG2bGDdaWgaaadbaGaemyyaegabeaaliabcYcaSiabdAha2naaBaaameaacqWGIbGyaeqaaSGaeiilaWIaemODay3aaSbaaWqaaiabdogaJbqabaaaleaacqWGKbazcqWGJbWyaaaaaa@3B62@ (·) is a partial distance cost function of *v*_*a*_, *v*_*b*_, and *v*_*c*_, and is defined as follows:

Q^va,vb,vcdc(L)=Distanceva,vb(L)+Distanceva,vc(L).     (26)
 MathType@MTEF@5@5@+=feaafiart1ev1aaatCvAUfKttLearuWrP9MDH5MBPbIqV92AaeXatLxBI9gBaebbnrfifHhDYfgasaacH8akY=wiFfYdH8Gipec8Eeeu0xXdbba9frFj0=OqFfea0dXdd9vqai=hGuQ8kuc9pgc9s8qqaq=dirpe0xb9q8qiLsFr0=vr0=vr0dc8meaabaqaciaacaGaaeqabaqabeGadaaakeaacuWGrbqugaqcamaaDaaaleaacqWG2bGDdaWgaaadbaGaemyyaegabeaaliabcYcaSiabdAha2naaBaaameaacqWGIbGyaeqaaSGaeiilaWIaemODay3aaSbaaWqaaiabdogaJbqabaaaleaacqWGKbazcqWGJbWyaaGccqGGOaakcqWGmbatcqGGPaqkcqGH9aqpcqWGebarcqWGPbqAcqWGZbWCieGacqWF0baDcqWFHbqycqWFUbGBcqWGJbWycqWGLbqzdaWgaaWcbaGaemODay3aaSbaaWqaaiabdggaHbqabaWccqGGSaalcqWG2bGDdaWgaaadbaGaemOyaigabeaaaSqabaGccqGGOaakcqWGmbatcqGGPaqkcqGHRaWkcqWGebarcqWGPbqAcqWGZbWCcqWF0baDcqWFHbqycqWFUbGBcqWGJbWycqWGLbqzdaWgaaWcbaGaemODay3aaSbaaWqaaiabdggaHbqabaWccqGGSaalcqWG2bGDdaWgaaadbaGaem4yamgabeaaaSqabaGccqGGOaakcqWGmbatcqGGPaqkcqGGUaGlcaWLjaGaaCzcamaabmaabaGaeGOmaiJaeGOnaydacaGLOaGaayzkaaaaaa@6EAB@

Thus far, we found out a method to efficiently calculate Δ matrix. The purpose of extending Δ matrix is to calculate the cost difference of the swap operation. When nodes vα1
 MathType@MTEF@5@5@+=feaafiart1ev1aaatCvAUfKttLearuWrP9MDH5MBPbIqV92AaeXatLxBI9gBaebbnrfifHhDYfgasaacH8akY=wiFfYdH8Gipec8Eeeu0xXdbba9frFj0=OqFfea0dXdd9vqai=hGuQ8kuc9pgc9s8qqaq=dirpe0xb9q8qiLsFr0=vr0=vr0dc8meaabaqaciaacaGaaeqabaqabeGadaaakeaacqWG2bGDdaWgaaWcbaacciGae8xSde2aaSbaaWqaaiabigdaXaqabaaaleqaaaaa@311B@ and vα2
 MathType@MTEF@5@5@+=feaafiart1ev1aaatCvAUfKttLearuWrP9MDH5MBPbIqV92AaeXatLxBI9gBaebbnrfifHhDYfgasaacH8akY=wiFfYdH8Gipec8Eeeu0xXdbba9frFj0=OqFfea0dXdd9vqai=hGuQ8kuc9pgc9s8qqaq=dirpe0xb9q8qiLsFr0=vr0=vr0dc8meaabaqaciaacaGaaeqabaqabeGadaaakeaacqWG2bGDdaWgaaWcbaacciGae8xSde2aaSbaaWqaaiabikdaYaqabaaaleqaaaaa@311D@ are swapped, we can calculate Swapvα1,vα2
 MathType@MTEF@5@5@+=feaafiart1ev1aaatCvAUfKttLearuWrP9MDH5MBPbIqV92AaeXatLxBI9gBaebbnrfifHhDYfgasaacH8akY=wiFfYdH8Gipec8Eeeu0xXdbba9frFj0=OqFfea0dXdd9vqai=hGuQ8kuc9pgc9s8qqaq=dirpe0xb9q8qiLsFr0=vr0=vr0dc8meaabaqaciaacaGaaeqabaqabeGadaaakeaacqWGtbWucqWG3bWDcqWGHbqycqWGWbaCdaWgaaWcbaGaemODay3aaSbaaWqaaGGaciab=f7aHnaaBaaabaGaeGymaedabeaaaeqaaSGaeiilaWIaemODay3aaSbaaWqaaiab=f7aHnaaBaaabaGaeGOmaidabeaaaeqaaaWcbeaaaaa@3BD0@ using these Δ costs as follows:

Swapvα1vα2(Svα1↔vα2L)=Δvα1P(vα2)(L)+Δvα2P(vα1)(L)+Rvα1,vα2(Svα1↔vα2L)−Rvα1,vα2(L),     (27)
 MathType@MTEF@5@5@+=feaafiart1ev1aaatCvAUfKttLearuWrP9MDH5MBPbIqV92AaeXatLxBI9gBaebbnrfifHhDYfgasaacH8akY=wiFfYdH8Gipec8Eeeu0xXdbba9frFj0=OqFfea0dXdd9vqai=hGuQ8kuc9pgc9s8qqaq=dirpe0xb9q8qiLsFr0=vr0=vr0dc8meaabaqaciaacaGaaeqabaqabeGadaaakeaacqWGtbWucqWG3bWDcqWGHbqycqWGWbaCdaWgaaWcbaGaemODay3aaSbaaWqaaGGaciab=f7aHnaaBaaabaGaeGymaedabeaaaeqaaSGaemODay3aaSbaaWqaaiab=f7aHnaaBaaabaGaeGOmaidabeaaaeqaaaWcbeaakiabcIcaOiabdofatnaaBaaaleaacqWG2bGDdaWgaaadbaGae8xSde2aaSbaaeaacqaIXaqmaeqaaaqabaWccqGHugYQcqWG2bGDdaWgaaadbaGae8xSde2aaSbaaeaacqaIYaGmaeqaaaqabaaaleqaaOGaemitaWKaeiykaKIaeyypa0JaeuiLdq0aaSbaaSqaaiabdAha2naaBaaameaacqWFXoqydaWgaaqaaiabigdaXaqabaaabeaaliabdcfaqjabcIcaOiabdAha2naaBaaameaacqWFXoqydaWgaaqaaiabikdaYaqabaaabeaaliabcMcaPaqabaGccqGGOaakcqWGmbatcqGGPaqkcqGHRaWkcqqHuoardaWgaaWcbaGaemODay3aaSbaaWqaaiab=f7aHnaaBaaabaGaeGOmaidabeaaaeqaaSGaemiuaaLaeiikaGIaemODay3aaSbaaWqaaiab=f7aHnaaBaaabaGaeGymaedabeaaaeqaaSGaeiykaKcabeaakiabcIcaOiabdYeamjabcMcaPiabgUcaRiabdkfasnaaBaaaleaacqWG2bGDdaWgaaadbaGae8xSde2aaSbaaeaacqaIXaqmaeqaaaqabaWccqGGSaalcqWG2bGDdaWgaaadbaGae8xSde2aaSbaaeaacqaIYaGmaeqaaaqabaaaleqaaOGaeiikaGIaem4uam1aaSbaaSqaaiabdAha2naaBaaameaacqWFXoqydaWgaaqaaiabigdaXaqabaaabeaaliabgsziRkabdAha2naaBaaameaacqWFXoqydaWgaaqaaiabikdaYaqabaaabeaaaSqabaGccqWGmbatcqGGPaqkcqGHsislcqWGsbGudaWgaaWcbaGaemODay3aaSbaaWqaaiab=f7aHnaaBaaabaGaeGymaedabeaaaeqaaSGaeiilaWIaemODay3aaSbaaWqaaiab=f7aHnaaBaaabaGaeGOmaidabeaaaeqaaaWcbeaakiabcIcaOiabdYeamjabcMcaPiabcYcaSiaaxMaacaWLjaWaaeWaaeaacqaIYaGmcqaI3aWnaiaawIcacaGLPaaaaaa@9AAD@

where

Rva,vb(L)=Wee∑eva∈Eva,evb∈EvbCrosseva,evb(L)+Wve(∑eva∈EvaCrossvb,eva(L)+∑evb∈EvbCrossva,evb(L)).     (28)
 MathType@MTEF@5@5@+=feaafiart1ev1aaatCvAUfKttLearuWrP9MDH5MBPbIqV92AaeXatLxBI9gBaebbnrfifHhDYfgasaacH8akY=wiFfYdH8Gipec8Eeeu0xXdbba9frFj0=OqFfea0dXdd9vqai=hGuQ8kuc9pgc9s8qqaq=dirpe0xb9q8qiLsFr0=vr0=vr0dc8meaabaqaciaacaGaaeqabaqabeGadaaakeaacqWGsbGudaWgaaWcbaGaemODay3aaSbaaWqaaiabdggaHbqabaWccqGGSaalcqWG2bGDdaWgaaadbaGaemOyaigabeaaaSqabaGccqGGOaakcqWGmbatcqGGPaqkcqGH9aqpcqWGxbWvdaWgaaWcbaGaemyzauMaemyzaugabeaakmaaqafabaGaem4qamKaemOCaiNaem4Ba8Maem4CamNaem4Cam3aaSbaaSqaaiabdwgaLnaaBaaameaacqWG2bGDdaWgaaqaaiabdggaHbqabaaabeaaliabcYcaSiabdwgaLnaaBaaameaacqWG2bGDdaWgaaqaaiabdkgaIbqabaaabeaaaSqabaGccqGGOaakcqWGmbatcqGGPaqkcqGHRaWkcqWGxbWvdaWgaaWcbaGaemODayNaemyzaugabeaaaeaacqWGLbqzdaWgaaadbaGaemODay3aaSbaaeaacqWGHbqyaeqaaaqabaWccqGHiiIZcqWGfbqrdaWgaaadbaGaemODay3aaSbaaeaacqWGHbqyaeqaaaqabaWccqGGSaalcqWGLbqzdaWgaaadbaGaemODay3aaSbaaeaacqWGIbGyaeqaaaqabaWccqGHiiIZcqWGfbqrdaWgaaadbaGaemODay3aaSbaaeaacqWGIbGyaeqaaaqabaaaleqaniabggHiLdGcdaqadaqaamaaqafabaGaem4qamKaemOCaiNaem4Ba8Maem4CamNaem4Cam3aaSbaaSqaaiabdAha2naaBaaameaacqWGIbGyaeqaaSGaeiilaWIaemyzau2aaSbaaWqaaiabdAha2naaBaaabaGaemyyaegabeaaaeqaaaWcbeaakiabcIcaOiabdYeamjabcMcaPaWcbaGaemyzau2aaSbaaWqaaiabdAha2naaBaaabaGaemyyaegabeaaaeqaaSGaeyicI4Saemyrau0aaSbaaWqaaiabdAha2naaBaaabaGaemyyaegabeaaaeqaaaWcbeqdcqGHris5aOGaey4kaSYaaabuaeaacqWGdbWqcqWGYbGCcqWGVbWBcqWGZbWCcqWGZbWCdaWgaaWcbaGaemODay3aaSbaaWqaaiabdggaHbqabaWccqGGSaalcqWGLbqzdaWgaaadbaGaemODay3aaSbaaeaacqWGIbGyaeqaaaqabaaaleqaaOGaeiikaGIaemitaWKaeiykaKcaleaacqWGLbqzdaWgaaadbaGaemODay3aaSbaaeaacqWGIbGyaeqaaaqabaWccqGHiiIZcqWGfbqrdaWgaaadbaGaemODay3aaSbaaeaacqWGIbGyaeqaaaqabaaaleqaniabggHiLdaakiaawIcacaGLPaaacqGGUaGlcaWLjaGaaCzcamaabmaabaGaeGOmaiJaeGioaGdacaGLOaGaayzkaaaaaa@B15E@

In SCCB-grid layout algorithm, the combo score also needs to be considered. Given a layout such that a node *v*_*α *_is moved to a vacant point *p*, Δvαpcs
 MathType@MTEF@5@5@+=feaafiart1ev1aaatCvAUfKttLearuWrP9MDH5MBPbIqV92AaeXatLxBI9gBaebbnrfifHhDYfgasaacH8akY=wiFfYdH8Gipec8Eeeu0xXdbba9frFj0=OqFfea0dXdd9vqai=hGuQ8kuc9pgc9s8qqaq=dirpe0xb9q8qiLsFr0=vr0=vr0dc8meaabaqaciaacaGaaeqabaqabeGadaaakeaacqqHuoardaqhaaWcbaGaemODay3aaSbaaWqaaGGaciab=f7aHbqabaWccqWGWbaCaeaacqWGJbWycqWGZbWCaaaaaa@35B9@ can be calculated as shown in Equation (3). In contrast, if two nodes vα1
 MathType@MTEF@5@5@+=feaafiart1ev1aaatCvAUfKttLearuWrP9MDH5MBPbIqV92AaeXatLxBI9gBaebbnrfifHhDYfgasaacH8akY=wiFfYdH8Gipec8Eeeu0xXdbba9frFj0=OqFfea0dXdd9vqai=hGuQ8kuc9pgc9s8qqaq=dirpe0xb9q8qiLsFr0=vr0=vr0dc8meaabaqaciaacaGaaeqabaqabeGadaaakeaacqWG2bGDdaWgaaWcbaacciGae8xSde2aaSbaaWqaaiabigdaXaqabaaaleqaaaaa@311B@ and vα2
 MathType@MTEF@5@5@+=feaafiart1ev1aaatCvAUfKttLearuWrP9MDH5MBPbIqV92AaeXatLxBI9gBaebbnrfifHhDYfgasaacH8akY=wiFfYdH8Gipec8Eeeu0xXdbba9frFj0=OqFfea0dXdd9vqai=hGuQ8kuc9pgc9s8qqaq=dirpe0xb9q8qiLsFr0=vr0=vr0dc8meaabaqaciaacaGaaeqabaqabeGadaaakeaacqWG2bGDdaWgaaWcbaacciGae8xSde2aaSbaaWqaaiabikdaYaqabaaaleqaaaaa@311D@ are swapped, the difference of combo scores, *Combo *(Svα1↔vα2
 MathType@MTEF@5@5@+=feaafiart1ev1aaatCvAUfKttLearuWrP9MDH5MBPbIqV92AaeXatLxBI9gBaebbnrfifHhDYfgasaacH8akY=wiFfYdH8Gipec8Eeeu0xXdbba9frFj0=OqFfea0dXdd9vqai=hGuQ8kuc9pgc9s8qqaq=dirpe0xb9q8qiLsFr0=vr0=vr0dc8meaabaqaciaacaGaaeqabaqabeGadaaakeaacqWGtbWudaWgaaWcbaGaemODay3aaSbaaWqaaGGaciab=f7aHnaaBaaabaGaeGymaedabeaaaeqaaSGaeyiLHSQaemODay3aaSbaaWqaaiab=f7aHnaaBaaabaGaeGOmaidabeaaaeqaaaWcbeaaaaa@38B1@*L*) – *Combo *(*L*), is effectively calculated as follows:

Swapvα1,vα2cs(L)=pSwapvα1vα2(L)+pSwapvα2vα1(L),     (29)
 MathType@MTEF@5@5@+=feaafiart1ev1aaatCvAUfKttLearuWrP9MDH5MBPbIqV92AaeXatLxBI9gBaebbnrfifHhDYfgasaacH8akY=wiFfYdH8Gipec8Eeeu0xXdbba9frFj0=OqFfea0dXdd9vqai=hGuQ8kuc9pgc9s8qqaq=dirpe0xb9q8qiLsFr0=vr0=vr0dc8meaabaqaciaacaGaaeqabaqabeGadaaakeaacqWGtbWucqWG3bWDcqWGHbqycqWGWbaCdaqhaaWcbaGaemODay3aaSbaaWqaaGGaciab=f7aHnaaBaaabaGaeGymaedabeaaaeqaaSGaeiilaWIaemODay3aaSbaaWqaaiab=f7aHnaaBaaabaGaeGOmaidabeaaaeqaaaWcbaGaem4yamMaem4CamhaaOGaeiikaGIaemitaWKaeiykaKIaeyypa0JaemiCaaNaem4uamLaem4DaCNaemyyaeMaemiCaa3aaSbaaSqaaiabdAha2naaBaaameaacqWFXoqydaWgaaqaaiabigdaXaqabaaabeaaliabdAha2naaBaaameaacqWFXoqydaWgaaqaaiabikdaYaqabaaabeaaaSqabaGccqGGOaakcqWGmbatcqGGPaqkcqGHRaWkcqWGWbaCcqWGtbWucqWG3bWDcqWGHbqycqWGWbaCdaWgaaWcbaGaemODay3aaSbaaWqaaiab=f7aHnaaBaaabaGaeGOmaidabeaaaeqaaSGaemODay3aaSbaaWqaaiab=f7aHnaaBaaabaGaeGymaedabeaaaeqaaaWcbeaakiabcIcaOiabdYeamjabcMcaPiabcYcaSiaaxMaacaWLjaWaaeWaaeaacqaIYaGmcqaI5aqoaiaawIcacaGLPaaaaaa@6DEF@

where

pSwapvu(L)={Wcs(Combov(Sv↔uL)−Combov(L)+Adjv(Sv↔uL)−Adjv(L)if v∈V′0if v∉V′.     (30)
 MathType@MTEF@5@5@+=feaafiart1ev1aaatCvAUfKttLearuWrP9MDH5MBPbIqV92AaeXatLxBI9gBaebbnrfifHhDYfgasaacH8akY=wiFfYdH8Gipec8Eeeu0xXdbba9frFj0=OqFfea0dXdd9vqai=hGuQ8kuc9pgc9s8qqaq=dirpe0xb9q8qiLsFr0=vr0=vr0dc8meaabaqaciaacaGaaeqabaqabeGadaaakeaacqWGWbaCcqWGtbWucqWG3bWDcqWGHbqycqWGWbaCdaWgaaWcbaGaemODayNaemyDauhabeaakiabcIcaOiabdYeamjabcMcaPiabg2da9maaceqabaqbaeqabiGaaaqaaiabdEfaxnaaBaaaleaacqWGJbWycqWGZbWCaeqaaOGaeiikaGIaem4qamKaem4Ba8MaemyBa0MaemOyaiMaem4Ba82aaSbaaSqaaiabdAha2bqabaGccqGGOaakcqWGtbWudaWgaaWcbaGaemODayNaeyiLHSQaemyDauhabeaakiabdYeamjabcMcaPiabgkHiTiabdoeadjabd+gaVjabd2gaTjabdkgaIjabd+gaVnaaBaaaleaacqWG2bGDaeqaaOGaeiikaGIaemitaWKaeiykaKIaey4kaSIaemyqaeKaemizaqMaemOAaO2aaSbaaSqaaiabdAha2bqabaGccqGGOaakcqWGtbWudaWgaaWcbaGaemODayNaeyiLHSQaemyDauhabeaakiabdYeamjabcMcaPiabgkHiTiabdgeabjabdsgaKjabdQgaQnaaBaaaleaacqWG2bGDaeqaaOGaeiikaGIaemitaWKaeiykaKcabaGaeeyAaKMaeeOzayMaeeiiaaIaemODayNaeyicI4SafmOvayLbauaaaeaacqaIWaamaeaacqqGPbqAcqqGMbGzcqqGGaaicqWG2bGDcqGHjiYZcuWGwbGvgaqbaaaaaiaawUhaaiabc6caUiaaxMaacaWLjaWaaeWaaeaacqaIZaWmcqaIWaamaiaawIcacaGLPaaaaaa@8C23@

**Figure 5:**
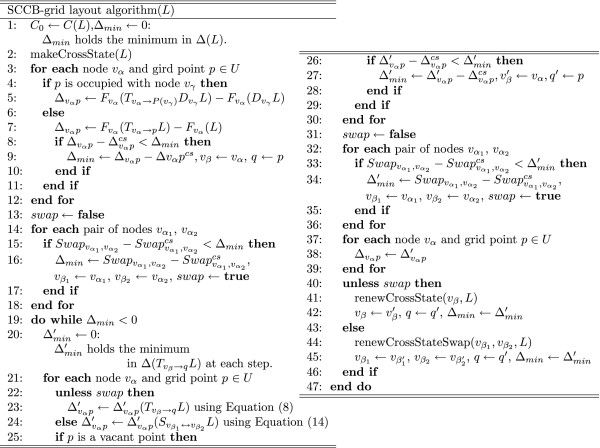
**SCCB-grid layout algorithm**. A pseudo code of SCCB-grid layout algorithm.

A pseudo code of SCCB-grid layout algorithm is described in Figure 5.

If node *v*_*β *_is moved at the previous step, the time complexity of calculating Δ matrix is *O *((|*V*| + |*E*|)|Evβ
 MathType@MTEF@5@5@+=feaafiart1ev1aaatCvAUfKttLearuWrP9MDH5MBPbIqV92AaeXatLxBI9gBaebbnrfifHhDYfgasaacH8akY=wiFfYdH8Gipec8Eeeu0xXdbba9frFj0=OqFfea0dXdd9vqai=hGuQ8kuc9pgc9s8qqaq=dirpe0xb9q8qiLsFr0=vr0=vr0dc8meaabaqaciaacaGaaeqabaqabeGadaaakeaacqWGfbqrdaWgaaWcbaGaemODay3aaSbaaWqaaGGaciab=j7aIbqabaaaleqaaaaa@3140@||*U*|). If two vβ1
 MathType@MTEF@5@5@+=feaafiart1ev1aaatCvAUfKttLearuWrP9MDH5MBPbIqV92AaeXatLxBI9gBaebbnrfifHhDYfgasaacH8akY=wiFfYdH8Gipec8Eeeu0xXdbba9frFj0=OqFfea0dXdd9vqai=hGuQ8kuc9pgc9s8qqaq=dirpe0xb9q8qiLsFr0=vr0=vr0dc8meaabaqaciaacaGaaeqabaqabeGadaaakeaacqWG2bGDdaWgaaWcbaacciGae8NSdi2aaSbaaWqaaiabigdaXaqabaaaleqaaaaa@311D@ and vβ2
 MathType@MTEF@5@5@+=feaafiart1ev1aaatCvAUfKttLearuWrP9MDH5MBPbIqV92AaeXatLxBI9gBaebbnrfifHhDYfgasaacH8akY=wiFfYdH8Gipec8Eeeu0xXdbba9frFj0=OqFfea0dXdd9vqai=hGuQ8kuc9pgc9s8qqaq=dirpe0xb9q8qiLsFr0=vr0=vr0dc8meaabaqaciaacaGaaeqabaqabeGadaaakeaacqWG2bGDdaWgaaWcbaacciGae8NSdi2aaSbaaWqaaiabikdaYaqabaaaleqaaaaa@311F@ are swapped at the previous step, the time complexity of calculating Δ matrix was *O *((|*V*| + |*E*|) (|Evβ1
 MathType@MTEF@5@5@+=feaafiart1ev1aaatCvAUfKttLearuWrP9MDH5MBPbIqV92AaeXatLxBI9gBaebbnrfifHhDYfgasaacH8akY=wiFfYdH8Gipec8Eeeu0xXdbba9frFj0=OqFfea0dXdd9vqai=hGuQ8kuc9pgc9s8qqaq=dirpe0xb9q8qiLsFr0=vr0=vr0dc8meaabaqaciaacaGaaeqabaqabeGadaaakeaacqWGfbqrdaWgaaWcbaGaemODay3aaSbaaWqaaGGaciab=j7aInaaBaaabaGaeGymaedabeaaaeqaaaWcbeaaaaa@3251@| + |Evβ2
 MathType@MTEF@5@5@+=feaafiart1ev1aaatCvAUfKttLearuWrP9MDH5MBPbIqV92AaeXatLxBI9gBaebbnrfifHhDYfgasaacH8akY=wiFfYdH8Gipec8Eeeu0xXdbba9frFj0=OqFfea0dXdd9vqai=hGuQ8kuc9pgc9s8qqaq=dirpe0xb9q8qiLsFr0=vr0=vr0dc8meaabaqaciaacaGaaeqabaqabeGadaaakeaacqWGfbqrdaWgaaWcbaGaemODay3aaSbaaWqaaGGaciab=j7aInaaBaaabaGaeGOmaidabeaaaeqaaaWcbeaaaaa@3253@|) |*U*|) = *O *((|*V*| + |*E*|) |Evβ′
 MathType@MTEF@5@5@+=feaafiart1ev1aaatCvAUfKttLearuWrP9MDH5MBPbIqV92AaeXatLxBI9gBaebbnrfifHhDYfgasaacH8akY=wiFfYdH8Gipec8Eeeu0xXdbba9frFj0=OqFfea0dXdd9vqai=hGuQ8kuc9pgc9s8qqaq=dirpe0xb9q8qiLsFr0=vr0=vr0dc8meaabaqaciaacaGaaeqabaqabeGadaaakeaacqWGfbqrdaWgaaWcbaGaemODay3aaSbaaWqaaGGaciqb=j7aIzaafaaabeaaaSqabaaaaa@314C@||*U*|), where |Evβ′
 MathType@MTEF@5@5@+=feaafiart1ev1aaatCvAUfKttLearuWrP9MDH5MBPbIqV92AaeXatLxBI9gBaebbnrfifHhDYfgasaacH8akY=wiFfYdH8Gipec8Eeeu0xXdbba9frFj0=OqFfea0dXdd9vqai=hGuQ8kuc9pgc9s8qqaq=dirpe0xb9q8qiLsFr0=vr0=vr0dc8meaabaqaciaacaGaaeqabaqabeGadaaakeaacqWGfbqrdaWgaaWcbaGaemODay3aaSbaaWqaaGGaciqb=j7aIzaafaaabeaaaSqabaaaaa@314C@| = (|Evβ1
 MathType@MTEF@5@5@+=feaafiart1ev1aaatCvAUfKttLearuWrP9MDH5MBPbIqV92AaeXatLxBI9gBaebbnrfifHhDYfgasaacH8akY=wiFfYdH8Gipec8Eeeu0xXdbba9frFj0=OqFfea0dXdd9vqai=hGuQ8kuc9pgc9s8qqaq=dirpe0xb9q8qiLsFr0=vr0=vr0dc8meaabaqaciaacaGaaeqabaqabeGadaaakeaacqWGfbqrdaWgaaWcbaGaemODay3aaSbaaWqaaGGaciab=j7aInaaBaaabaGaeGymaedabeaaaeqaaaWcbeaaaaa@3251@| + |Evβ2
 MathType@MTEF@5@5@+=feaafiart1ev1aaatCvAUfKttLearuWrP9MDH5MBPbIqV92AaeXatLxBI9gBaebbnrfifHhDYfgasaacH8akY=wiFfYdH8Gipec8Eeeu0xXdbba9frFj0=OqFfea0dXdd9vqai=hGuQ8kuc9pgc9s8qqaq=dirpe0xb9q8qiLsFr0=vr0=vr0dc8meaabaqaciaacaGaaeqabaqabeGadaaakeaacqWGfbqrdaWgaaWcbaGaemODay3aaSbaaWqaaGGaciab=j7aInaaBaaabaGaeGOmaidabeaaaeqaaaWcbeaaaaa@3253@|)/2. In addition, the time complexity of all the swap operations considered at each step is *O *(|*E*|^2^). Therefore, the time complexity of SCCB-grid layout algorithm is *O *(|*E*|^2 ^+ |*U*||Evβ′
 MathType@MTEF@5@5@+=feaafiart1ev1aaatCvAUfKttLearuWrP9MDH5MBPbIqV92AaeXatLxBI9gBaebbnrfifHhDYfgasaacH8akY=wiFfYdH8Gipec8Eeeu0xXdbba9frFj0=OqFfea0dXdd9vqai=hGuQ8kuc9pgc9s8qqaq=dirpe0xb9q8qiLsFr0=vr0=vr0dc8meaabaqaciaacaGaaeqabaqabeGadaaakeaacqWGfbqrdaWgaaWcbaGaemODay3aaSbaaWqaaGGaciqb=j7aIzaafaaabeaaaSqabaaaaa@314C@| (|*V*| + |*E*|)) at each step.

Since the time complexity of CB-grid layout algorithm is *O *(|*V*|^2 ^+ |*E*|^2 ^+ |*W*||Evβ
 MathType@MTEF@5@5@+=feaafiart1ev1aaatCvAUfKttLearuWrP9MDH5MBPbIqV92AaeXatLxBI9gBaebbnrfifHhDYfgasaacH8akY=wiFfYdH8Gipec8Eeeu0xXdbba9frFj0=OqFfea0dXdd9vqai=hGuQ8kuc9pgc9s8qqaq=dirpe0xb9q8qiLsFr0=vr0=vr0dc8meaabaqaciaacaGaaeqabaqabeGadaaakeaacqWGfbqrdaWgaaWcbaGaemODay3aaSbaaWqaaGGaciab=j7aIbqabaaaleqaaaaa@3140@| (|*V*| + |*E*|)) at each step [15], the time complexity of SCCB-grid layout algorithm is *O*(|*V*||Evβ
 MathType@MTEF@5@5@+=feaafiart1ev1aaatCvAUfKttLearuWrP9MDH5MBPbIqV92AaeXatLxBI9gBaebbnrfifHhDYfgasaacH8akY=wiFfYdH8Gipec8Eeeu0xXdbba9frFj0=OqFfea0dXdd9vqai=hGuQ8kuc9pgc9s8qqaq=dirpe0xb9q8qiLsFr0=vr0=vr0dc8meaabaqaciaacaGaaeqabaqabeGadaaakeaacqWGfbqrdaWgaaWcbaGaemODay3aaSbaaWqaaGGaciab=j7aIbqabaaaleqaaaaa@3140@| (|*V*| + |*E*|)) larger than that of CB-grid layout algorithm (note that *v*_*β *_and *v*_*β' *_are not distinguished here). Here, we consider two cases, |*V*| ≤ |*W*| (case 1) and |*V*| > |*W*| (case 2) and show these two algorithms have the same time complexity with high probability. For case 1, the above difference is negligible since *O *(|*V*||Evβ
 MathType@MTEF@5@5@+=feaafiart1ev1aaatCvAUfKttLearuWrP9MDH5MBPbIqV92AaeXatLxBI9gBaebbnrfifHhDYfgasaacH8akY=wiFfYdH8Gipec8Eeeu0xXdbba9frFj0=OqFfea0dXdd9vqai=hGuQ8kuc9pgc9s8qqaq=dirpe0xb9q8qiLsFr0=vr0=vr0dc8meaabaqaciaacaGaaeqabaqabeGadaaakeaacqWGfbqrdaWgaaWcbaGaemODay3aaSbaaWqaaGGaciab=j7aIbqabaaaleqaaaaa@3140@| (|*V*| + |*E*|)) ≤ *O *(|*W*||Evβ
 MathType@MTEF@5@5@+=feaafiart1ev1aaatCvAUfKttLearuWrP9MDH5MBPbIqV92AaeXatLxBI9gBaebbnrfifHhDYfgasaacH8akY=wiFfYdH8Gipec8Eeeu0xXdbba9frFj0=OqFfea0dXdd9vqai=hGuQ8kuc9pgc9s8qqaq=dirpe0xb9q8qiLsFr0=vr0=vr0dc8meaabaqaciaacaGaaeqabaqabeGadaaakeaacqWGfbqrdaWgaaWcbaGaemODay3aaSbaaWqaaGGaciab=j7aIbqabaaaleqaaaaa@3140@|(|*V*| + |*E*|)). In contrast, the *O*(|*V*||Evβ
 MathType@MTEF@5@5@+=feaafiart1ev1aaatCvAUfKttLearuWrP9MDH5MBPbIqV92AaeXatLxBI9gBaebbnrfifHhDYfgasaacH8akY=wiFfYdH8Gipec8Eeeu0xXdbba9frFj0=OqFfea0dXdd9vqai=hGuQ8kuc9pgc9s8qqaq=dirpe0xb9q8qiLsFr0=vr0=vr0dc8meaabaqaciaacaGaaeqabaqabeGadaaakeaacqWGfbqrdaWgaaWcbaGaemODay3aaSbaaWqaaGGaciab=j7aIbqabaaaleqaaaaa@3140@| (|*V*| + |*E*|)) difference cannot be neglected in case 2. However, if we assume that all nodes can be moved to form the next layout with equal probability, |*V*||Evβ
 MathType@MTEF@5@5@+=feaafiart1ev1aaatCvAUfKttLearuWrP9MDH5MBPbIqV92AaeXatLxBI9gBaebbnrfifHhDYfgasaacH8akY=wiFfYdH8Gipec8Eeeu0xXdbba9frFj0=OqFfea0dXdd9vqai=hGuQ8kuc9pgc9s8qqaq=dirpe0xb9q8qiLsFr0=vr0=vr0dc8meaabaqaciaacaGaaeqabaqabeGadaaakeaacqWGfbqrdaWgaaWcbaGaemODay3aaSbaaWqaaGGaciab=j7aIbqabaaaleqaaaaa@3140@| = 2 |*E*|, and *O*(|*V*||Evβ
 MathType@MTEF@5@5@+=feaafiart1ev1aaatCvAUfKttLearuWrP9MDH5MBPbIqV92AaeXatLxBI9gBaebbnrfifHhDYfgasaacH8akY=wiFfYdH8Gipec8Eeeu0xXdbba9frFj0=OqFfea0dXdd9vqai=hGuQ8kuc9pgc9s8qqaq=dirpe0xb9q8qiLsFr0=vr0=vr0dc8meaabaqaciaacaGaaeqabaqabeGadaaakeaacqWGfbqrdaWgaaWcbaGaemODay3aaSbaaWqaaGGaciab=j7aIbqabaaaleqaaaaa@3140@| (|*V*| + |*E*|)) = *O *(|*V*|^2 ^+ |*E*|^2^) subsequently. Therefore, the time complexity of SCCB-grid layout algorithm will be the same as that of CB-grid layout algorithm even in the case 2. For the above reasons, the time complexities of SCCB-grid and CB-grid layout algorithms are the same in practice.

## Results and Discussion

### Data and Parameters

To evaluate our algorithms on a large-scale signal transduction pathway with a gene regulatory network, we create the pathway model of an endothelial cell with Cell Illustrator [1,2] by extracting information from [19]. The model consists of 309 nodes and 371 edges (three times as large as the apoptosis model in [15], which consists of 117 nodes and 126 edges), and the maximum degree of a node is ten (eight in the apoptosis model). Grid widths and heights are fixed to 100 pixels; the total numbers of vertical and horizontal grid points are 36 and 40, respectively. We used the following information pertaining to seven GO subcellular localizations: extracellular space (GO:0005615), cytoplasm (GO:0005737), nucleus (GO:0005634), mitochondrion (GO:0005739), plasma membrane (GO:0005886), nuclear membrane (GO:0005635), and mitochondria membrane (GO:0005740). We also used the following information pertaining to sixteen processes and entities used as attributes of nodes: migration, phosphorylation, protein with a modification, ligand, assembly, transcription, translation, mRNA, ligand and receptor, receptor, unknown, protein, exchange, trimer, ubiquitination, and degradation.

Usually, these types of biological models have many nodes termed as degradation. The degradation process always has only one edge. To exploit this property, we apply these layout algorithms after removing degradation nodes (97 nodes). After applying layout algorithms, we attach each eliminated degradation node just below the entity to which it was initially connected. Thus, in practice, the numbers of nodes and edges in the model given to layout algorithms are 212 and 274, respectively. Note that when the performances of algorithms are compared with the numbers of edge-edge crossings and node-edge crossings in the latter part of this section, crossings that are caused by degradations and edges connected to them are not taken into account.

We apply the following rule to edge-edge crossing weight *W*_*ee*_, node-edge crossing weight *W*_*ne*_, combo score weight *W*_*cs*_, and distance cost weight *W*_*dc *_of a layout cost, in Equation (2), to ensure that the importance of the distance cost is less than those of the others:

min⁡(Wee,Wne,Wcs)>Wdc⋅max⁡L∑v,u∈VDistancev,u(L).     (31)
 MathType@MTEF@5@5@+=feaafiart1ev1aaatCvAUfKttLearuWrP9MDH5MBPbIqV92AaeXatLxBI9gBaebbnrfifHhDYfgasaacH8akY=wiFfYdH8Gipec8Eeeu0xXdbba9frFj0=OqFfea0dXdd9vqai=hGuQ8kuc9pgc9s8qqaq=dirpe0xb9q8qiLsFr0=vr0=vr0dc8meaabaqaciaacaGaaeqabaqabeGadaaakeaacyGGTbqBcqGGPbqAcqGGUbGBcqGGOaakcqWGxbWvdaWgaaWcbaGaemyzauMaemyzaugabeaakiabcYcaSiabdEfaxnaaBaaaleaacqWGUbGBcqWGLbqzaeqaaOGaeiilaWIaem4vaC1aaSbaaSqaaiabdogaJjabdohaZbqabaGccqGGPaqkcqGH+aGpcqWGxbWvdaWgaaWcbaGaemizaqMaem4yamgabeaakiabgwSixpaaxababaGagiyBa0MaeiyyaeMaeiiEaGhaleaacqWGmbataeqaaOWaaabuaeaacqWGebarcqWGPbqAcqWGZbWCieGacqWF0baDcqWFHbqycqWFUbGBcqWGJbWycqWGLbqzdaWgaaWcbaGaemODayNaeiilaWIaemyDauhabeaakiabcIcaOiabdYeamjabcMcaPaWcbaGaemODayNaeiilaWIaemyDauNaeyicI4SaemOvayfabeqdcqGHris5aOGaeiOla4IaaCzcaiaaxMaadaqadaqaaiabiodaZiabigdaXaGaayjkaiaawMcaaaaa@6D3D@

In our study, *W*_*dc*_, *W*_*ee*_, *W*_*ne*_, and *W*_*cs *_were set to 1, 70, 150, and 110, respectively. Also, the constant C in *CW*_*a *_was set to 12.

Using the combo score, many nodes can be aligned vertically. However, in many cases, the nodes cannot be moved once they have combo relations. Plasma membrane, nuclear membrane, and mitochondrial membrane are thin and torus shaped, thus, vertical alignments of the nodes on these subcellular localizations will not be of interest for users (e.g., the width of plasma membrane in our model is only two grids). Therefore, in this paper, we decided to ignore combo scores in plasma membrane, nuclear membrane, and mitochondrial membrane.

### Comparison of layouts

**Figure 6:**
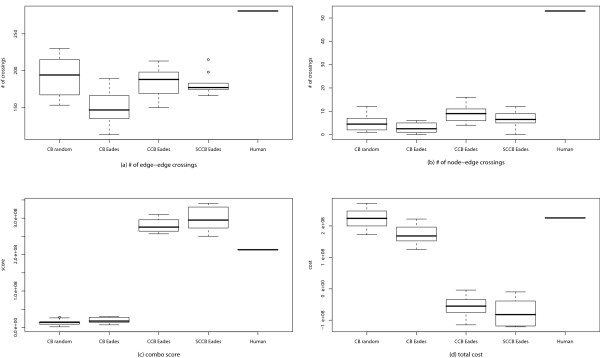
**Comparisons of edge-edge crossings, node-edge crossings, combo score, and total cost among the results of four grid layout algorithms and the human layout**. Costs and scores of the generated layouts with the CB random, CB Eades, CCB Eades, SCCB Eades, and human layout from the same initial layout. These algorithms are applied to ten initial layouts. (a) the number of edge-edge crossings. (b) the number of node-edge crossings. (c) combo score. (d) total cost.

**Figure 8:**
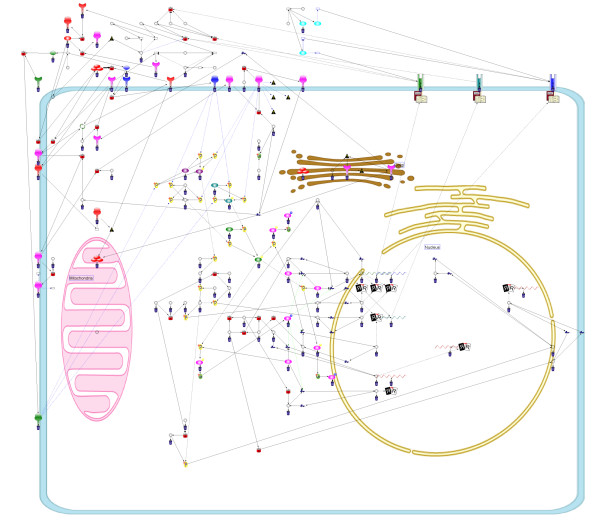
**A resulting layout of CB-grid layout algorithm**. A resulting layout of CB-grid layout algorithm in an endothelial signal transduction pathway. The pathway model is the same as that in Figure 10.

**Figure 9:**
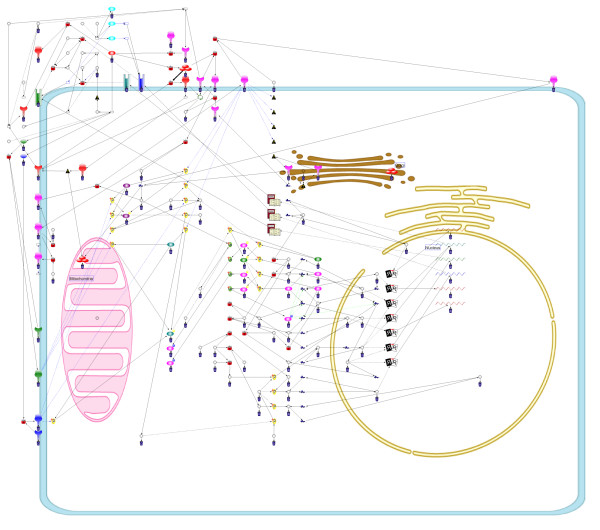
**A resulting layout of SCCB-grid layout algorithm**. A resulting layout of SCCB-grid layout algorithm in an endothelial signal transduction pathway. The pathway model is the same as that in Figure 10.

**Figure 10:**
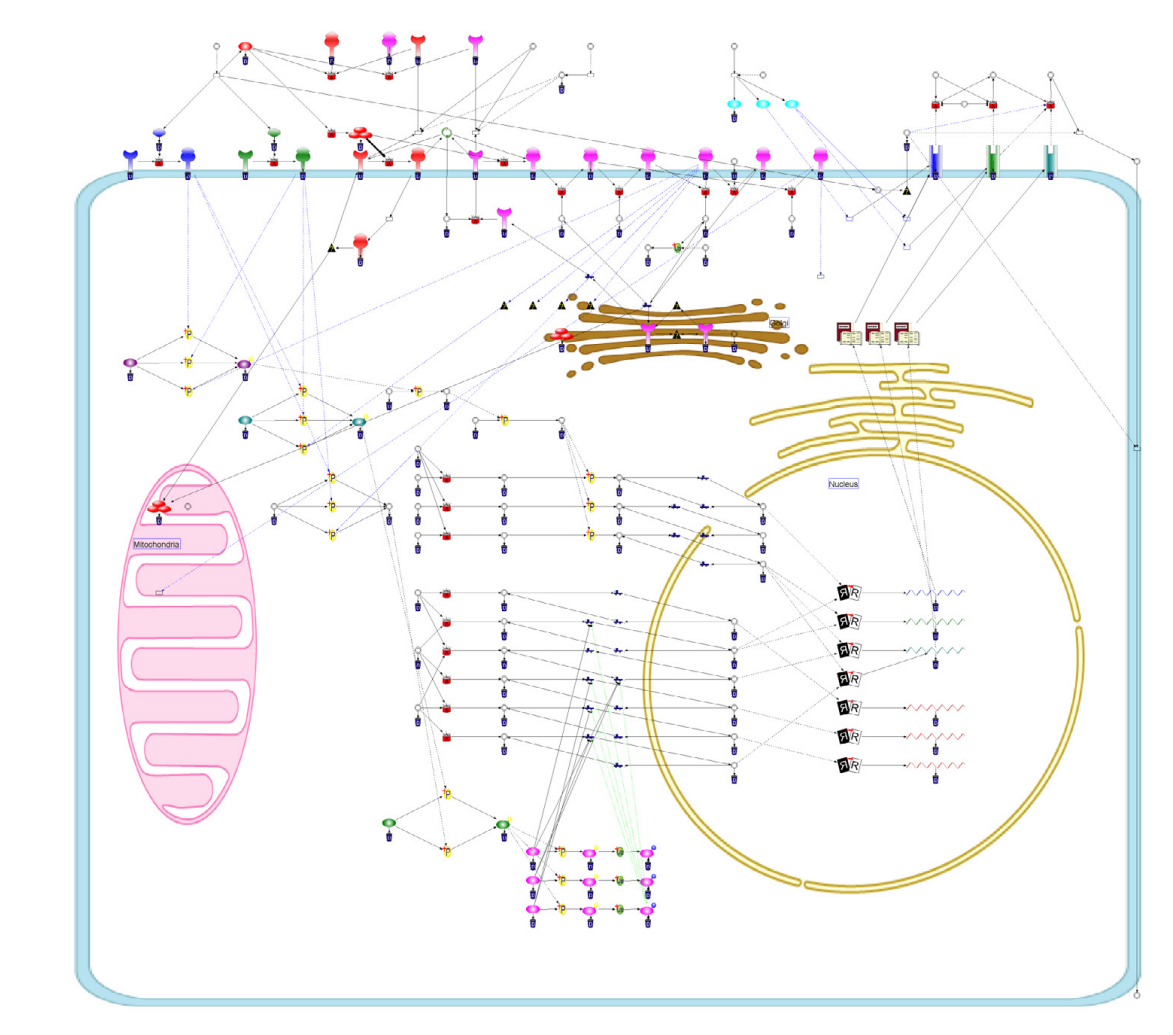
**The human layout**. The human layout of an endothelial signal transduction pathway. This pathway model is arranged with CB-grid and SCCB-grid layout algorithms in Figure 8 and Figure 9, respectively.

Figure 6 shows the number of edge-edge crossings, the number of node-edge crossings, combo scores, and total costs of the layouts with CB-grid, CCB-grid, and SCCB-grid layout algorithms, and the human layout. We generate ten initial layouts by applying Eades initial layout algorithm to ten random layouts. These initial layouts are commonly used for each layout algorithm (CB Eades, CCB Eades, and SCCB Eades in Figure 6). In addition, we use the ten random layouts directly as initial layouts of CB-grid layout algorithms (CB random in Figure 6, which corresponds to the previous layout algorithm) to confirm the significance of preparing proper initial layouts. Figure 8 and 9 respectively show the best layouts of CB-grid and SCCB-grid layout algorithms, which have the lowest total cost among ten resulting layouts of each algorithm. The human layout is shown in Figure 10.

**Figure 7:**
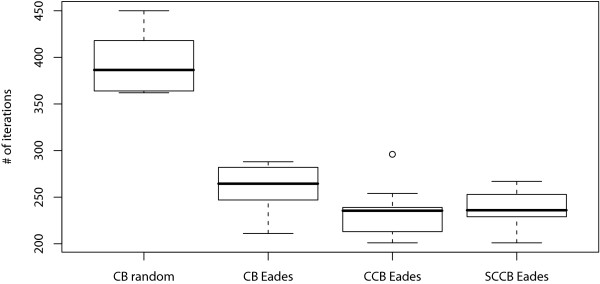
**Comparisons of the total numbers of iterations for optimal layouts among four grid layout algorithms**. Total number of iterations for optimal layouts with CB random, CB Eades, and SCCB Eades from the same initial layout. Ten initial layouts are applied with these algorithms.

In [15], the initial layout for CB-grid layout algorithm was a random layout, which had a large number of edge-edge crossings and node-edge crossings. Many iterations will, therefore, be needed until convergence. This fact prompted us to use the output of Eades initial layout algorithm as an initial layout. Figure 7 shows the number of iterations until convergence. As shown in this figure, CB-grid Eades successfully reduces the number of iterations when compared to CB-grid random (40% reduction on average). Moreover, the total score of CB-grid Eades is greatly improved over that of CB-grid random (see Figure 6(d)). A discussion in [15] was suggesting that reducing edge-edge crossings and node-edge crossings will lead to a better approximation of the human layout. In contrast as shown in Figure 6(a) and 6(b), the human layout also has several edge-edge and node-edge crossings, and has a higher combo score than that of CB-grid layout algorithm. Based on these facts, we proposed an additional scoring criterion – combo score – in CCB-grid layout algorithm. As seen through the value of combo scores (see Figure 6(c)), CCB-grid layout algorithm drastically improves this score, and this score becomes closer to that of the human layout. However, the numbers of edge-edge crossings and node-edge crossings in CCB-grid layout algorithm increase, comparing to CB-grid Eades (see Figure 6(a) and 6(b)). In this paper, the swap operation is proposed to increase the number of candidate layouts at each step. As shown in Figure 6(a) and 6(b), SCCB-grid layout algorithm succeeds in reducing edge-edge crossings and node-edge crossings, i.e., the above drawback of CCB-grid layout algorithm is partially diminished. In addition, as shown in Figure 6(c), the combo score of SCCB-grid layout algorithm is also improved slightly.

We also apply grid-layout algorithms to Fas-induced apoptosis pathway model [20] and ASE cell fate simulation model [21] to obtain a more generalized comparison. Resulting layouts and the number of crossings in each layout are summarized in Additional file 1. These models including the endothelial cell model are also available as Additional file 2, and the application of SCCB-grid layout algorithm for these models can be downloaded from [22].

## Conclusion

For better biopathway layouts, three improvements to CB-grid layout algorithm were proposed: (i) the improvement of initial layouts (ii) the improvement of cost function (iii) the improvement of search strategy itself without increasing the time complexity. For (i), Eades initial layout algorithm was proposed and the improvement was confirmed with a signal transduction pathway of an endothelial cell. For (ii), CCB-grid layout algorithm, which includes combo score function, was proposed and the improvement was verified with the same signal transduction pathway. For (iii), SCCB-grid layout algorithm was proposed. Due to (i) and (iii), our layout algorithm can be started from the better layout, and more robust to the condition of the initial layout than extant methods. In addition, we succeeded in utilizing the biological attributes that are not considered in extant methods due to combo score.

However, our layout algorithm has limitations and problems, which should be addressed in future work. Firstly, if the parameters of the combo score are not correctly selected, once a node gets a combo relation, the node no longer moves to other grid points anymore. Thus, it is important to devise a method that automatically selects the suitable parameters for the combo score function, edge-edge crossing function, and node-edge crossing function. Secondly, in our algorithm, only undirected graphs are considered to be laid out. On the other hand, for metabolic pathways, [11,13] proposed layout algorithms that decompose a digraph to hierarchical structural parts and directed cycle parts by considering the direction of edges in order to capture the flow of reactions. Therefore, the grid layout algorithm will also need to handle digraphs, utilizing its property that is effective especially in the grid-based layout. Finally, it should be addressed that grid layout algorithms including our new approach requires high time complexity and are not suitable for the real-time drawing. Thus, we would like to devise a further optimized grid layout algorithm to enable the real-time drawing.

## Authors' contributions

The basic idea was conceived by MK and MN. This idea was developed by KK and MN who then conceived a new idea and developed it. EJ created the endothelial model in Figure 10. SM supervised the whole study. The final manuscript was read and approved by all authors.

## Supplementary Material

Additional file 1 Resulting layouts of applying LK-grid layout algorithm, CB-grid layout algorithm and SCCB-grid layout algorithm to Fas-induced apoptosis pathway model and ASE cell fate simulation model are shown. Comparison of these results are also included. Click here for file

Additional file 2 Biopathway model files. Endothelial cell model, Fas-induced apoptosis pathway model and ASE cell fate simulation model are included. Click here for file

## References

[B1] Nagasaki M, Doi A, Matsuno H, Miyano S (2003). Genomic Object Net: I. A platform for modelling and simulating biopathways. Applied Bioinformatics.

[B2] Doi A, Nagasaki M, Fujita S, Matsuno H, Miyano S (2003). Genomic Object Net: II. Modelling biopathways by hybrid functional Petri net with extension. Applied Bioinformatics.

[B3] Shannon P, Markiel A, Ozier O, Baliga NS, Wang JT, Ramage D, Amin N, Schwikowski B, Ideker T (2003). Cytoscape: a software environment for integrated models of biomolecular interaction networks. Genome Research.

[B4] Networks/Pajek. http://vlado.fmf.uni-lj.si/pub/networks/pajek/.

[B5] Demir E, Babur O, Dogrusoz U, Gursoy A, Nisanci G, Atalay RC, Ozturk M (2002). PATIKA: an integrated visual environment for collaborative construction and analysis of cellular pathways. Bioinformatics.

[B6] Dogrusoz U, Erson EZ, Giral E, Demir E, Babur O, Cetintas A, Colak R (2006). PATIKAweb: a Web interface for analyzing biological pathways through advanced querying and visualization. Bioinformatics.

[B7] Kurata H, Matoba N, Shimizu N (2003). CADLIVE for constructing a large-scale biochemical network based on a simulation-directed notation and its application to yeast cell cycle. Nucleic Acids Research.

[B8] Kurata H, Masaki K, Sumida Y, Iwasaki R (2005). CADLIVE dynamic simulator: direct link of biochemical networks to dynamic models. Genome Research.

[B9] Brandes U, Dwyer T, Schreiber F (2003). Visualizing related metabolic pathways in two and a half dimensions. Proceedings of the 11th International Symposium on Graph Drawing.

[B10] Karp PD, Paley SM (1994). Automated drawing of metabolic pathways. Proceedings of the 3rd International Conference on Bioinformatics and Genome Research.

[B11] Becker MY, Rojas I (2001). A graph layout algorithm for drawing metabolic pathways. Bioinformatics.

[B12] Sirava M, Schafer T, Eiglsperger M, Kaufmann M, Kohlgacher O, Bornberg-Bauer E, Lenhof HP (2002). BioMiner-modeling, analyzing, and visualizing biochemical pathways and networks. Bioinformatics.

[B13] Wegner K, Kummer U (2005). A new dynamical layout algorithm for complex biochemical reaction networks. BMC Bioinformatics.

[B14] Li W, Kurata H (2005). A grid layout algorithm for automatic drawing of biochemical networks. Bioinformatics.

[B15] Kato M, Nagasaki M, Doi A, Miyano S (2005). Automatic drawing of biological networks using cross cost and subcomponent data. Genome Informatics.

[B16] Genc B, Dogrusoz U (2003). A constrained, force-directed layout algorithm for biological pathways. Proceedings of the 11th International Symposium on Graph Drawing.

[B17] Dogrusoz U, Gral E, Cetintas A, Civril A, Demir E (2004). A compound graph layout algorithm for biological pathways. Proceedings of the 12th International Symposium on Graph Drawing.

[B18] Eades P (1984). A heuristic for graph drawing. Congressus Nemerantium.

[B19] Pober JS (2002). Endothelial activation: Intracellular signaling pathways. Arthritis Research.

[B20] Matsuno H, Tanaka Y, Aoshima H, Doi A, Matsui M, Miyano S (2003). Biopathways representation and simulation on hybrid functional Petri net. In Silico Biology.

[B21] Saito A, Nagasaki M, Doi A, Ueno K, Miyano S (2006). Cell fate simulation model of gustatory nuerons with microRNAs double-negative feedback loop by hybrid functional Petri net with extension. Genome Informatics.

[B22] http://www.csml.org/download/SCCBLayout_BMC_inst.exe.

