# Supplementary material for: An efficient grid layout algorithm for biological networks utilizing various biological attributes
Source: BMC Bioinformatics. 2007 Mar 6;8:76. doi: 10.1186/1471-2105-8-76 (PMC1821340; doi:10.1186/1471-2105-8-76)
Supplement: Additional file 1 — Resulting layouts of applying LK-grid layout algorithm, CB-grid layout algorithm and SCCB-grid layout algorithm to Fas-induced apoptosis pathway model and ASE cell fate simulation model are shown. Comparison of these results are also included. [file 1471-2105-8-76-S1.pdf]

# Supplement

Fas-induced apoptosis pathway model [1] and ASE cell fate simulation model [2] are used to compare the performances of gird layout algorithms, LK-gird layout algorithm, CB-grid layout algorithm, and SCCB-grid layout algorithm. The initial layout of each model is obtained by using Eades initial layout algorithm, and then the above algorithms are applied to the initial layouts. The initial and resulting layouts of each model are shown in Figure 1 and Figure 2. The numbers of edge-edge crossings and node-edge crossings in the initial and resulting layouts of each model are described in Table 1 and Table 2, respectively. As is shown in Figures 1(b) and 2(b), nodes are separated to several clusters in the resulting layouts of LK-grid layout algorithm and the layouts look compact. However, theses layouts actually have lots of crossings, and especially the number of node-edge crossings is huge comparing to those of other two grid layout algorithms.

As is shown in Figures 1 and 2, the resulting layouts of CB-grid layout algorithm and SCCB-grid layout algorithm have major difference in the number of alignments nodes having the same attribute. These alignments are circled with blue boxes.

Table 1: The numbers of edge-edge crossings and node-edge crossings in layout for Fas-induced apoptosis pathway model.

| | Initial layout | LK | CB | SCCB |
|---|---|---|---|---|
| # of edge-edge crossings | 63 | 37 | 10 | 7 |
| # of node-edge crossings | 39 | 29 | 0 | 0 |

Table 2: The numbers of edge-edge crossings and node-edge crossings in layout for ASE cell fate simulation model.

| | Initial layout | LK | CB | SCCB |
|---|---|---|---|---|
| # of edge-edge crossings | 75 | 22 | 9 | 7 |
| # of node-edge crossings | 52 | 25 | 0 | 0 |

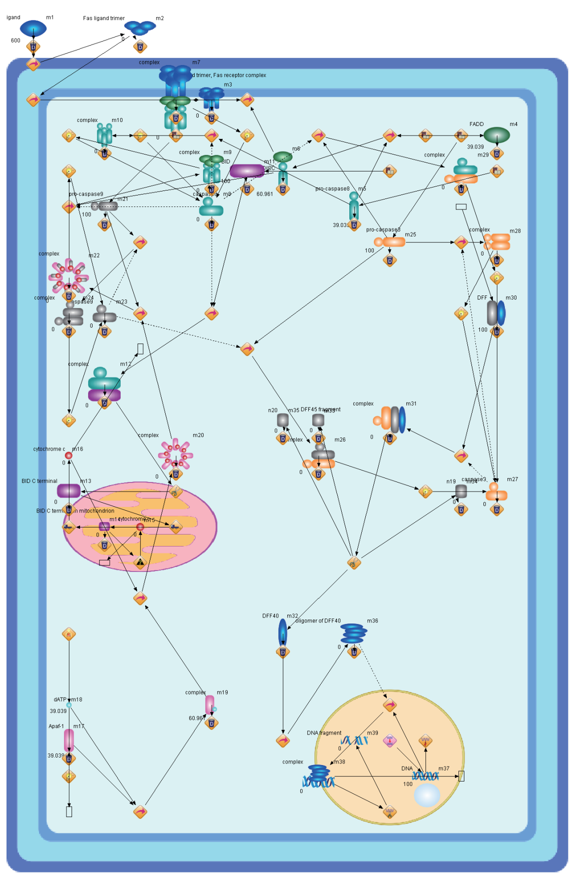

(a) Initial layout

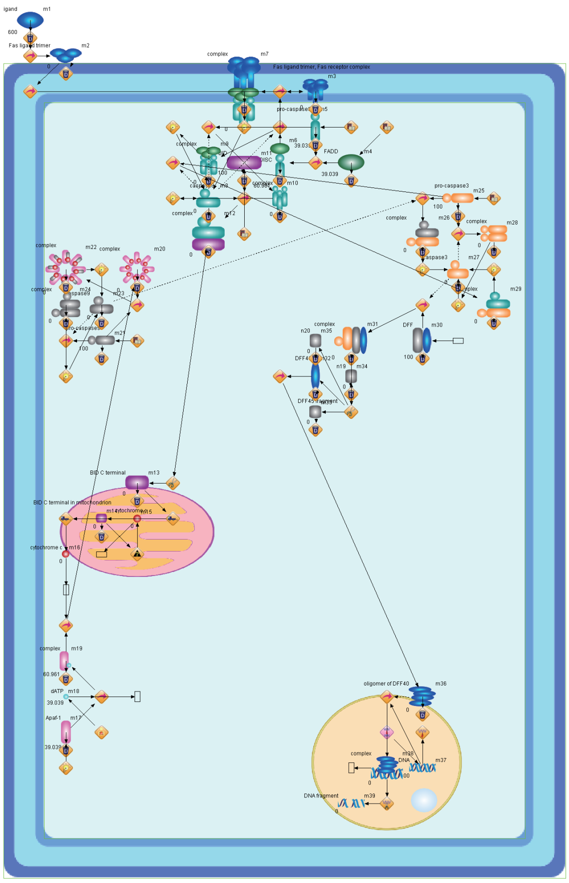

(b) LK-grid layout algorithm

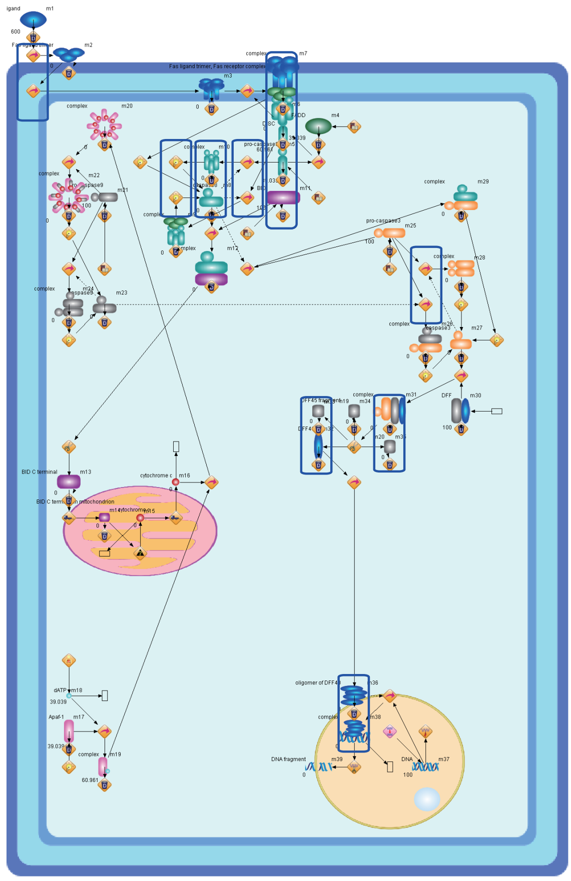

(c) CB-grid layout algorithm

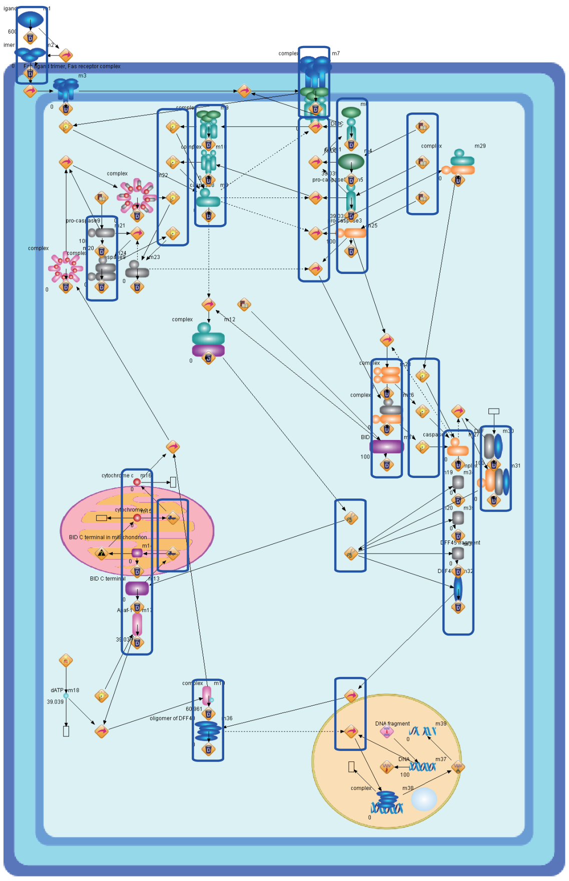

(d) SCCB-grid layout algorithm

Figure 1: Fas-induced apoptosis pathway model. This model consists of 117 nodes. (a) is the initial layout. (b), (c), (d) are resulting layouts of LK-grid layout algorithm, CB-grid layout algorithm, and SCCB-grid layout algorithm, respectively.

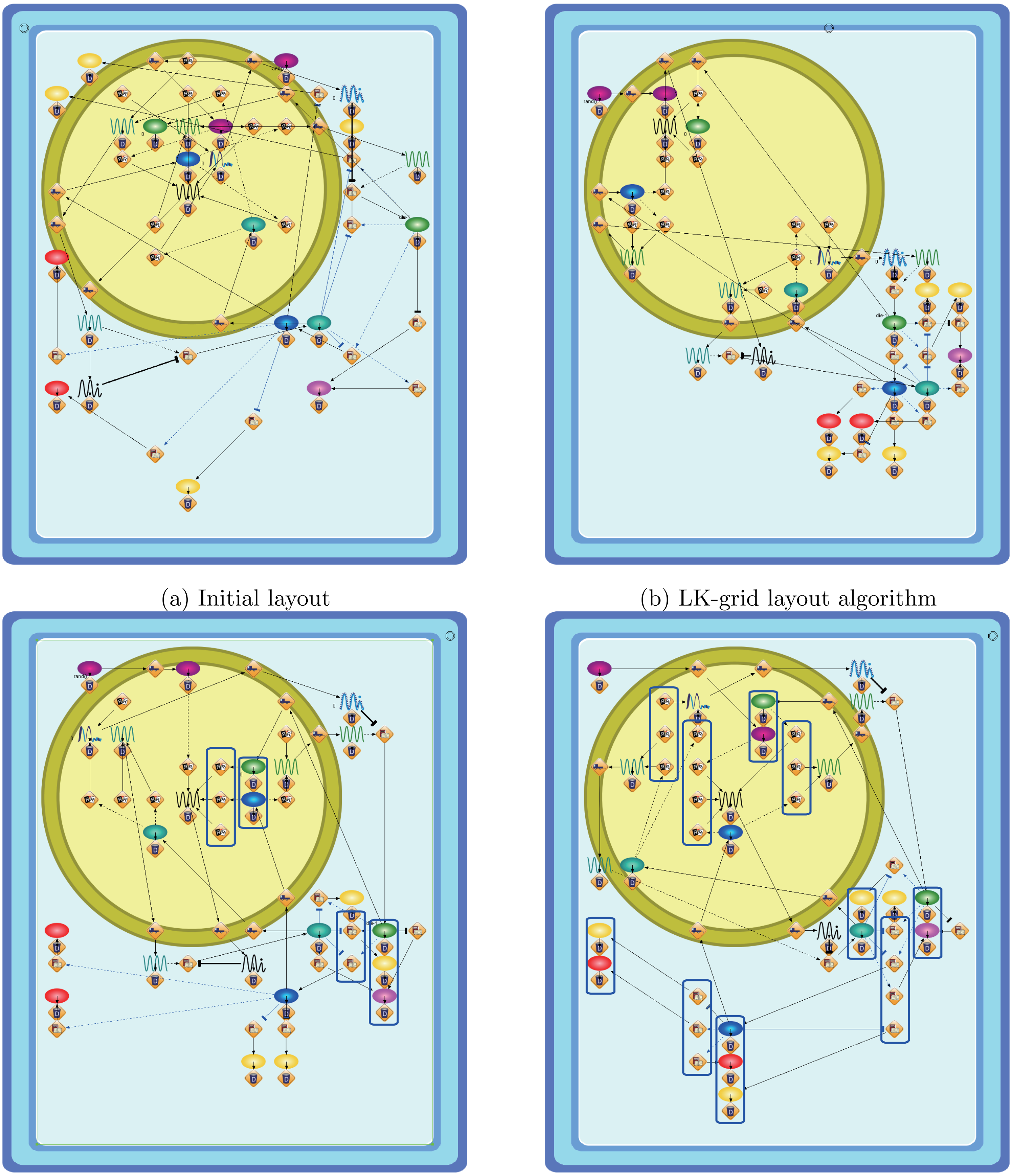

(a) Initial layout

(b) LK-grid layout algorithm

(c) CB-grid layout algorithm

(d) SCCB-grid layout algorithm

Figure 2: ASE cell fate simulation model. This model consists of 76 nodes. (a) is the initial layout. (b), (c), (d) are resulting layouts of LK-grid layout algorithm, CB-grid layout algorithm, and SCCB-grid layout algorithm, respectively.

## References

[1] Matsuno, H., Tanaka, Y., Aoshima, H., Doi, A., Matsui, M., Miyano, S., Biopathways Representation and Simulation on Hybrid Functional Petri Net, *In Silico Biology*, 3(3): 389–404, 2003.

[2] Saito, A., Nagasaki, M., Doi, A., Ueno, K., Miyano, S., Cell Fate Simulation Model of Gustatory Neurons with microRNAs Double-Negative Feedback Loop by Hybrid Functional Petri Net with Extension, *Genome Informatics* 17:100–111, 2006.
